# Ranking the dietary interventions by their effectiveness in the management of polycystic ovary syndrome: a systematic review and network meta-analysis

**DOI:** 10.1186/s12978-024-01758-5

**Published:** 2024-02-22

**Authors:** Anna Evelin Juhász, Márton Péter Stubnya, Brigitta Teutsch, Noémi Gede, Péter Hegyi, Péter Nyirády, Ferenc Bánhidy, Nándor Ács, Réka Juhász

**Affiliations:** 1 Center for Translational Medicine, Semmelweis University, Budapest, Hungary; 2 Department of Dietetics and Nutrition Sciences, Semmelweis University, Budapest, Hungary; 3 Department of Obstetrics and Gynaecology, Semmelweis University, Budapest, Hungary; 4 Institute for Translational Medicine, Medical School, University of Pécs, Pécs, Hungary; 5 Institute of Pancreatic Diseases, Semmelweis University, Budapest, Hungary; 6 Department of Urology, Semmelweis University, Budapest, Hungary

**Keywords:** Diet, Insulin resistance, Obesity, And weight loss

## Abstract

**Introduction:**

Polycystic ovary syndrome (PCOS) is a common condition in women, characterised by reproductive and metabolic dysfunction. While dietary approaches have been evaluated as a first-line treatment for patients with PCOS, there is limited evidence to support preference for a specific dietary composition. This systematic review and network meta-analysis was performed with the objective of comparing different dietary interventions in terms of positive impact. Metformin, the currently preferred treatment, was also compared.

**Methods:**

The latest systematic search was performed on the 20th of March, 2023. Eligible randomised controlled trials (RCTs) included patients with PCOS and compared the dietary approach with another intervention or a standard diet. Outcomes were expressed via anthropometric measurements and hormonal, glycemic, and lipid levels. The Bayesian method was used to perform a network meta-analysis and to calculate the surface under the cumulative ranking curve (SUCRA) values in order to rank the dietary interventions. The overall quality of the evidence was assessed using the Grading of Recommendations Assessment, Development, and Evaluation system.

**Results:**

19 RCTs were identified, comprising data from 727 patients who were variously treated with 10 types of dietary interventions and metformin. The Dietary Approaches to Stop Hypertension (DASH) diet was the most effective in reducing Homeostatic Model Assessment of Insulin Resistance (SUCRA 92.33%), fasting blood glucose (SUCRA 85.92%), fasting insulin level (SUCRA 79.73%) and triglyceride level (SUCRA 82.07%). For body mass index (BMI), the most effective intervention was the low-calorie diet (SUCRA 84.59%). For weight loss, the low-calorie diet with metformin (SUCRA 74.38%) was the most effective intervention. Metformin produced the greatest reductions in low-density lipoprotein cholesterol (SUCRA 78.08%) and total testosterone levels (SUCRA 71.28%). The low-carb diet was the most effective intervention for reducing cholesterol levels (SUCRA 69.68%), while the normal diet (SUCRA 65.69%) ranked first for increasing high-density lipoprotein cholesterol levels.

**Conclusion:**

Dietary interventions vary in their effects on metabolic parameters in women with PCOS. Based on our results, the DASH diet is the most effective dietary intervention for treating PCOS.

*Registration* PROSPERO ID CRD42021282984

**Supplementary Information:**

The online version contains supplementary material available at 10.1186/s12978-024-01758-5.

## Introduction

Polycystic ovarian syndrome (PCOS) is one of the most common endocrine diseases in women of reproductive age. Depending on the population studied and the diagnostic criteria used, prevalence ranges from 5 to 18%. [1]. The Rotterdam Criteria, established in 2003, is the most widely used, based on which the diagnosis of PCOS can be declared if at least 2 of the following 3 conditions are met: 1. Oligo-/amenorrhea or anovulation; 2. Laboratory- or clinically-proven hyperandrogenism; 3. A polycystic ovary is visible on an ultrasound image. Common symptoms of the syndromic disorder include: hyperandrogenism, hirsutism, irregular menstruation cycle, and infertility [2], causing significant reduction in quality of life for affected women [3]. Although the specific causes of PCOS are still unknown, insulin resistance (IR) has been identified as a significant etiological factor. Due to abnormal insulin receptor function and signaling, defective insulin receptor shape, or high amounts of insulin-binding antibodies, IR is associated with impaired insulin sensitivity in bodily tissues [4]. Additionally, obesity is commonly, though not always, observed in PCOS patients. It is also observed that being overweight increases the chance of developing PCOS [5].

Several interventions (pharmacological, nonpharmacological, or surgical) are available for reducing PCOS symptoms [6]. Metformin is an insulin sensitiser commonly used in PCOS patients with IR; however, gastrointestinal side effects limit its use as a first-choice for long-term treatment [7]. On the basis of recent results, inositols may have a beneficial effect on PCOS outcomes, but further research is needed [8, 9]. The International Evidence-based Guideline for the Assessment and Management of PCOS suggests dietary and exercise therapies as the first line of management [10]. The majority of women with PCOS are overweight or obese, and even a small weight reduction (5–10% of body weight) can significantly improve metabolic parameters and reproductive function. Weight reduction increases the sex hormone binding globulin (SHBG) concentration, improves ovarian function and fertility, and reduces miscarriages [11, 12].

Although achieving an ideal body weight improves symptoms in overweight women with PCOS, there is no clear evidence to determine which dietary intervention is best for achieving this goal [13, 14]. Currently, the most commonly used diet types are: low-calorie, low-carbohydrate, Dietary Approaches to Stop Hypertension (DASH), and Mediterranean diets for treating PCOS. This study aims to rank the effectiveness of the diets and treatment options used in the therapy of women with PCOS, by comparing anthropometric (e.g.: body weight) changes, hormonal (e.g.: total testosterone level) changes, and metabolic (e.g.: fasting blood sugar level, blood fat levels) changes measured during the intervention period. The main goal of the study is to provide clinicians with clear, evidence-based information about dietary interventions in lifestyle management in women with PCOS.

## Methods

This systematic review and network meta-analysis (NMA) is based on the PRISMA 2020 guideline and Cochrane *Handbook* recommendation [15]. The study protocol was registered on PROSPERO (CRD42022329961), and is adhered to fully.

### Eligibility criteria

The inclusion criteria specified any RCTs that included women with diagnosed polycystic ovary syndrome, and that compared two dietary approaches or one dietary approach to either a normal diet or metformin. Included RCTs reported at least one of the following outcomes: BMI, weight, total testosterone (TT), follicle-stimulating hormone (FSH), luteinizing hormone (LH), fasting blood glucose (FBG), fasting insulin (FI), HOMA-IR, total cholesterol (TC), TG, HDL, and LDL. Studies were excluded if patients performed exercise alongside their diet or were given dietary supplements to avoid potential effect modifiers.

### Information sources

Our systematic search was conducted on the 2nd of May, 2022. It was updated on the 8th of March, 2023, in five scientific databases: MEDLINE (via PubMed), Embase, Cochrane Central Register of Controlled Trials (CENTRAL), Scopus, and Web of Science. No language or other filters were applied.

### Search strategy

During the systematic search, the main concept was (PCOS) AND (dietary interventions) AND (metformin) AND random*. In order to increase the number of potentially relevant articles, besides the free-text words, we used MeSH and EMTREE terms. The entire search key can be found in the Additional file 1: Material.

### Selection process

After duplicate studies had been removed, the remaining studies were examined by title and abstract and then by full text by two independent authors (AEJ, MPS). To assess the rate of agreement between two raters at the two stages of the selection process (title and abstract and full text), we calculated Cohen’s kappa coefficient [16]. The third review author (BT) settled any disputes. The reference lists of additional articles were located via a manual search of the full texts of the eligible articles (RJ).

### Data collection

Data were independently collected by the two authors from the eligible articles (AEJ, MPS). All conflicts were resolved by a third independent author (RJ). The following data were extracted from each eligible article: the first author, the year of publication, study population, study period, country, number of centres, patient characteristics (age and BMI), number of patients allocated to the study arms, the types of dietary interventions used in the intervention and control groups, pre- and post-interventional values, and the change of the laboratory parameters according to our outcomes. In any case that required data were not completely available for a given article, the corresponding authors were contacted. Not all the dietary approaches were defined; for this reason, we created larger intervention groups based on the percentage of macronutrients. The different dietary approaches were standardised according to European Food Safety Authority (EFSA) Dietary Reference Values for Nutrients [17]. A normal diet was considered to be a daily energy intake of 55% carbohydrate, 15–20% protein, and 25–30% fat. The details of the dietary interventions can be found in Additional file 1: Table S2.

### Risk of bias assessment and quality of evidence

The risk of bias assessment was conducted in duplicate (AEJ, MPS) using Version 2 of the Cochrane Risk of Bias (RoB 2) Tool for all outcomes [18]. The five key domains evaluated were the randomisation process, deviation from the intended intervention, missing outcome data, outcome measurement, and selection of the reported results. Assigned to these domains were the categorisations: “low risk,” “some concerns”, or “high risk of bias.” Any disagreements among the assessors were resolved by a third review author (RJ).

We followed the GRADE (Grading of Recommendations Assessment, Development, and Evaluation) recommendation when evaluating the certainty of the evidence [19]. Two independent review authors (AEJ, MPS) evaluated each assessment criterion for each outcome and comparison. Any disputes were settled by a third party (RJ).

### Synthesis methods

Network meta-analyses was conducted with the random effects model using a Bayesian method [20]. The consistency examination was ruled out by a visual inspection of plots. The network is depicted in a graph, with the nodes representing diet types and the edges representing direct comparisons. The size of the node is related to the number of studies. The thickness of the edges is proportional to the number of trials with a direct comparison. Mean difference (MD) was used for continuous data with 95% credible intervals (the interpretation of the Bayesian 95% confidence interval) (95% CrI). The model was optimised and posterior samples generated using the Monte-Carlo methods running in four chains. At least 20.000 adaptation iterations were set for convergence, and 40.000 simulation iterations. The network estimates (pooled estimates of direct and indirect data) of each intervention were presented in comparison with each other in a league table. The surface under the cumulative ranking (SUCRA) curve values was used to rank the interventions according to their posterior probability. The cumulative probabilities of each treatment were expressed by a single value between 0 and 100%. Ranking probabilities allow for easy-to-interpret conclusions (“Intervention A has a 55% probability of being the best”). The probability that the intervention will be in the top rank or in one of the top ranks increases with a higher percentage or SUCRA value [21]. All calculations were performed with R (V. 4.1.1) package BUGSnet (V. 1.1.0), along with the Markov Chain Monte Carlo engine JAGS (V. 4-12).

Due to the small number of publications, a separate network meta-analysis for FSH and LH outcomes was not possible. The FSH and LH levels results presented in these articles are included only in the systematic review part. The qualitative synthesis also includes studies that only provided data as medians and did not report SD.

## Results

### Search and selection

**Fig. 1:**
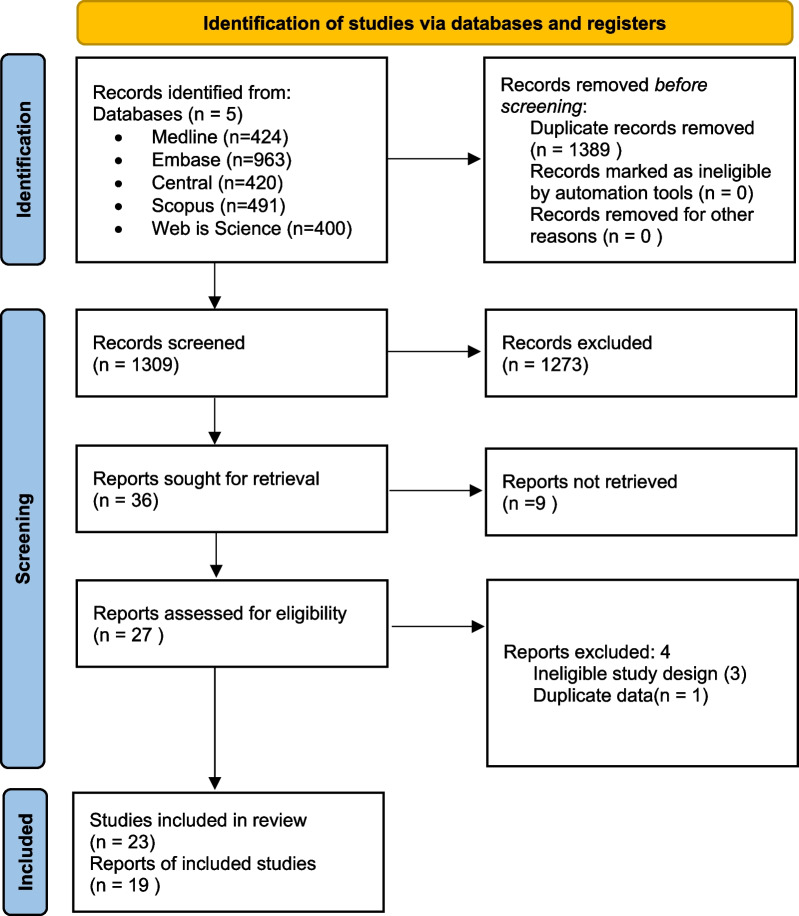
PRISMA 2020 flowchart representing the study selection process

A total of 1309 studies were identified as a result of the systematic search. 27 studies were found to be eligible for the qualitative and quantitative synthesis after removal of duplicate records. The selection process is shown in Fig. 1.

### Main characteristics of included studies

The baseline characteristics of the articles in the quantitative and qualitative synthesis are detailed in Table 1. The total number of involved patients is 800. The study duration varied from 4 to 24 weeks. As interventions, 10 dietary approaches and metformin were assessed in the trials.

**Table 1:** Basic characteristics of the included studies

First author, year	Country	Study period (weeks)	Number of patients	Mean age (SD) Intervention /Control group	BMI kg/m^2^ (SD) Intervention /Control group	Medication^1^	Intervention	Control	Outcome
Agowska, 2021 [48]	Poland	8	35	16.8 (1.3)	> 95th percentile	No	Low-calorie	Metformin	Weight, FBG, FI, HOMA-IR
Asemi, 2014 [49]	Iran	8	48	30.7 (6.7)/29.4 (6.2)	29.1 (3.2)/31.5 (5.7)	No	DASH	Normal	BMI, Weight, FBG, FI, HOMA-IR
Asemi, 2015 [50]	Iran	8	48	22.1 (3.2)/24.7 (6.0)	30.3 (4.5)/28.6 (5.8)	No	DASH	Normal	LDL, HDL, TC, TG
Azadi-Yazdi, 2017 [51]	Iran	12	55	32.1 (5.9)/31.7 (6.2)	31.9 (4.1)/30.2 (3.2)	No	DASH	Normal	BMI, Weight, TT
Esfahanian, 2013 [39]	Iran	12	30	20 (4.6)/21.9 (9.3)	34.1 (5.4)/31.1 (3.3)	No	Low-calorie	Metformin	BMI, FBG, FI, HOMA-IR, TT, LDL, HDL, TC, TG
Foroozanfard, 2017 [22]	Iran	12	60	27.1 (4.7)/25.6 (3.7)	32.3 (4.6)/32.2 (3.9)	N/A	DASH	Normal	BMI, Weight, FBG, FI, HOMA-IR, TT, FSH, LH
Galletly, 2007 [52]	Australia	16	27	33 (1.2)/32 (1.2)	37.6 (6.4)/34.5 (5.7)	No	Low-P	High-P	BMI, Weight
Gower, 2013 [23]	United States of America	8	30	31.2 (5.8)	31.8 (5.7)	No	Low-carb	Normal	FI, HOMA-IR, TT, FSH, LH, LDL, HDL, TC, TG
Marzouk, 2015 [53]	Egypt	24	60	19.3 (1.3)/20.1 (1.8)	36.0 (4.7)/35.8 (4.8)	No	Low-calorie	Normal	BMI, Weight, FBG
Mehrabani, 2012 [24]	Iran	12	49	28.5 (5.2)/30.5 (6.4)	31.1 (4.6)/31.9 (4.0)	No	Low-calorie	Normal	FI, HOMA-IR, TT, FSH, LH, LDL, HDL, TC, TG
Mei, 2022 [54]	China	12	59	27.9 (5.3)/28.07 (7.1)	39.3 (2.2)/29.5 (2.4)	No	Mediterranean	Low-fat	BMI
Moran, 2003 [55]	Australia	16	28	32 (1.2)/33 (1.2)	37.9 (1.6)/37.7 (1.9)	No	High-P	Low-P	FBG, FI, LDL, HDL, TC, TG
Nadjarzadeh, 2021 [56]	Iran	12	32	28.8 (6.5)/29.4 (6.6)	33.9 (5.3)/32.8 (5.3)	No	Low-calorie	High-P	BMI, Weight, TT
Panico, 2014 [27]	Italy	12	14	28.7 (4.9)	28.7 (4.9)	No	Low-GI	Normal	BMI, Weight, FBG, FI, HOMA-IR, TT, FSH, LH, TC, TG
Qublan, 2007 [25]	Jordan	24	46	31.5 (19–38)/30.8 (20–37)	32.2 (29–43)/31.9 (29–44)	No	High-P	Metformin	FBG, FI, FSH, LH
Sorensen, 2012 [57]	Denmark	24	27	27.7 (5.5)/28.4 (5.8)	30.6 (7.8)/30.5 (8.5)	No	High-P	Normal	BMI, Weight, FBG, TT, LDL, HDL, TC
Stamets, 2004 [26]	United States of America	4	26	29 (4)/26 (4)	38 (4)/37 (5)	No	High-P	Low-calorie	Weight, TT, FSH, LH, LDL, HDL, TC, TG
Toscani, 2011 [58]	Brazil	8	18	22.7 (5.6)/29.5 (5.7)	> 25 kg/m^2^	No	High-P	Low-calorie	Weight, FBG, LDL, HDL, TC
Wong, 2016 [59]	United States of America	24	16	15.4 (1.3)/16.3 (2.2)	36.2 (5.3)/33.9 (4.7)	No	Low-GI	Low-fat	BMI, FBG, FI, TT, TC, TG
Articles in the qualitative synthesis									
Mittal, 2020	India	12	21	33.1 (4.4)/34.4 (5.0)	33.7 (4.8)/32.2 (5.9)	No	Vegan	Low-calorie	BMI, Weight
Orstein, 2011	United States of America	12	16	15.8 (2.2)	35.7 (6)	No	Low-carbohydrate	Low-fat	Weight
Sordia-Hernández, 2015	Mexico	12	37	26.1 (4.1)/26.1 (4.7)	N/A	N/A	Low-glycemic	Normal	Weight
Turner-McGrievy, 2014	United States of America	24	18	27.8 (4.5)	39.9 (6.1)	No	Vegan	Low- calorie	Weight

N/A: not available; ^1^Medication that might affect the patients’ physiology during the intervention (lipid-lowering, anti-obesity, oral antidiabetic drug, hormonal therapy)

DASH, Dietary approaches to stop hypertension; Low-calorie + M, Low-calorie diet plus metformin; Low-carb, Low- carbohydrate diet; High-P, High-Protein diet; Low-GI, Low-Glycemic Index diet; Low-P, Low-Protein diet; FBG: fasting blood glucose; FI: fasting insulin; TG: triglyceride; TC: cholesterol; HDL: high-density lipoprotein cholesterol; LDL: low-density lipoprotein cholesterol; TT: total testosterone; FSH: follicle-stimulating hormone; LH: luteinizing hormone

### Quantitative synthesis

**Fig. 2:**
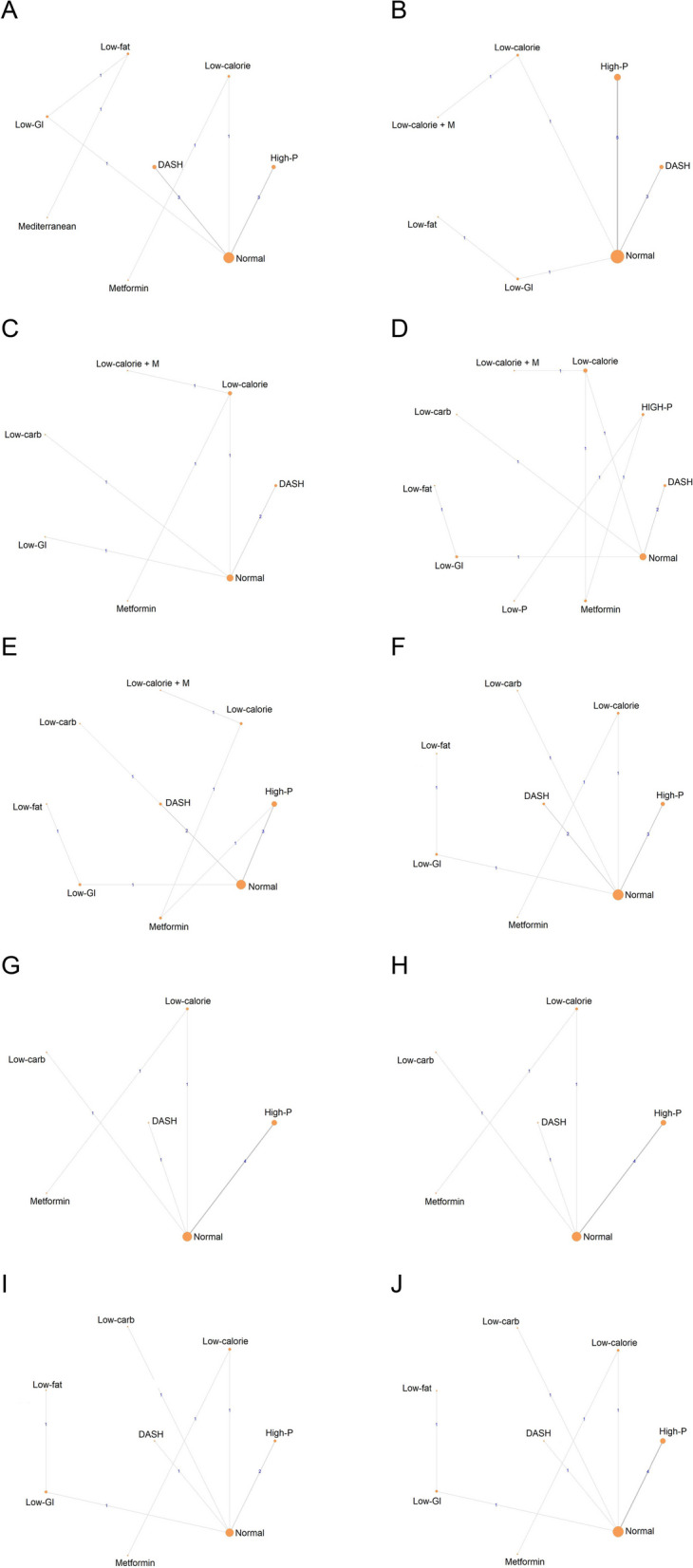
The network interventions regarding each outcomes. The size of the node is proportional to the number of studies. The thickness of the edges is proportional to the number of trials with a direct comparison. **A**: BMI, **B**: Weight, **C**: HOMA-IR, **D**: FI, **E**: FBG, **F**: TT, **G**: LDL, **H**: HDL, **I**: TG, **J**: TC. DASH, Dietary approaches to stop hypertension; Low-calorie + M, Low-calorie diet plus metformin; Low-carb, Low- carbohydrate diet; High-P, High-Protein diet; Low-GI, Low-Glycemic Index diet; Low-P, Low-Protein diet

The network interventions regarding each outcome are presented in Fig. 2.

### Ranking of interventions

Using ranking probabilities and the surface under the cumulative ranking curves, the relative ranking of the various dietary interventions for each outcome was calculated. The frequency of the different interventions must be considered since the Mediterranean and the low-protein diets had extreme SUCRA %, however, they were only in 1–1 RCTs. Results are shown in Table 2.

**Table 2:** Interventions’ SUCRA% regarding each outcomes

Dietary approaches		Anthropometric measurements		Glycemic factors			Lipid factors				Hormonal parameters	Summary ranking
		BMI	Weight	HOMA-IR	FBG	FI	TC	TG	HDL	LDL	TT	All outcomes combined
DASH	SUCRA %	57.1	53.3^2^	**80.4**^2^	**76.6**	**79.7**	61.8	**82.1**	61.8	44.4	54.5	64.8
High-P		29.6	17.9	–	50.0	39.3	43.9	53.6	23.6	54.9	18.4	36.8
Low-calorie		**73.6**	69.9	59.1	20.1	67.5	51.8	38.7	56.8	41.5	61.9	54.1
Low-carb		–	–	50.7	73.4	52.9	**69.7**	46.7	55.2	22.5	67.4	54.8
Low-fat		60.1	60.6		51.1	61.2	52.5	43.6	–	–	51.4	54.4
Low-GI		36.6	35.3	32.6	40.4	34.8	53.8	64.1	–	–	44.8	42.8
Low-P		–	–	–	–	16.0	–	–	–	–	–	16.0
Mediterranean		65.6	–	–	–	–	–	–	–	–	–	65.6
Low-calorie + M		–	**74.4**	59.6	31.4	64.5	–	–	–	–	–	57.5
Metformin		38.5	–	30.9	54.5	37.9	23.7	28.5	36.6	**78.1**	**71.2**	44.4
Normal		38.8	35.8	36.6	52.5	46.1	42.6	42.6	**65.7**	58.4	30.1	44.9

SUCRA values range from 0 to 100%. The higher the SUCRA value, and the closer to 100%, the higher the likelihood that intervention is in the top rank or one of the top ranks. The top interventions are in bold text. ^2^Means statistically significant difference was observed when the intervention was compared with normal diet

FBG: fasting blood glucose; FI: fasting insulin; TG: triglyceride; TC: cholesterol; HDL: high-density lipoprotein cholesterol; LDL: low-density lipoprotein cholesterol; TT: total testosterone;

DASH, Dietary approaches to stop hypertension; Low-calorie + M, Low-calorie diet plus metformin; Low-carb, Low- carbohydrate diet; High-P, High-Protein diet; Low-GI, Low-Glycemic Index diet; Low-P, Low-Protein diet

### Anthropometric measures

Regarding BMI, the network included 11 studies with 8 interventions and 428 patients (Fig. 2A). The low-calorie diet (SUCRA 73.58%) was ranked as the most effective intervention (Table 2, Additional file 1: Figure S1-S2). No statistically significant difference was observed in the change of BMI with the pairwise comparisons of the included different interventions in the network (Additional file 1: Table S3).

Regarding weight, the network 12 studies with 7 interventions and 418 patients (Fig. 2B). The low-calorie diet with metformin (SUCRA 73.7%) was ranked as the most effective intervention (Table 2, Additional file 1: Figure S3-S4). A statistically significant difference was observed when the low-fat diet was compared with low-GI (MD = − 3.59, 95% CrI: − 6.03; − 1.08); DASH diet with high-protein diet (MD = − 2.88 95% CrI: − 4.96; − 0.89) and with normal diet (MD = − 1.67 95% CrI: − 3.2; − 0.34) (Additional file 1: Table S4).

### Glycemic levels

Regarding HOMA-IR, the network included 7 studies and interventions with 286 patients (Fig. 2C). For this outcome, the DASH diet (SUCRA 80.47%) was the most effective intervention (Table 2, Additional file 1: Figure S5-S6). Furthermore, this type of diet produced a statistically significant difference compared to the normal diet (MD = − 1.10 95%-os CrI: − 2.05; − 0.03) in terms of HOMA-IR change (Additional file 1: Table S5).

Regarding FI level, the network included 10 studies and interventions with 376 patients (Fig. 2D). The DASH diet (SUCRA 79.73%) was the most effective based on the SUCRA values regarding FI level (Table 2, Additional file 1: Figure S7-8). There was no statistically significant difference between the interventions (Additional file 1: Table S6).

Regarding FBG level, the network included 11 studies, 9 interventions, and 372 patients (Fig. 2E). Based on the SUCRA values, the DASH diet (SUCRA 76.6%) was ranked the most effective dietary intervention for decreasing FBG levels (Table 2, Additional file 1: Figure S9-S10). No statistically significant difference was observed in the change of FBG with the pair-wise comparisons of the included different interventions in the network (Additional file 1: Table S7).

### Hormonal measures

Regarding total testosterone levels, the network included 10 studies, 8 interventions, and 359 patients (Fig. 2F). Metformin (SUCRA 71.28%) was observed to be the most effective based on the SUCRA values regarding total testosterone levels (Table 2, Additional file 1: Figure S11-S12). No significant difference could be established between the interventions (Additional file 1: Table S8).

### Lipid levels

The network of LDL and HDL levels included 8 studies, 6 interventions, and 276 patients (Fig. 2G, H). Metformin (SUCRA 78.08%) was the most effective intervention regarding LDL (Additional file 1: Figure S13-S14), while the normal diet (SUCRA 65.69%) was observed to be the most effective intervention regarding HDL levels (Additional file 1: Figure S14-S15). No statistically significant difference was observed in the change of LDL or HDL with the pairwise comparisons of the included interventions in the network (Additional file 1: Table S9–10).

Regarding TG level, 8 studies and interventions with 261 patients were included in the network (Fig. 2I). The DASH diet (SUCRA 82.07%) was the most effective intervention, and with the pair-wise comparisons of the included different interventions (Additional file 1: Figure S17-S18), no statistically significant difference was observed in the change of TG level (Additional file 1: Table S11).

Regarding TC level, the network included 10 studies and 8 interventions with 306 patients (Fig. 2J). For this outcome, the low-carb diet (SUCRA 69.68%) was the most effective intervention (Additional file 1: Figure S19-S20). No statistically significant difference was observed in the effects of the interventions (Additional file 1: Table S12).

### Qualitative synthesis

Ten studies were included in the qualitative synthesis. In six articles, there were insufficient data regarding FSH and LH level outcomes [22–27]. Most studies reported no significant differences in FSH and LH values after the treatment. Additionally, there were no significant differences between the intervention and control groups. In two RCTs, the weight and BMI changes were measured in terms of percentage, which was not used in statistical analysis [28, 29]. Mittal et al. demonstrated a significant change in weight and BMI in the vegan group. Ornstein et al. showed that there was a weight change, but it was not statistically significant. One article did not report SD value for weight after the intervention time [30]. The authors reported that there was a change in patients’ weight but it was not statistically significant. In the fourth article, the data were in median [31]. The results show that vegan participants lost significantly more weight than patients in the low-calorie diet group.

### Risk of bias assessment and certainty of the evidence

**Fig. 3:**
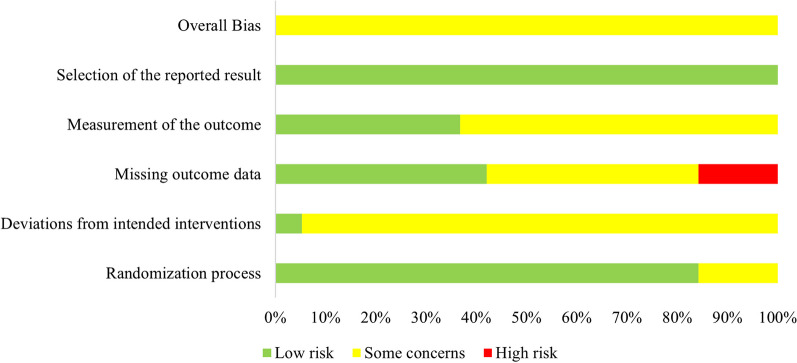
Overall quality of included studies

Risk of bias assessments reporting overall quality of included studies are presented in Fig. 3. Domain and study levels for each outcome separately can be found in the Additional file 1: Material. A majority of studies carried some concerns, while there were some high-risk points in 3 trials. The “missing outcome data” domain was considered as a high bias risk in these trials. Detailed results are found in Additional file 1: Figure S21-S40. For each comparison, the level of certainty of evidence ranged from low to high. Low certainty of evidence was mostly due to wide confidence intervals. Additional file 1: Table S13–23 contain the results of the GRADE assessment. No inconsistent results were indicated by the inconsistency test for any of the results. (Additional file 1: Figure S41-S49).

## Discussion

The purpose of this study was to create a ranking of the interventions used in treatment of PCOS and to identify which dietary intervention was most beneficial in regulating anthropometric, glycemic, lipid, and hormonal parameters in patients. Our NMA, including 19 RCTs with 708 patients, showed that different dietary interventions could influence anthropometric and metabolic parameters in PCOS.

Our results show that the DASH diet had a statistically significant, superior effect on reducing HOMA-IR and weight compared to the control normal diet, among the studied dietary interventions in women with PCOS. In measuring the severity of IR, HOMA-IR is a more reliable indicator than fasting insulin levels. Our results are consistent with a previous meta-analysis which included 19 trials (1193 women with PCOS) and indicated that the DASH diet and the calorie-restricted diet were likely to be optimal for reducing IR and improving body composition in women with PCOS. The DASH diet may have a positive impact on glycemic management by increasing β-cell function, decreasing high glucose and HbA1c levels, and improving insulin sensitivity [34–37]. Furthermore, the DASH diet provides high fibre intake, which is crucial for patients with metabolic disorders [38]. Various clinical trials have suggested that the DASH diet, either alone or combined with other lifestyle changes, can be effective in treating several diseases aside from hypertension [36].

We also found that a calorie-restricted diet is likely to be an effective option for losing weight. Low-calorie diets have been associated with weight loss that results in decreased fat mass and preserved lean body mass [39]. Abdominal fat has a strong association with insulin resistance, hyperandrogenism, and PCOS. Previous research has shown a correlation between the return of ovulation and the reduction of abdominal obesity. The improvement of metabolic and reproductive risk is significantly impacted by the observed reduction in abdominal fat [40].

Our analysis shows that the Mediterranean diet is an effective dietary intervention for reducing BMI. However, due to a limited number of studies, only one trial evaluated the effects of the Mediterranean diet.

For patients with IR, the recommendations prefer those diets in which the ratio of macronutrients is the same as that of normal dietary recommendations [42–44]. The beneficial effects of the balanced diets are proved in our NMA, since the high protein diet produced lower results (SUCRA 36.8%) compared to the normal diet (SUCRA 44.93%) or the DASH diet (SUCRA 64.78%). This is also supported by the fact that among the many dietary interventions, those which primarily aim to achieve desired results by changing the ratio of macronutrients (for example low-carbohydrate diet, low-fat diet) did not achieve a statistically significant difference compared to other dietary interventions.

In the treatment of PCOS, metformin is the currently preferred metabolic treatment [45]. The present NMA of drug therapy effectiveness could only examine a small group of cases (n = 39), and our results showed that metformin was the most effective intervention for decreasing total testosterone levels.

### Strengths and limitations

This study, to our knowledge, is the first network meta-analysis to rank the impact of dietary interventions in PCOS patients. The major strengths of this study are that it includes most of the relevant parameters in PCOS and benefits from a rigorous methodology. Furthermore, this study established an advantageous dietary intervention as a suggestion for clinicians in the treatment of PCOS. The main limitation of this study is the small number of direct comparisons and the low number of patients participating in the trials. The involved patients in the RCTs varied in age and ethnicity, to our knowledge, PCOS may present differently in different ethnicities and populations. In addition there is limited information in the included trials about the specific PCOS phenotype of the patients. PCOS phenotype may also influence the results. Another limitation in evaluating the data is presented by the different durations of the interventions. When analysing the results, it is important to consider each country's environment and food habits. DASH diet can be difficult to implement in some countries.

### Implications for practice and research

For the purposes of addressing new questions and providing greater clarity, more RCTs with larger case numbers are needed, and with longer follow-up times. With regard to informing practice, our results provide clear and useful guidance for clinicians concerning the beneficial effects of the DASH diet in the treatment of PCOS.

## Conclusion

It has been shown that implementing research results into everyday practice is essential and brings major health and economic benefits [46, 47]. Normalising weight and metabolic and hormonal parameters is important in treating PCOS but determining which dietary intervention should be preferred is complicated by a lack of clear evidence. Based on our results, the DASH diet should be preferred in the treatment of PCOS, especially in patients unable to tolerate the gastrointestinal side effects induced by metformin. In addition, a notable observation was that diets that avoid changing the ratio of macronutrients (for example, the DASH diet), and rather reduce the daily amount of calories and change the quality of the food, were generally more effective in reducing symptoms than those diets which aim to change the ratio of macronutrients (for example, a protein-rich diet).

### Supplementary Information

**Additional file 1: **PRISMA NMA Checklist; Searchkey; Summary of the dietary interventions; and Rankogram, Surface under the cumulative ranking (SUCRA) curves, League table, Risk of bias assessment, Assessment of certainty of evidence, Investigations of inconsistency of all outcomes.

## Data Availability

All data described in the manuscript, and data supporting the results, will be made publicly and freely available without restriction in the Additional file 1: material.

## Abbreviations

BMI: Body mass index
CrI: Credible intervals
DASH: Dietary approaches to stop hypertension
FBG: Fasting blood glucose
FI: Fasting insulin
FSH: Follicle stimulating hormone
GRADE: The Grading of Recommendations Assessment, Development, and Evaluation
LH: Luteinizing hormone
MD: Mean difference
NMA: Network meta-analysis
PCOS: Polycystic ovarian syndrome
RCT: Randomised controlled trial
RoB 2: Risk-of-bias tool for randomised trials
SUCRA: The surface under the cumulative ranking curve
TC: Total cholesterol
TT: Total testosterone
