# Supplementary material for: Ranking the dietary interventions by their effectiveness in the management of polycystic ovary syndrome: a systematic review and network meta-analysis
Source: Reprod Health. 2024 Feb 22;21:28. doi: 10.1186/s12978-024-01758-5 (PMC10885527; doi:10.1186/s12978-024-01758-5)

**Additional Material**

Title: Ranking the dietary interventions by their effectiveness in the management of polycystic ovary syndrome – a systematic review and network meta-analysis

Authors:

Anna Evelin Juhász^1,2^, Márton Péter Stubnya^1,3^, Brigitta Teutsch^1,4^, Noémi Gede^4^, Péter Hegyi^1,4,5^, Péter Nyirády^1,6^, Ferenc Bánhidy^1,3^, Nándor Ács^1,3^, Réka Juhász^1,2^

Affiliations

^1^Center for Translational Medicine, Semmelweis University, Budapest, Hungary

^2^Department of Dietetics and Nutrition Sciences, Semmelweis University, Budapest, Hungary

^3^Department of Obstetrics and Gynaecology, Semmelweis University, Budapest, Hungary

^4^Institute for Translational Medicine, Medical School, University of Pécs, Pécs, Hungary

^5^Institute of Pancreatic Diseases, Semmelweis University, Budapest, Hungary

^6^Department of Urology, Semmelweis University, Budapest, Hungary

Table of contents

[Table S1: PRISMA NMA Checklist of Items to Include When Reporting A Systematic Review Involving a Network Meta-analysis 5](#_Toc135639870)

[Searchkey: 9](#_Toc135639871)

[Table S2: Summary of the dietary interventions 10](#_Toc135639872)

[Figure S1: Rankogram 11](#_Toc135639873)

[Figure S2: Surface under the cumulative ranking (SUCRA) curves of BMI 12](#_Toc135639874)

[Table S3: League table 13](#_Toc135639875)

[Figure S3: Rankogram representing surface under the cumulative ranking curve (SUCRA%) values of reducing weight. 14](#_Toc135639876)

[Figure S4: Surface under the cumulative ranking (SUCRA) curves of weight. 15](#_Toc135639877)

[Table S4: League table containing comparisons regarding fasting blood glucose level. 16](#_Toc135639878)

[Figure S5: Rankogram representing surface under the cumulative ranking curve (SUCRA%) values of reducing HOMA-IR. 17](#_Toc135639879)

[Figure S6: Surface under the cumulative ranking (SUCRA) curves of HOMA-IR. 18](#_Toc135639880)

[Table S5: League table containing comparisons regarding HOMA-IR. 19](#_Toc135639881)

[Figure S7: Rankogram representing surface under the cumulative ranking curve (SUCRA%) values of reducing fasting insulin level. 20](#_Toc135639882)

[Figure S8: Surface under the cumulative ranking (SUCRA) curves of fasting insulin level. 21](#_Toc135639883)

[Table S6: League table containing comparisons regarding fasting insulin level. 22](#_Toc135639884)

[Figure S9: Rankogram representing surface under the cumulative ranking curve (SUCRA%) values of reducing fasting blood glucose level. 23](#_Toc135639885)

[Figure S10: Surface under the cumulative ranking (SUCRA) curves of fasting blood glucose level. 24](#_Toc135639886)

[Table S7: League table containing comparisons regarding fasting blood glucose level. 25](#_Toc135639887)

[Figure S11: Rankogram representing surface under the cumulative ranking curve (SUCRA%) values of reducing total testosterone level. 26](#_Toc135639888)

[Figure S12: Surface under the cumulative ranking (SUCRA) curves of total testosterone level. 27](#_Toc135639889)

[Table S8: League table containing comparisons regarding total testosterone level. 28](#_Toc135639890)

[Figure S13: Rankogram representing surface under the cumulative ranking curve (SUCRA%) values of reducing LDL level. 29](#_Toc135639891)

[Figure S14: Surface under the cumulative ranking (SUCRA) curves of LDL level. 30](#_Toc135639892)

[Table S9: League table containing comparisons regarding LDL level. 31](#_Toc135639893)

[Figure S15: Rankogram representing surface under the cumulative ranking curve (SUCRA%) values of HDL level. 32](#_Toc135639894)

[Figure S16: Surface under the cumulative ranking (SUCRA) curves of HDL level. 33](#_Toc135639895)

[Table S10: League table containing comparisons regarding HDL level. 34](#_Toc135639896)

[Figure S17: Rankogram representing surface under the cumulative ranking curve (SUCRA%) values of reducing triglyceride level. 35](#_Toc135639897)

[Figure S18: Surface under the cumulative ranking (SUCRA) curves of triglyceride level. 36](#_Toc135639898)

[Table S11: League table containing comparisons regarding triglyceride level. 37](#_Toc135639899)

[Figure S19: Rankogram representing surface under the cumulative ranking curve (SUCRA%) values of reducing cholesterol level. 38](#_Toc135639900)

[Figure S20: Surface under the cumulative ranking (SUCRA) curves of cholesterol level. 39](#_Toc135639901)

[Table S12: League table containing comparisons regarding cholesterol level. 40](#_Toc135639902)

[Figure S21: Risk of bias assessment at study level regarding BMI. 41](#_Toc135639903)

[Figure S22: Risk of bias assessment at domain level regarding BMI. 42](#_Toc135639904)

[Figure S23: Risk of bias assessment at study level regarding weight. 43](#_Toc135639905)

[Figure S24: Risk of bias assessment at domain level regarding weight. 44](#_Toc135639906)

[Figure S25: Risk of bias assessment at study level regarding HOMA-IR. 45](#_Toc135639907)

[Figure S26: Risk of bias assessment at domain level regarding HOMA-IR. 46](#_Toc135639908)

[Figure S27: Risk of bias assessment at study level regarding fasting insulin level. 47](#_Toc135639909)

[Figure S28: Risk of bias assessment at domain level regarding fasting insulin level. 48](#_Toc135639910)

[Figure S29: Risk of bias assessment at study level regarding fasting blood glucose level. 49](#_Toc135639911)

[Figure S30: Risk of bias assessment at domain level regarding fasting blood glucose level. 50](#_Toc135639912)

[Figure S31: Risk of bias assessment at study level regarding total testosterone level. 51](#_Toc135639913)

[Figure S32: Risk of bias assessment at domain level regarding total testosterone level. 52](#_Toc135639914)

[Figure S33: Risk of bias assessment at study level regarding LDL level. 53](#_Toc135639915)

[Figure S34: Risk of bias assessment at domain level regarding LDL level. 54](#_Toc135639916)

[Figure S35: Risk of bias assessment at study level regarding HDL level. 55](#_Toc135639917)

[Figure S36: Risk of bias assessment at domain level regarding HDL level. 56](#_Toc135639918)

[Figure S37: Risk of bias assessment at study level regarding triglyceride level. 57](#_Toc135639919)

[Figure S38: Risk of bias assessment at domain level regarding triglyceride level. 58](#_Toc135639920)

[Figure S39: Risk of bias assessment at study level regarding cholesterol level. 59](#_Toc135639921)

[Figure S40: Risk of bias assessment at domain level regarding cholesterol level. 60](#_Toc135639922)

[Table S13: Assessment of certainty of evidence regarding BMI. 61](#_Toc135639923)

[Table S14: Assessment of certainty of evidence regarding weight. 62](#_Toc135639924)

[Table S15: Assessment of certainty of evidence regarding HOMA-IR. 63](#_Toc135639925)

[Table S16: Assessment of certainty of evidence regarding fasting insulin level. 63](#_Toc135639926)

[Table S17: Assessment of certainty of evidence regarding fasting insulin level. 64](#_Toc135639927)

[Table S18: Assessment of certainty of evidence regarding fasting blood glucose level. 66](#_Toc135639928)

[Table S19: Assessment of certainty of evidence regarding total testosterone level. 67](#_Toc135639929)

[Table S20: Assessment of certainty of evidence regarding LDL level. 68](#_Toc135639930)

[Table S21: Assessment of certainty of evidence regarding HDL level. 69](#_Toc135639931)

[Table S22: Assessment of certainty of evidence regarding triglyceride level. 70](#_Toc135639932)

[Table S23: Assessment of certainty of evidence regarding cholesterol level. 71](#_Toc135639933)

[Figure S41: Results from investigations of inconsistency regarding BMI. 72](#_Toc135639934)

[Figure S42: Results from investigations of inconsistency regarding weight. 73](#_Toc135639935)

[Figure S43: Results from investigations of inconsistency regarding HOMA-IR. 74](#_Toc135639936)

[Figure S43: Results from investigations of inconsistency regarding fasting insulin level. 75](#_Toc135639937)

[Figure S44: Results from investigations of inconsistency regarding fasting blood glucose level. 76](#_Toc135639938)

[Figure S45: Results from investigations of inconsistency regarding total testosterone level. 77](#_Toc135639939)

[Figure S46: Results from investigations of inconsistency regarding LDL level. 78](#_Toc135639940)

[Figure S47: Results from investigations of inconsistency regarding HDL level. 79](#_Toc135639941)

[Figure S48: Results from investigations of inconsistency regarding triglyceride level. 80](#_Toc135639942)

[Figure S49: Results from investigations of inconsistency regarding cholesterol level. 81](#_Toc135639943)

# Table S1: PRISMA NMA Checklist of Items to Include When Reporting A Systematic Review Involving a Network Meta-analysis

| **Section/Topic** | **Item #** | **Checklist Item** | **Reported on Page #** |
| --- | --- | --- | --- |
| **TITLE** |  |  |  |
| Title | 1 | Identify the report as a systematic review *incorporating a network meta-analysis (or related form of meta-analysis).* | ***Page 1*** |
|  |  |  |  |
| **ABSTRACT** |  |  |  |
| Structured summary | 2 | Provide a structured summary including, as applicable:  **Background:** main objectives  **Methods:** data sources; study eligibility criteria, participants, and interventions; study appraisal; and *synthesis methods, such as network meta-analysis.*  **Results:** number of studies and participants identified; summary estimates with corresponding confidence/credible intervals; *treatment rankings may also be discussed. Authors may choose to summarize pairwise comparisons against a chosen treatment included in their analyses for brevity.*  **Discussion/Conclusions:** limitations; conclusions and implications of findings.  **Other:** primary source of funding; systematic review registration number with registry name. | Page 4 |
|  |  |  |  |
| **INTRODUCTION** |  |  |  |
| Rationale | 3 | Describe the rationale for the review in the context of what is already known*, including mention of why a network meta-analysis has been conducted.* | ***Page 6*** |
| Objectives | 4 | Provide an explicit statement of questions being addressed, with reference to participants, interventions, comparisons, outcomes, and study design (PICOS). | Page 7 |
|  |  |  |  |
| **METHODS** |  |  |  |
| Protocol and registration | 5 | Indicate whether a review protocol exists and if and where it can be accessed (e.g., Web address); and, if available, provide registration information, including registration number. | Page 8 |
| Eligibility criteria | 6 | Specify study characteristics (e.g., PICOS, length of follow-up) and report characteristics (e.g., years considered, language, publication status) used as criteria for eligibility, giving rationale. *Clearly describe eligible treatments included in the treatment network, and note whether any have been clustered or merged into the same node (with justification).* | ***Page 8*** |
| Information sources | 7 | Describe all information sources (e.g., databases with dates of coverage, contact with study authors to identify additional studies) in the search and date last searched. | Page9 |
| Search | 8 | Present full electronic search strategy for at least one database, including any limits used, such that it could be repeated. | Page 9 |
| Study selection | 9 | State the process for selecting studies (i.e., screening, eligibility, included in systematic review, and, if applicable, included in the meta-analysis). | Page 9 |
| Data collection process | 10 | Describe method of data extraction from reports (e.g., piloted forms, independently, in duplicate) and any processes for obtaining and confirming data from investigators. | Page 9 |
| Data items | 11 | List and define all variables for which data were sought (e.g., PICOS, funding sources) and any assumptions and simplifications made. | Page 9 |
| **Geometry of the network** | **S1** | Describe methods used to explore the geometry of the treatment network under study and potential biases related to it. This should include how the evidence base has been graphically summarized for presentation, and what characteristics were compiled and used to describe the evidence base to readers. | ***Page 8*** |
| Risk of bias within individual studies | 12 | Describe methods used for assessing risk of bias of individual studies (including specification of whether this was done at the study or outcome level), and how this information is to be used in any data synthesis. | Page 9 |
| Summary measures | 13 | State the principal summary measures (e.g., risk ratio, difference in means). *Also describe the use of additional summary measures assessed, such as treatment rankings and surface under the cumulative ranking curve (SUCRA) values, as well as modified approaches used to present summary findings from meta-analyses.* | Page 8 |
| Planned methods of analysis | 14 | Describe the methods of handling data and combining results of studies for each network meta-analysis. This should include, but not be limited to:   - *Handling of multi-arm trials;* - *Selection of variance structure;* - *Selection of prior distributions in Bayesian analyses; and* - *Assessment of model fit.* | Page 8 |
| **Assessment of Inconsistency** | **S2** | Describe the statistical methods used to evaluate the agreement of direct and indirect evidence in the treatment network(s) studied. Describe efforts taken to address its presence when found. | Page 10 |
| Risk of bias across studies | 15 | Specify any assessment of risk of bias that may affect the cumulative evidence (e.g., publication bias, selective reporting within studies). |  |
| Additional analyses | 16 | Describe methods of additional analyses if done, indicating which were pre-specified. This may include, but not be limited to, the following:   - Sensitivity or subgroup analyses; - Meta-regression analyses; - *Alternative formulations of the treatment network; and* - *Use of alternative prior distributions for Bayesian analyses (if applicable).* |  |
|  |  |  |  |
| **RESULTS†** |  |  |  |
| Study selection | 17 | Give numbers of studies screened, assessed for eligibility, and included in the review, with reasons for exclusions at each stage, ideally with a flow diagram. | Page 11 |
| **Presentation of network structure** | **S3** | Provide a network graph of the included studies to enable visualization of the geometry of the treatment network. | ***Page 15*** |
| **Summary of network geometry** | **S4** | Provide a brief overview of characteristics of the treatment network. This may include commentary on the abundance of trials and randomized patients for the different interventions and pairwise comparisons in the network, gaps of evidence in the treatment network, and potential biases reflected by the network structure. | ***Page 15*** |
| Study characteristics | 18 | For each study, present characteristics for which data were extracted (e.g., study size, PICOS, follow-up period) and provide the citations. | Page 13 |
| Risk of bias within studies | 19 | Present data on risk of bias of each study and, if available, any outcome level assessment. | Additional material |
| Results of individual studies | 20 | For all outcomes considered (benefits or harms), present, for each study: 1) simple summary data for each intervention group, and 2) effect estimates and confidence intervals. *Modified approaches may be needed to deal with information from larger networks.* | Additional material |
| Synthesis of results | 21 | Present results of each meta-analysis done, including confidence/credible intervals. *In larger networks, authors may focus on comparisons versus a particular comparator (e.g. placebo or standard care), with full findings presented in an appendix. League tables and forest plots may be considered to summarize pairwise comparisons.* If additional summary measures were explored (such as treatment rankings), these should also be presented. | Additional material |
| **Exploration for inconsistency** | **S5** | Describe results from investigations of inconsistency. This may include such information as measures of model fit to compare consistency and inconsistency models, *P* values from statistical tests, or summary of inconsistency estimates from different parts of the treatment network. | ***Supplementary*** |
| Risk of bias across studies | 22 | Present results of any assessment of risk of bias across studies for the evidence base being studied. | Page 20 |
| Results of additional analyses | 23 | Give results of additional analyses, if done (e.g., sensitivity or subgroup analyses, meta-regression analyses*, alternative network geometries studied, alternative choice of prior distributions for Bayesian analyses,* and so forth). |  |
|  |  |  |  |
| **DISCUSSION** |  |  |  |
| Summary of evidence | 24 | Summarize the main findings, including the strength of evidence for each main outcome; consider their relevance to key groups (e.g., healthcare providers, users, and policy-makers). | Page 21 |
| Limitations | 25 | Discuss limitations at study and outcome level (e.g., risk of bias), and at review level (e.g., incomplete retrieval of identified research, reporting bias). *Comment on the validity of the assumptions, such as transitivity and consistency. Comment on any concerns regarding network geometry (e.g., avoidance of certain comparisons).* | Page 23 |
| Conclusions | 26 | Provide a general interpretation of the results in the context of other evidence, and implications for future research. | Page 23 |
|  |  |  |  |
| **FUNDING** |  |  |  |
| Funding | 27 | Describe sources of funding for the systematic review and other support (e.g., supply of data); role of funders for the systematic review. This should also include information regarding whether funding has been received from manufacturers of treatments in the network and/or whether some of the authors are content experts with professional conflicts of interest that could affect use of treatments in the network. | ***Page 2*** |

PICOS = population, intervention, comparators, outcomes, study design.

* Text in italics indicateS wording specific to reporting of network meta-analyses that has been added to guidance from the PRISMA statement.

† Authors may wish to plan for use of appendices to present all relevant information in full detail for items in this section.

# Searchkey:

Pubmed:

(diet OR food OR "feeding behavior" OR "dietary pattern" OR "feeding pattern" OR "eating behavior" OR "food selection" OR "dietary habit" OR "dietary approach" OR "food habit" OR "eating habit" OR "intermittent fasting" OR "dietary intervention") AND (PCOS OR PCOD OR polycystic ovarian syndrome OR polycystic ovary syndrome OR "polycystic ovary") AND random*

Cochrane library:

(diet OR food OR "feeding behavior" OR "dietary pattern" OR "feeding pattern" OR "eating behavior" OR "food selection" OR "dietary habit" OR "dietary approach" OR "food habit" OR "eating habit" OR "intermittent fasting" OR "dietary intervention") AND (PCOS OR PCOD OR "polycystic ovarian syndrome" OR "polycystic ovary syndrome" OR "polycystic ovary") AND random*

Embase:

(diet OR food OR 'feeding behavior' OR 'dietary pattern' OR 'feeding pattern' OR 'eating behavior' OR 'food selection' OR 'dietary habit' OR 'dietary approach' OR 'food habit' OR 'eating habit' OR 'intermittent fasting' OR 'dietary intervention') AND (PCOS OR PCOD OR 'polycystic ovarian syndrome' OR 'polycystic ovary syndrome' OR 'polycystic ovary') AND random*

Scopus:

(diet OR food OR "feeding behavior" OR "dietary pattern" OR "feeding pattern" OR "eating behavior" OR "food selection" OR "dietary habit" OR "dietary approach" OR "food habit" OR "eating habit" OR "intermittent fasting" OR "dietary intervention") AND (PCOS OR PCOD OR "polycystic ovarian syndrome" OR "polycystic ovary syndrome" OR "polycystic ovary") AND random*

Web of Science:

(diet OR food OR "feeding behavior" OR "dietary pattern" OR "feeding pattern" OR "eating behavior" OR "food selection" OR "dietary habit" OR "dietary approach" OR "food habit" OR "eating habit" OR "intermittent fasting" OR "dietary intervention") AND (PCOS OR PCOD OR "polycystic ovarian syndrome" OR "polycystic ovary syndrome" OR "polycystic ovary") AND random*

# Table S2: Summary of the dietary interventions

| Dietary intervention | Carbohydrate % | Protein % | Fat % | Other information |
| --- | --- | --- | --- | --- |
| DASH | 50-55 | 15-20 | 25-30 |  |
| High-P | 40 | 30 | 30 |  |
| Low-calorie | 40-55 | 15-20 | 30-35 | 400-600 kcal/day deficit |
| Low-fat | 55 | 20 | 25 |  |
| Low-GI | 40-45 | 20-30 | 30-35 | GI < 45 |
| Mediterranean | 20 | 35-45 | 35-45 |  |
| Low-carb | 41 | 19 | 40 |  |
| Low-P | 55 | 15 | 30 |  |
| Metformin | 50 | 25 | 25 | 1000-2000 mg metformin/day |
| Low-calorie + M | 40-50 | 15-20 | 30-35 | 400-600 kcal/day deficit + 1000-2000 mg metformin/day |
| Normal | 55 | 15-20 | 25-30 |  |

DASH, Dietary approach stop hypertension; Low-calorie + M, Low-calorie diet plus metformin; Low-carb, Low- carbohydrate diet; High-P, High-Protein diet; Low-GI, Low-Glycemic Index diet; Low-P, Low-Protein diet

Figure S1: Rankogram representing surface under the cumulative ranking curve (SUCRA%) values of reducing BMI. SUCRA values range from 0 to 100%. The higher the SUCRA value and the closer to 100%, the higher the likelihood that intervention is in the top rank or one of the top ranks.

Low-GI, Low-Glycemic Index diet; High-P, High-Protein diet


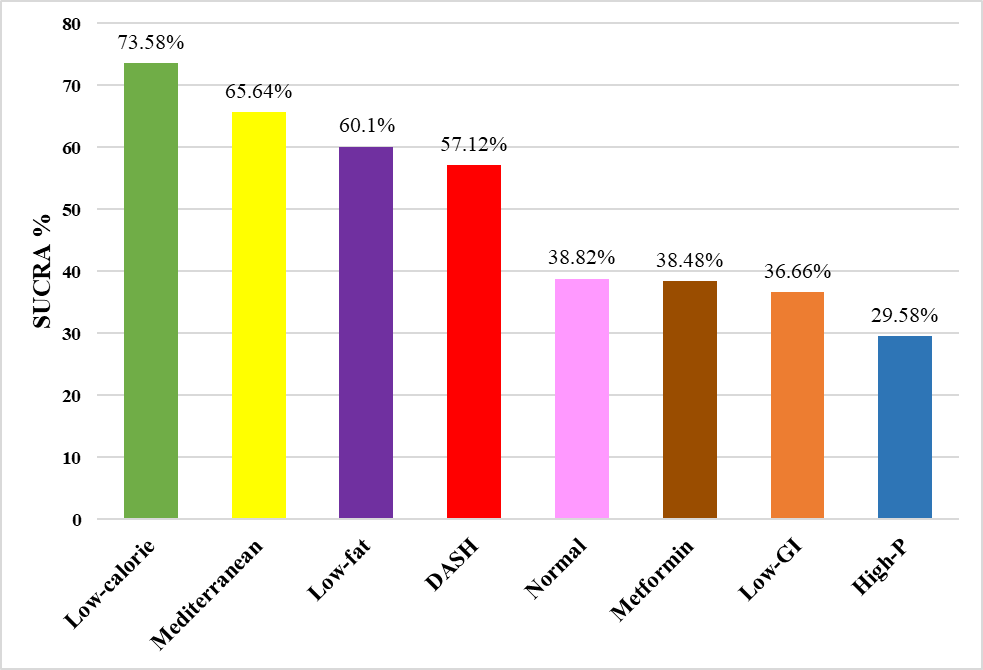


Figure S2: Surface under the cumulative ranking (SUCRA) curves of BMI. SUCRA values range from 0 to 100%. The higher the SUCRA value, and the closer to 100%, the higher the likelihood that intervention is in the top rank or one of the top ranks.

Low-GI, Low-Glycemic Index diet; High-P, High-Protein diet


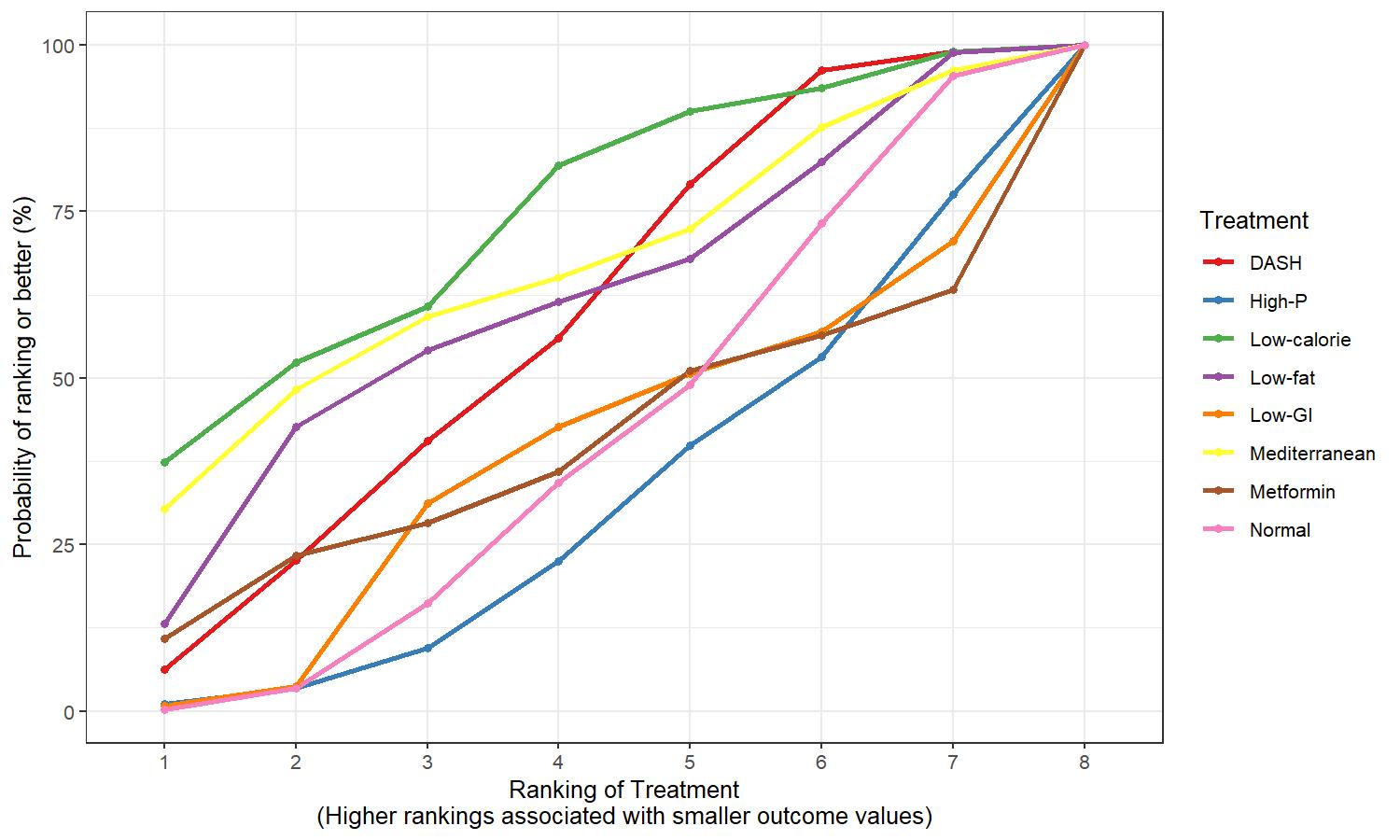


Table S3: League table containing comparisons regarding BMI. Values are given as MD (95% credible interval).

Low-GI, Low-Glycemic Index diet; High-P, High-Protein diet


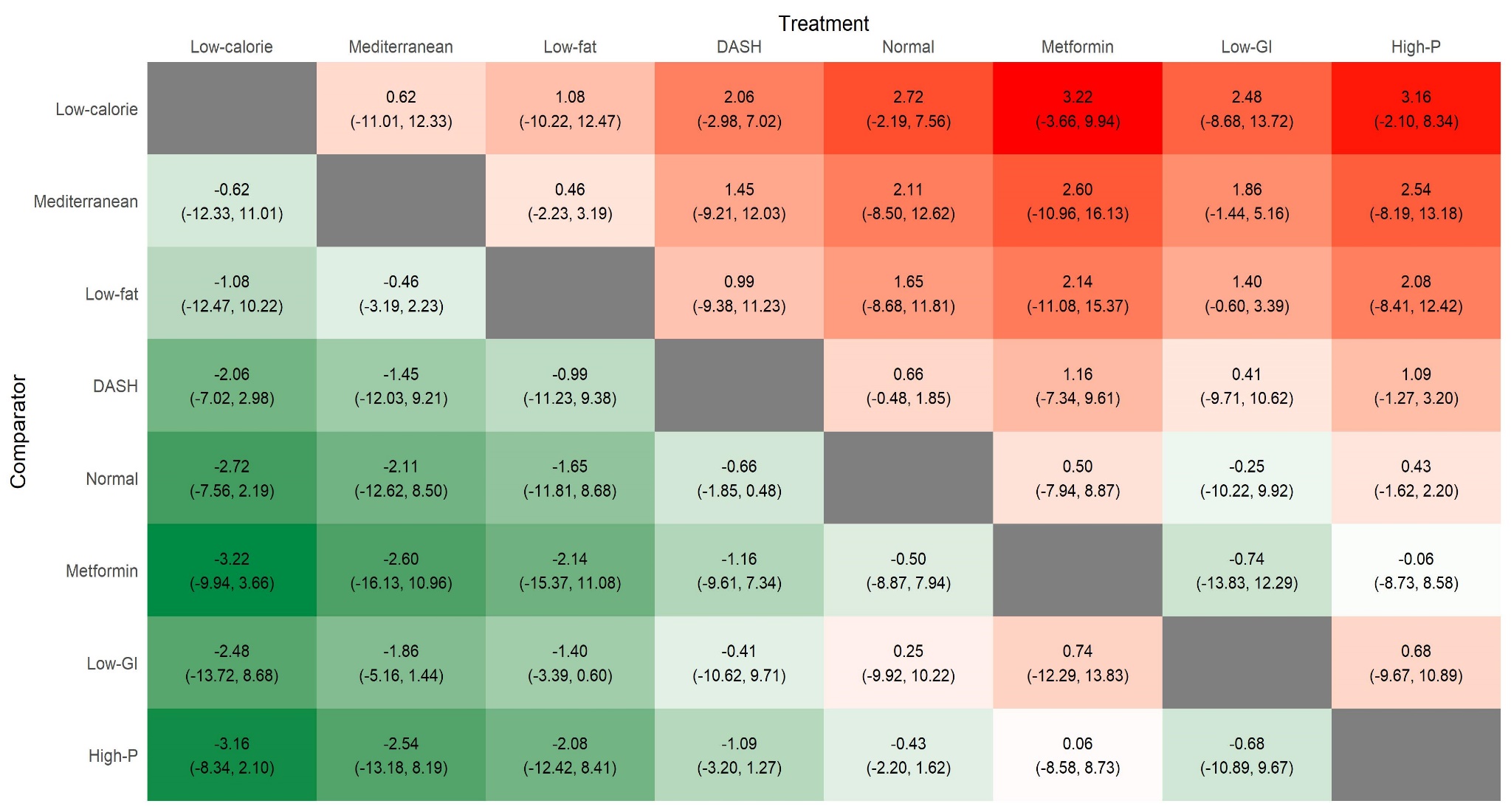


Figure S3: Rankogram representing surface under the cumulative ranking curve (SUCRA%) values of reducing weight. SUCRA values range from 0 to 100%. The higher the SUCRA value and the closer to 100%, the higher the likelihood that intervention is in the top rank or one of the top ranks.

*means: significant difference was observed compared to normal diet.

Low-calorie + M, Low-calorie diet plus metformin; DASH, Dietary approach stop hypertension; Low-GI, Low-Glycemic Index diet; High-P, High-protein diet


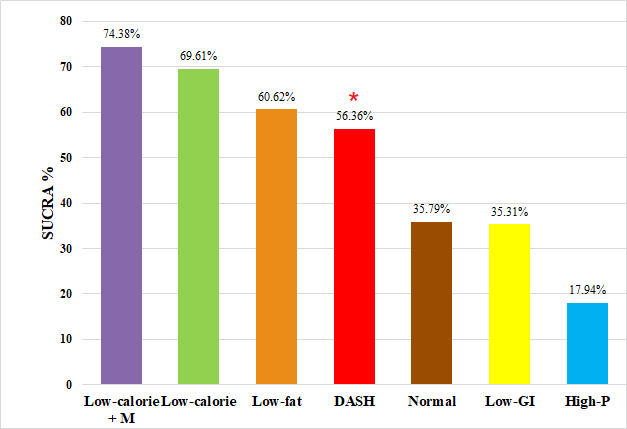


Figure S4: Surface under the cumulative ranking (SUCRA) curves of weight. SUCRA values range from 0 to 100%. The higher the SUCRA value, and the closer to 100%, the higher the likelihood that intervention is in the top rank or one of the top ranks.

Low-calorie + M, Low-calorie diet plus metformin; DASH, Dietary approach stop hypertension; Low-GI, Low-Glycemic Index diet; High-P, High-protein diet


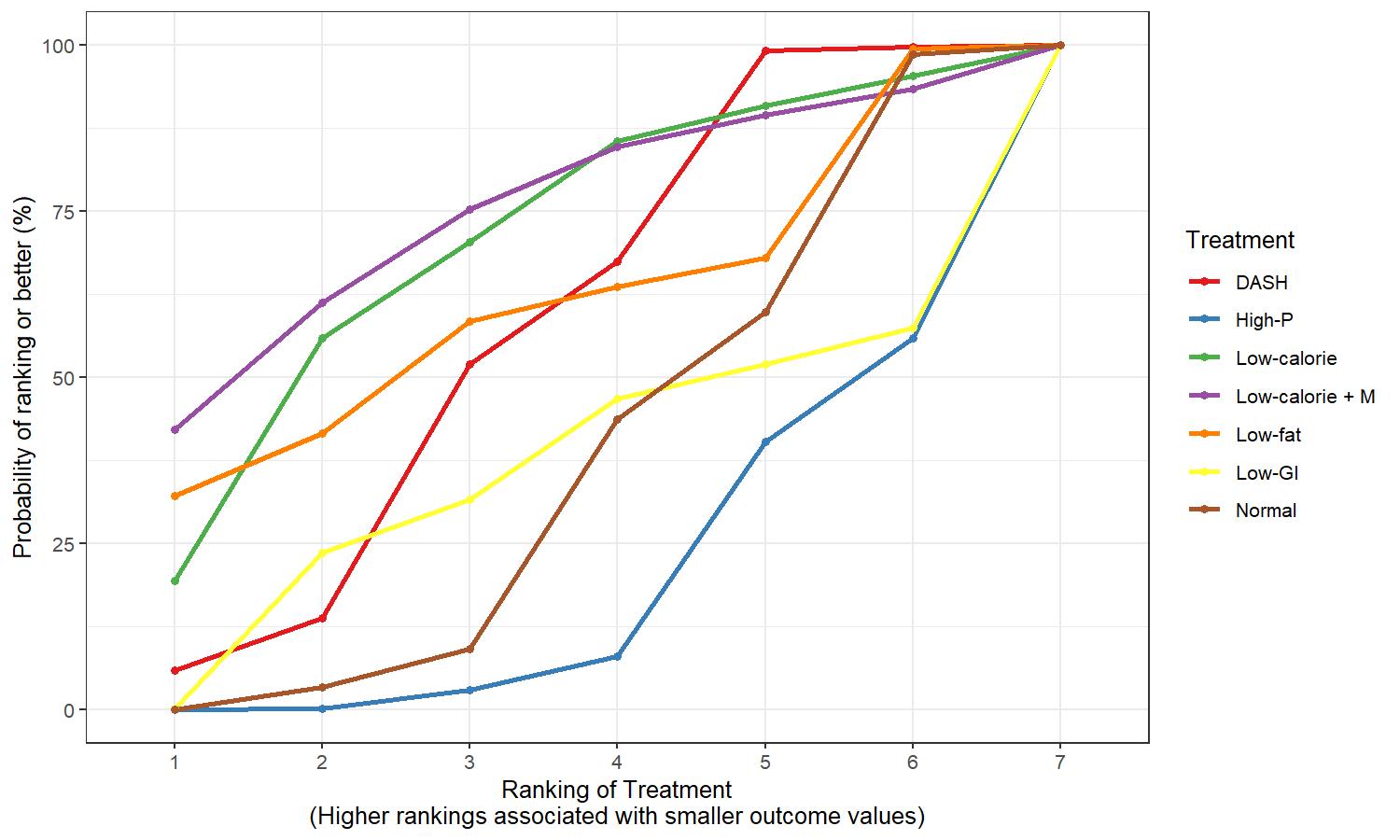


Table S4: League table containing comparisons regarding fasting blood glucose level. Values are given as MD (95% credible interval).

Low-calorie + M, Low-calorie diet plus metformin; DASH, Dietary approach stop hypertension; Low-GI, Low-Glycemic Index diet; High-P, High-protein diet


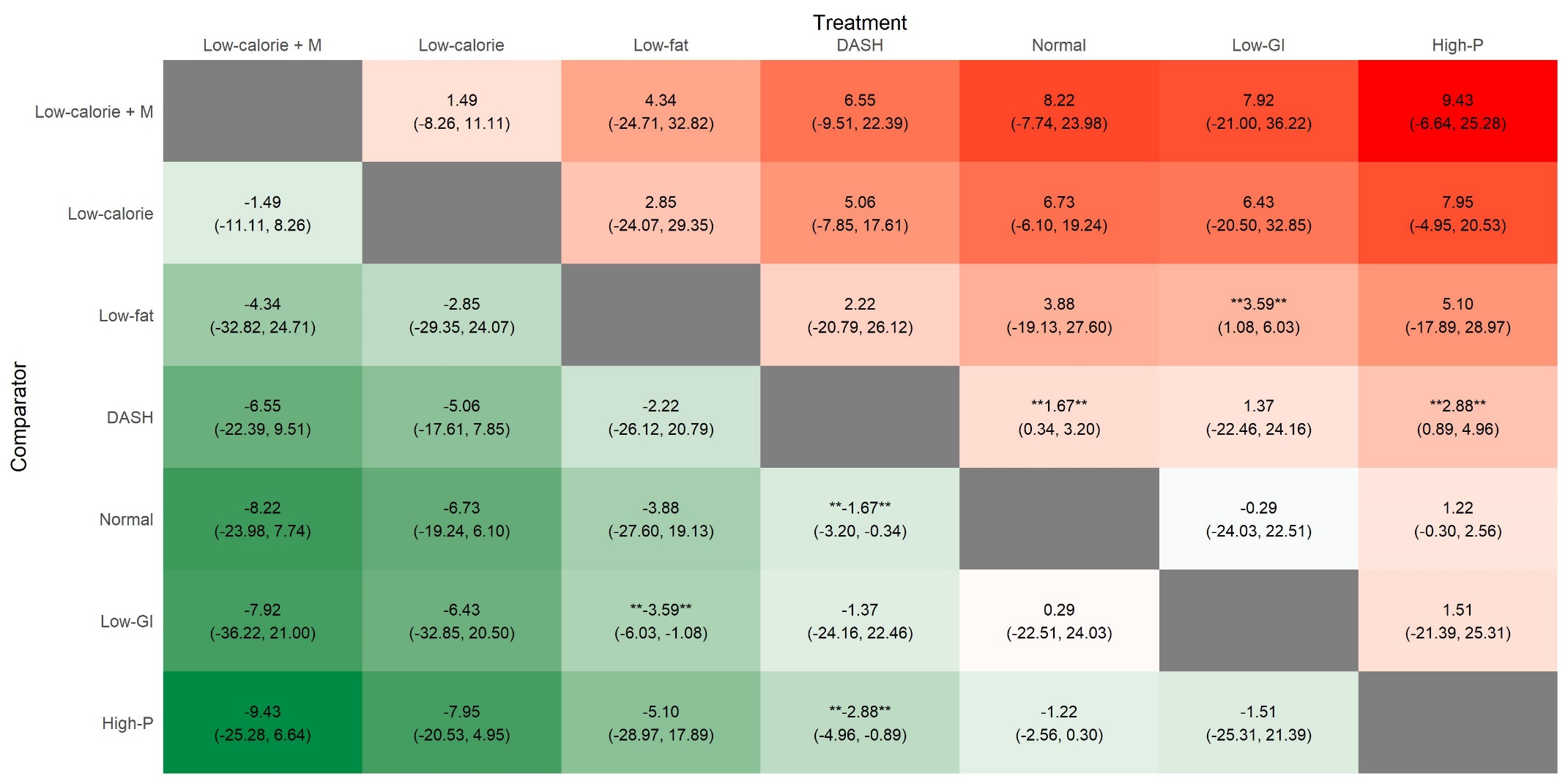


Figure S5: Rankogram representing surface under the cumulative ranking curve (SUCRA%) values of reducing HOMA-IR. SUCRA values range from 0 to 100%. The higher the SUCRA value and the closer to 100%, the higher the likelihood that intervention is in the top rank or one of the top ranks.

*means: significant difference was observed compared to normal diet.

DASH, Dietary approach stop hypertension; Low-calorie + M, Low-calorie diet plus metformin; Low-carb, Low- carbohydrate diet, Low-GI, Low-Glycemic Index diet


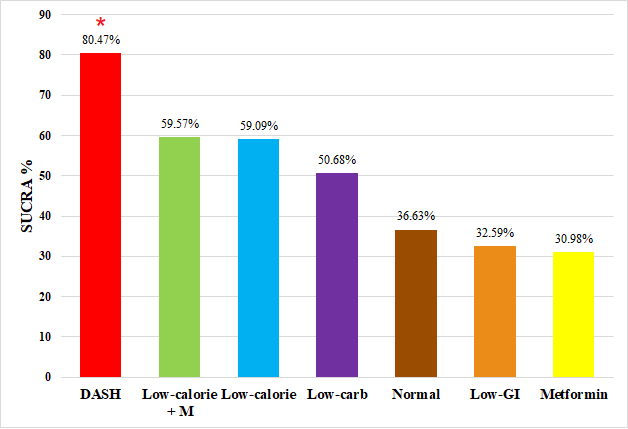


Figure S6: Surface under the cumulative ranking (SUCRA) curves of HOMA-IR. SUCRA values range from 0 to 100%. The higher the SUCRA value, and the closer to 100%, the higher the likelihood that intervention is in the top rank or one of the top ranks.

DASH, Dietary approach stop hypertension; Low-calorie + M, Low-calorie diet plus metformin; Low-carb, Low- carbohydrate diet, Low-GI, Low-Glycemic Index diet


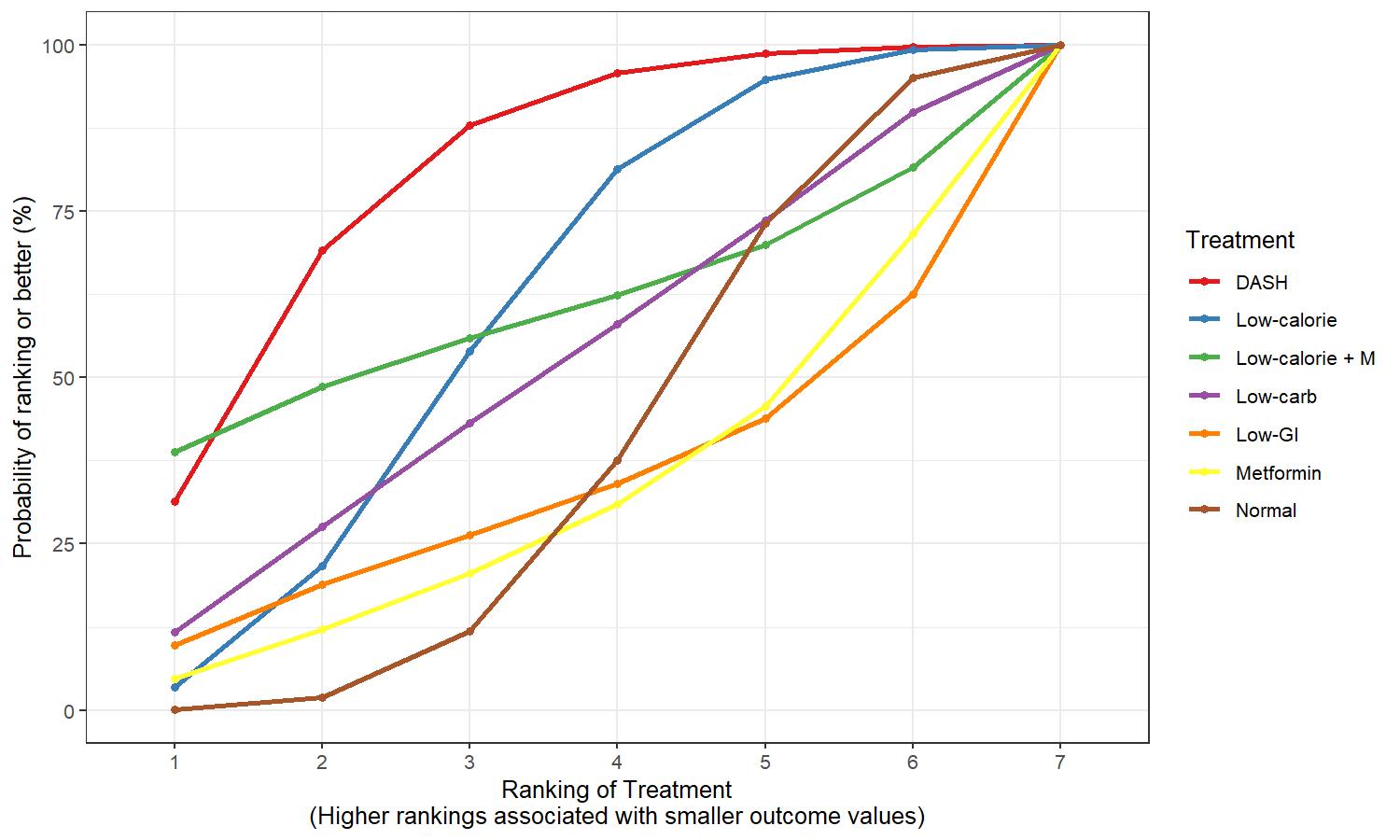


Table S5: League table containing comparisons regarding HOMA-IR. Values are given as MD (95% credible interval).

DASH, Dietary approach stop hypertension; Low-calorie + M, Low-calorie diet plus metformin; Low-carb, Low- carbohydrate diet, Low-GI, Low-Glycemic Index diet


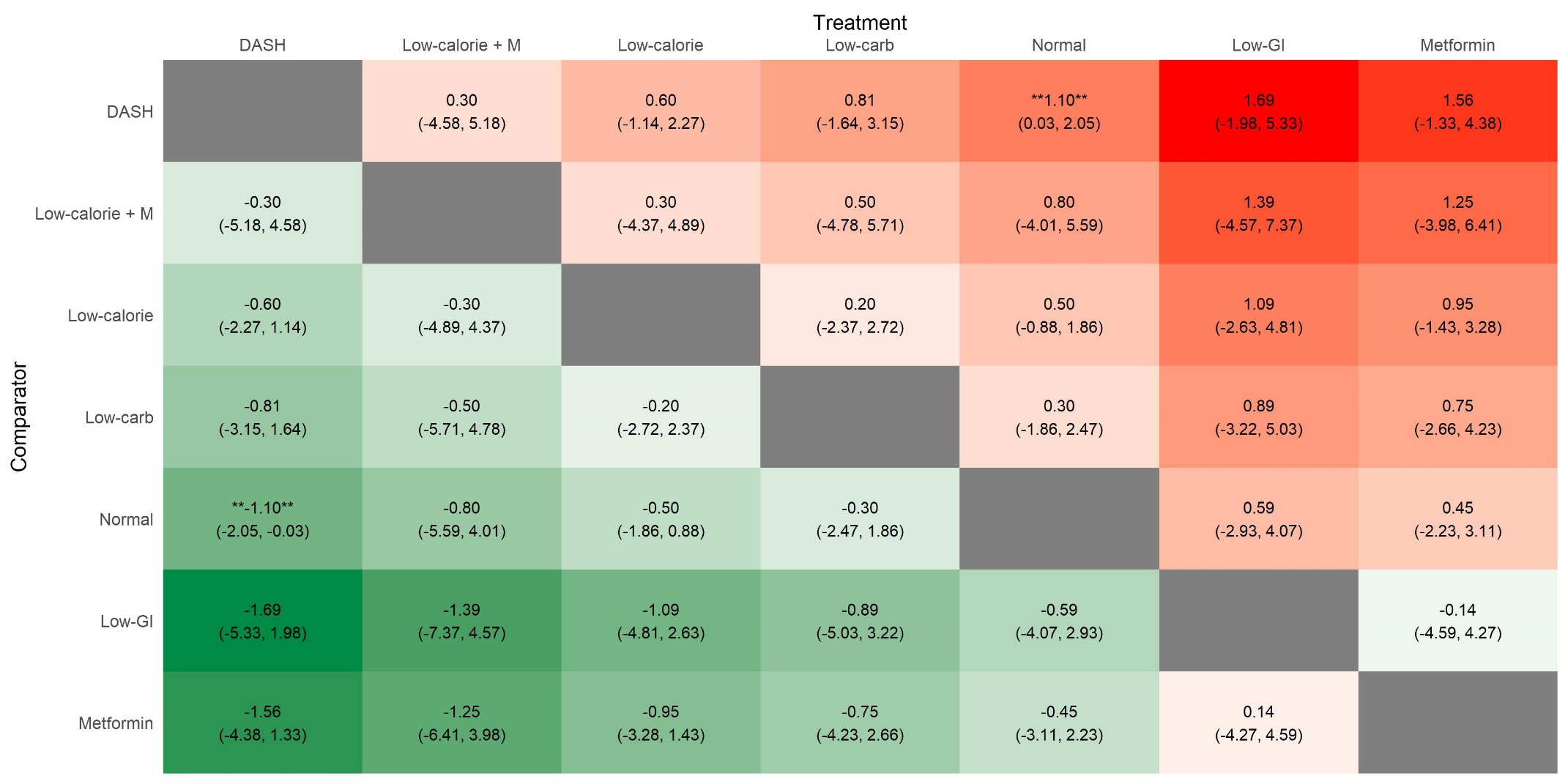


Figure S7: Rankogram representing surface under the cumulative ranking curve (SUCRA%) values of reducing fasting insulin level. SUCRA values range from 0 to 100%. The higher the SUCRA value and the closer to 100%, the higher the likelihood that intervention is in the top rank or one of the top ranks.

DASH, Dietary approach stop hypertension; Low-calorie + M, Low-calorie diet plus metformin; Low-carb, Low- carbohydrate diet; High-P, High-Protein diet; Low-GI, Low-Glycemic Index diet; Low-P, Low-Protein diet


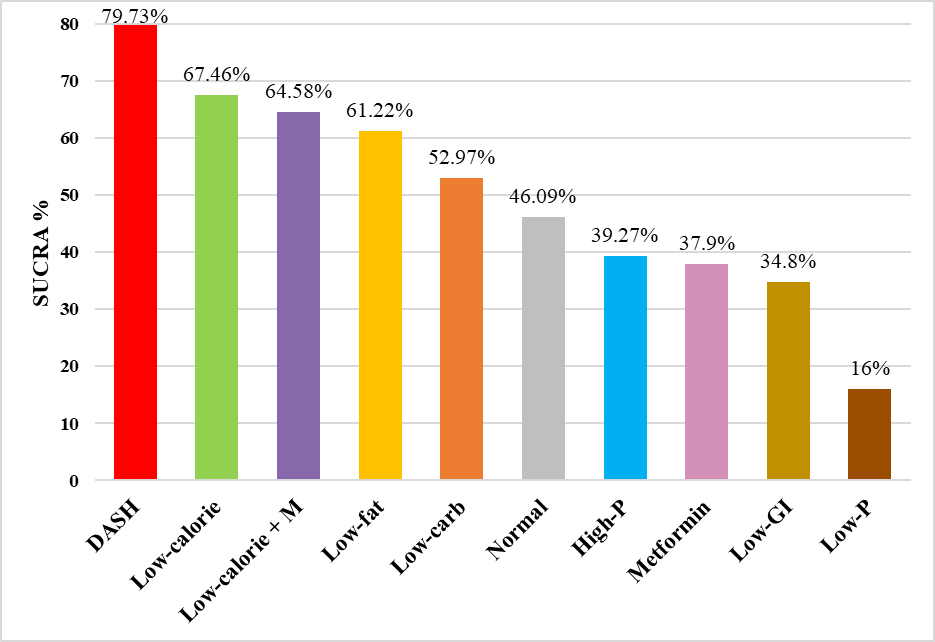


Figure S8: Surface under the cumulative ranking (SUCRA) curves of fasting insulin level. SUCRA values range from 0 to 100%. The higher the SUCRA value, and the closer to 100%, the higher the likelihood that intervention is in the top rank or one of the top ranks.

DASH, Dietary approach stop hypertension; Low-calorie + M, Low-calorie diet plus metformin; Low-carb, Low- carbohydrate diet; High-P, High-Protein diet; Low-GI, Low-Glycemic Index diet; Low-P, Low-Protein diet


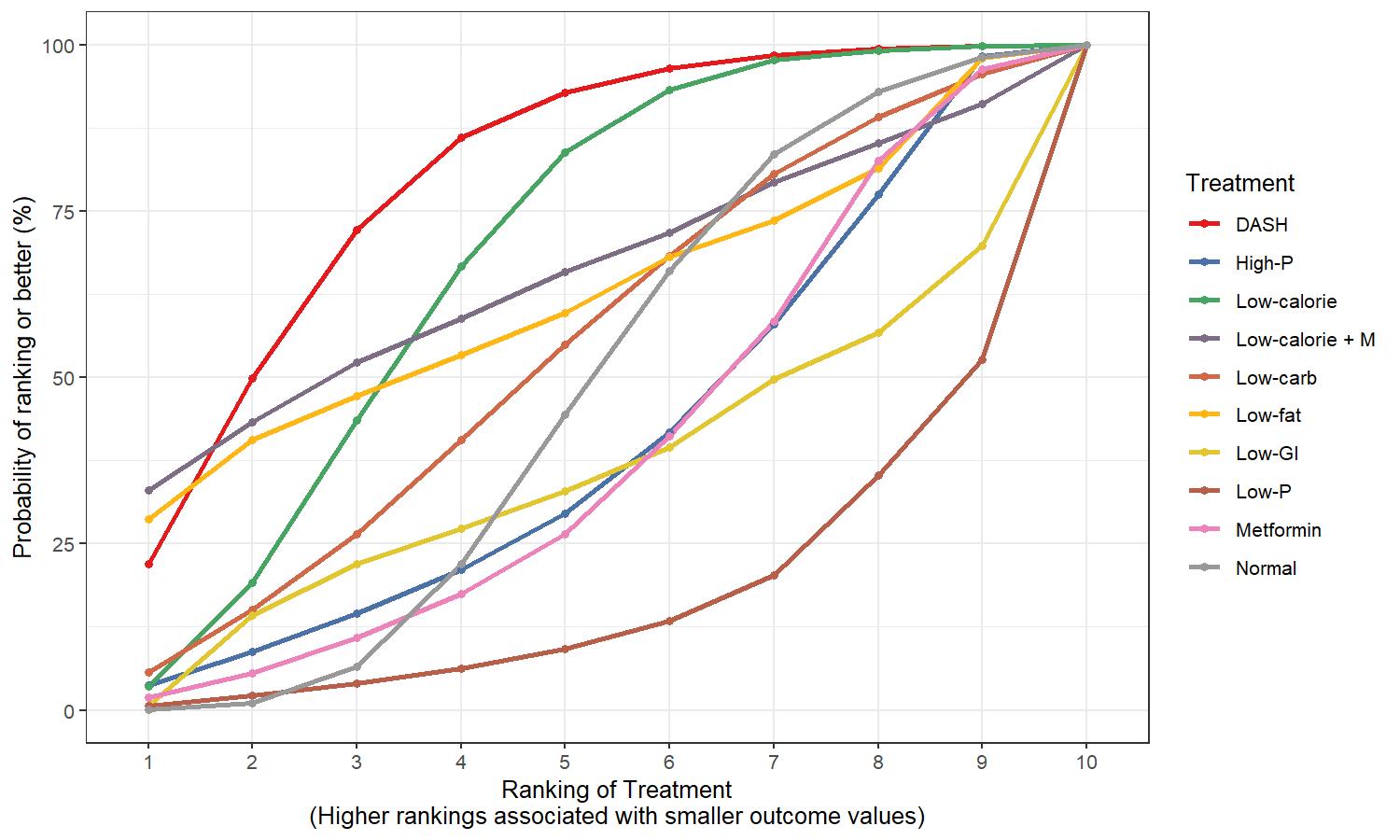


Table S6: League table containing comparisons regarding fasting insulin level. Values are given as MD (95% credible interval).DASH,

DASH, Dietary approach stop hypertension; Low-calorie + M, Low-calorie diet plus metformin; Low-carb, Low- carbohydrate diet; High-P, High-Protein diet; Low-GI, Low-Glycemic Index diet; Low-P, Low-Protein diet


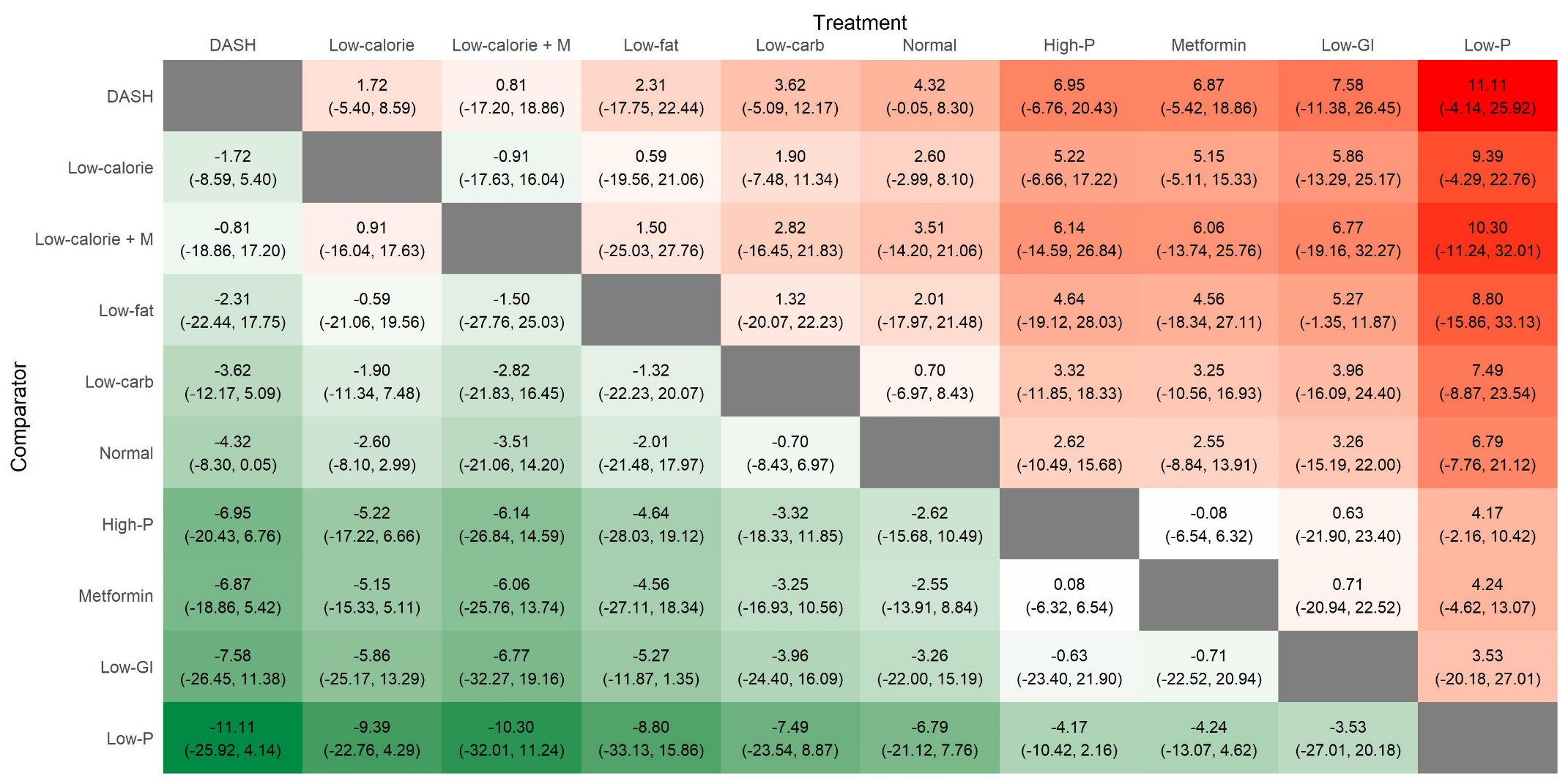


Figure S9: Rankogram representing surface under the cumulative ranking curve (SUCRA%) values of reducing fasting blood glucose level. postprandial blood glucose level. SUCRA values range from 0 to 100%. The higher the SUCRA value and the closer to 100%, the higher the likelihood that intervention is in the top rank or one of the top ranks.

DASH, Dietary approach stop hypertension; Low-carb, Low- carbohydrate; High-P, High-Protein diet; Low-GI, Low-Glycemic Index diet; Low-calorie + M, Low-calorie diet plus metformin; diet


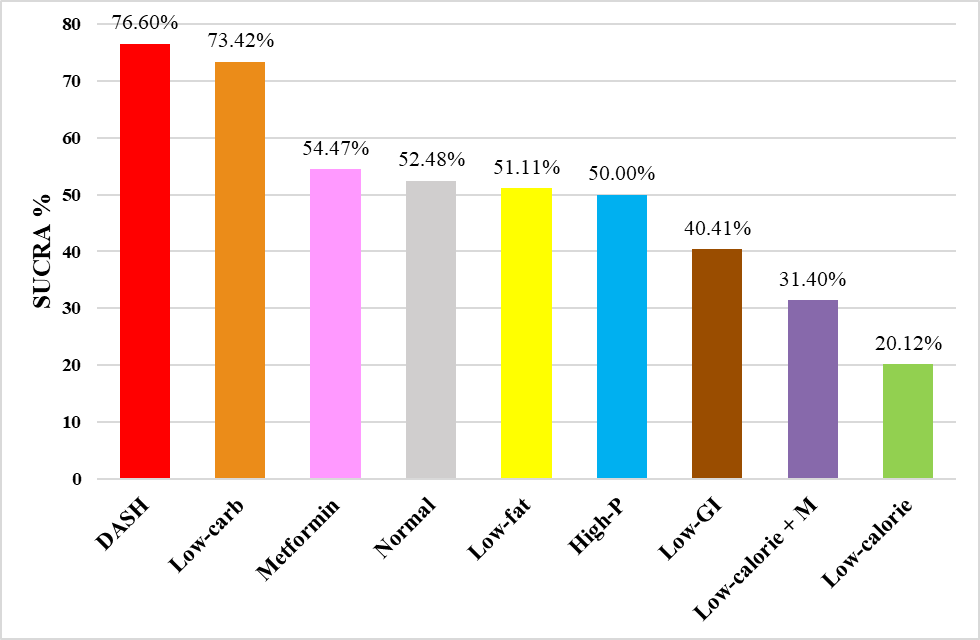


Figure S10: Surface under the cumulative ranking (SUCRA) curves of fasting blood glucose level. SUCRA values range from 0 to 100%. The higher the SUCRA value, and the closer to 100%, the higher the likelihood that intervention is in the top rank or one of the top ranks.

DASH, Dietary approach stop hypertension; Low-carb, Low- carbohydrate; High-P, High-Protein diet; Low-GI, Low-Glycemic Index diet; Low-calorie + M, Low-calorie diet plus metformin; diet


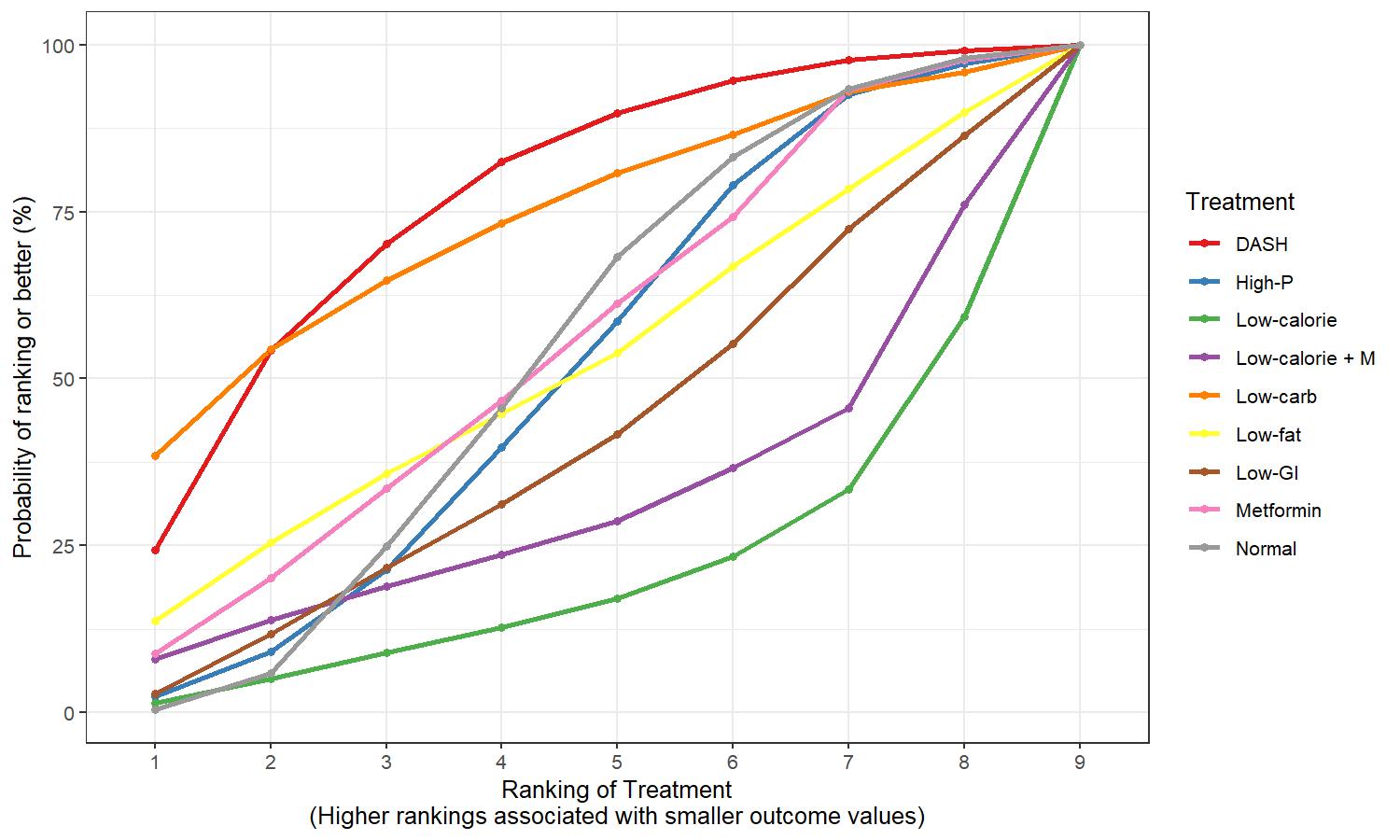


Table S7: League table containing comparisons regarding fasting blood glucose level. Values are given as MD (95% credible interval).
DASH, Dietary approach stop hypertension; Low-carb, Low- carbohydrate; High-P, High-Protein diet; Low-GI, Low-Glycemic Index diet; Low-calorie + M, Low-calorie diet plus metformin; diet


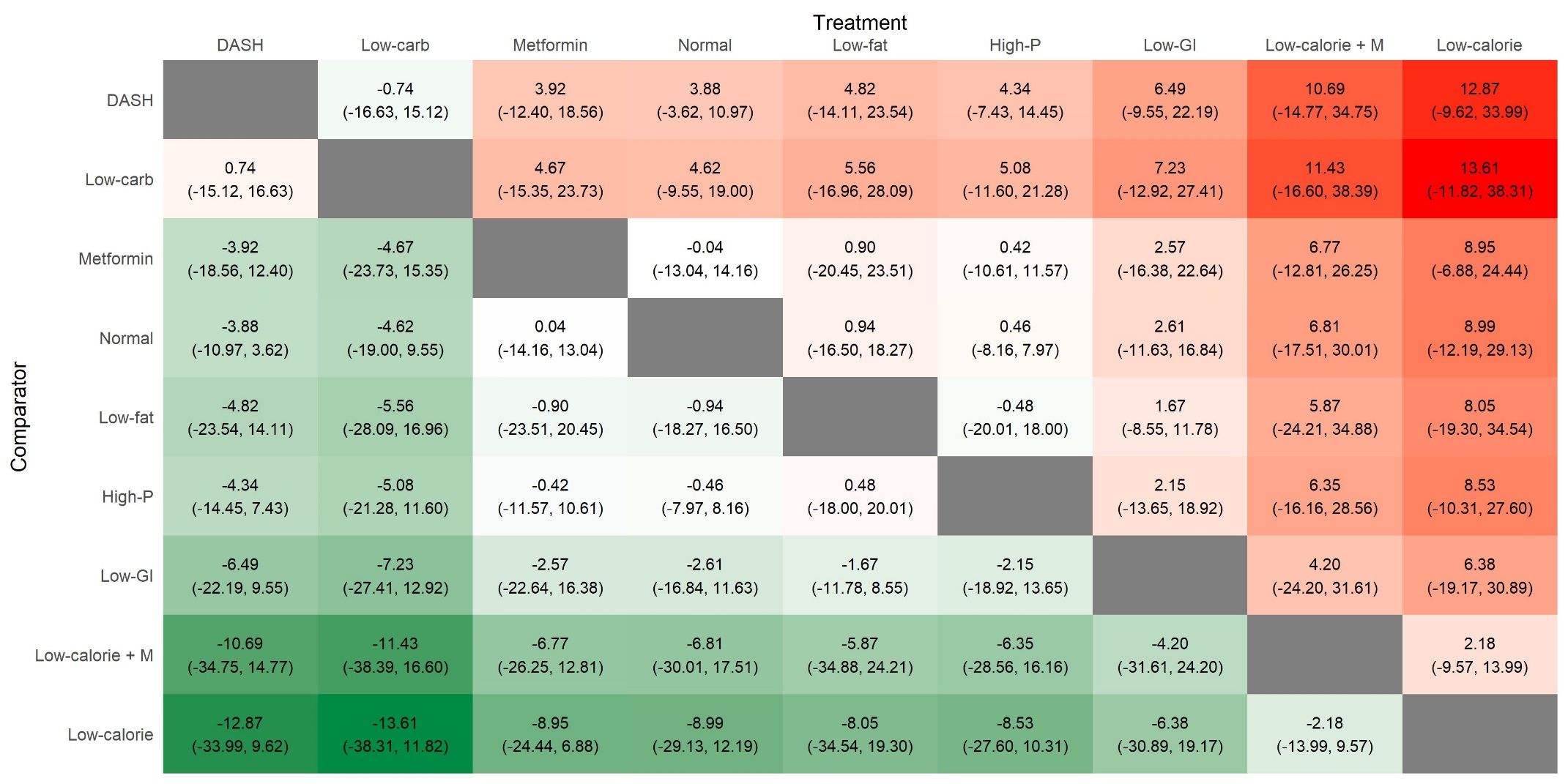


Figure S11: Rankogram representing surface under the cumulative ranking curve (SUCRA%) values of reducing total testosterone level. SUCRA values range from 0 to 100%. The higher the SUCRA value and the closer to 100%, the higher the likelihood that intervention is in the top rank or one of the top ranks.

Low-carb, Low- carbohydrate; DASH, Dietary approach stop hypertension; Low-GI, Low-Glycemic Index diet; High-P, High-Protein diet


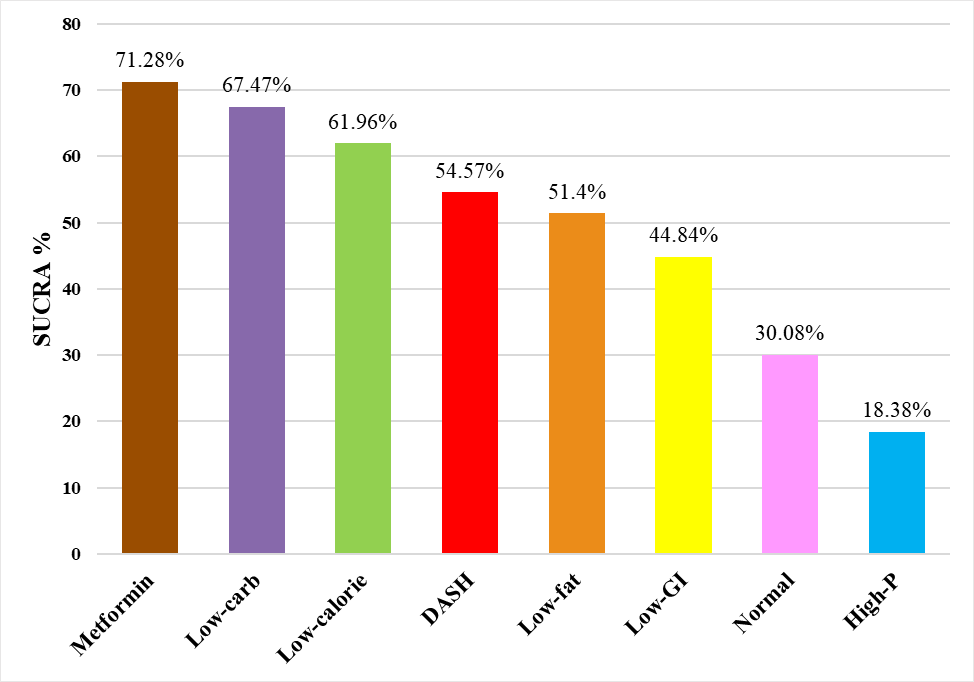


Figure S12: Surface under the cumulative ranking (SUCRA) curves of total testosterone level. SUCRA values range from 0 to 100%. The higher the SUCRA value, and the closer to 100%, the higher the likelihood that intervention is in the top rank or one of the top ranks.

Low-carb, Low- carbohydrate; DASH, Dietary approach stop hypertension; Low-GI, Low-Glycemic Index diet; High-P, High-Protein diet


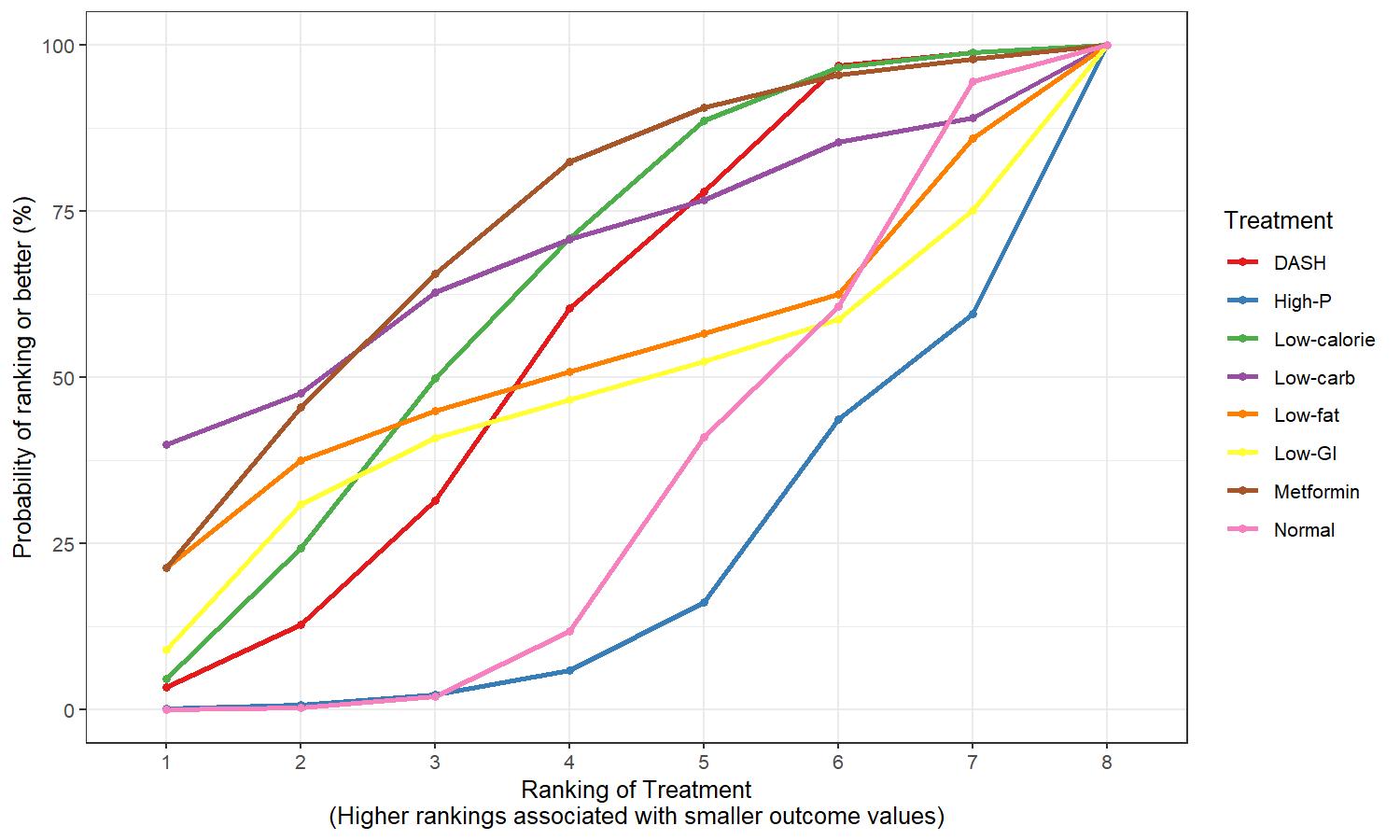


Table S8: League table containing comparisons regarding total testosterone level. Values are given as MD (95% credible interval).

Low-carb, Low- carbohydrate; DASH, Dietary approach stop hypertension; Low-GI, Low-Glycemic Index diet; High-P, High-Protein diet


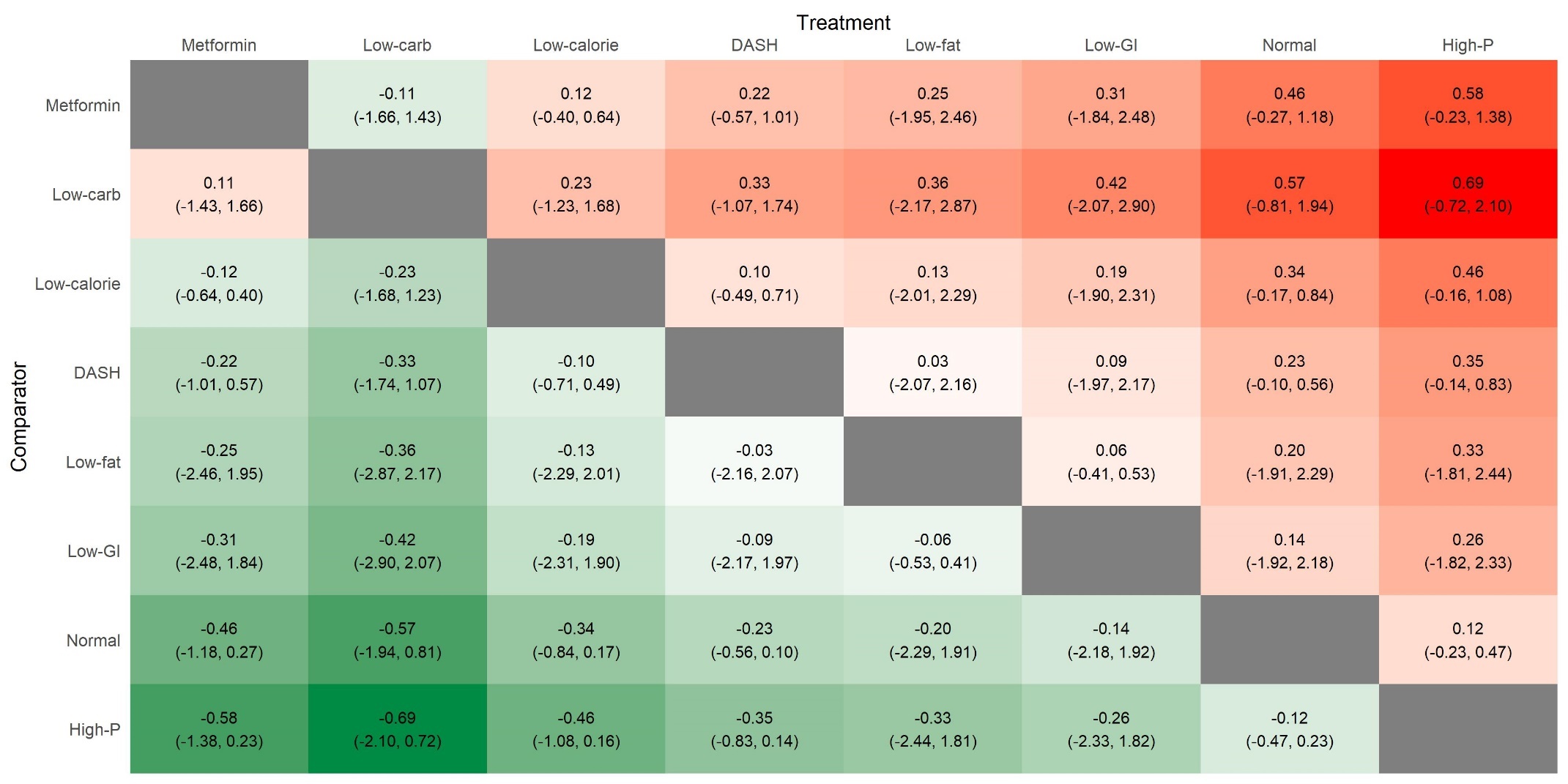


Figure S13: Rankogram representing surface under the cumulative ranking curve (SUCRA%) values of reducing LDL level. SUCRA values range from 0 to 100%. The higher the SUCRA value and the closer to 100%, the higher the likelihood that intervention is in the top rank or one of the top ranks.

High-P, High-Protein diet; DASH, Dietary approach stop hypertension; Low-carb, Low- carbohydrate


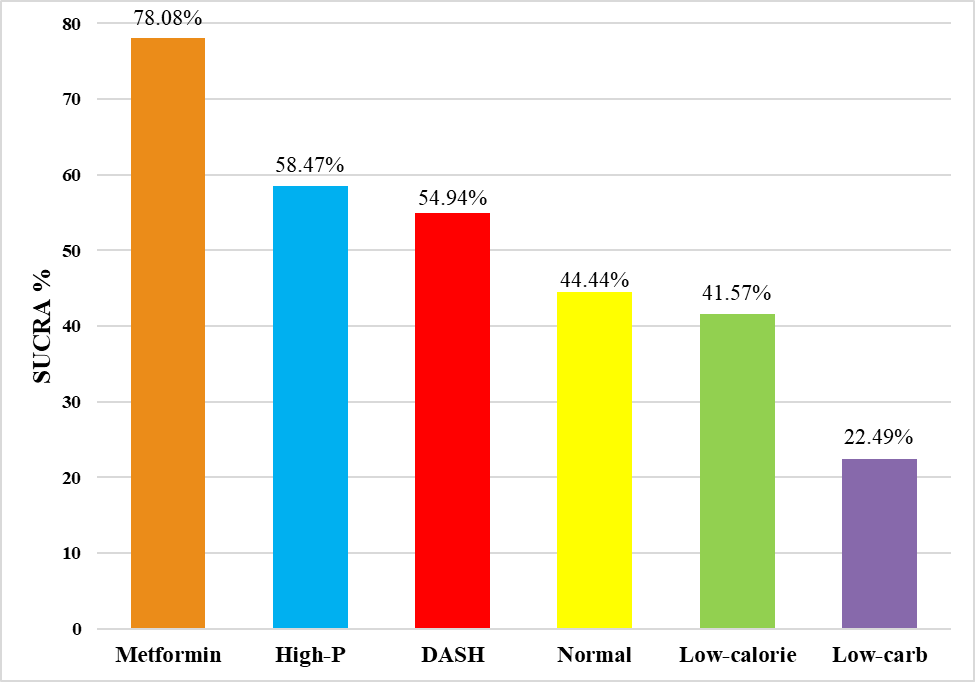


Figure S14: Surface under the cumulative ranking (SUCRA) curves of LDL level. SUCRA values range from 0 to 100%. The higher the SUCRA value, and the closer to 100%, the higher the likelihood that intervention is in the top rank or one of the top ranks.

High-P, High-Protein diet; DASH, Dietary approach stop hypertension; Low-carb, Low- carbohydrate


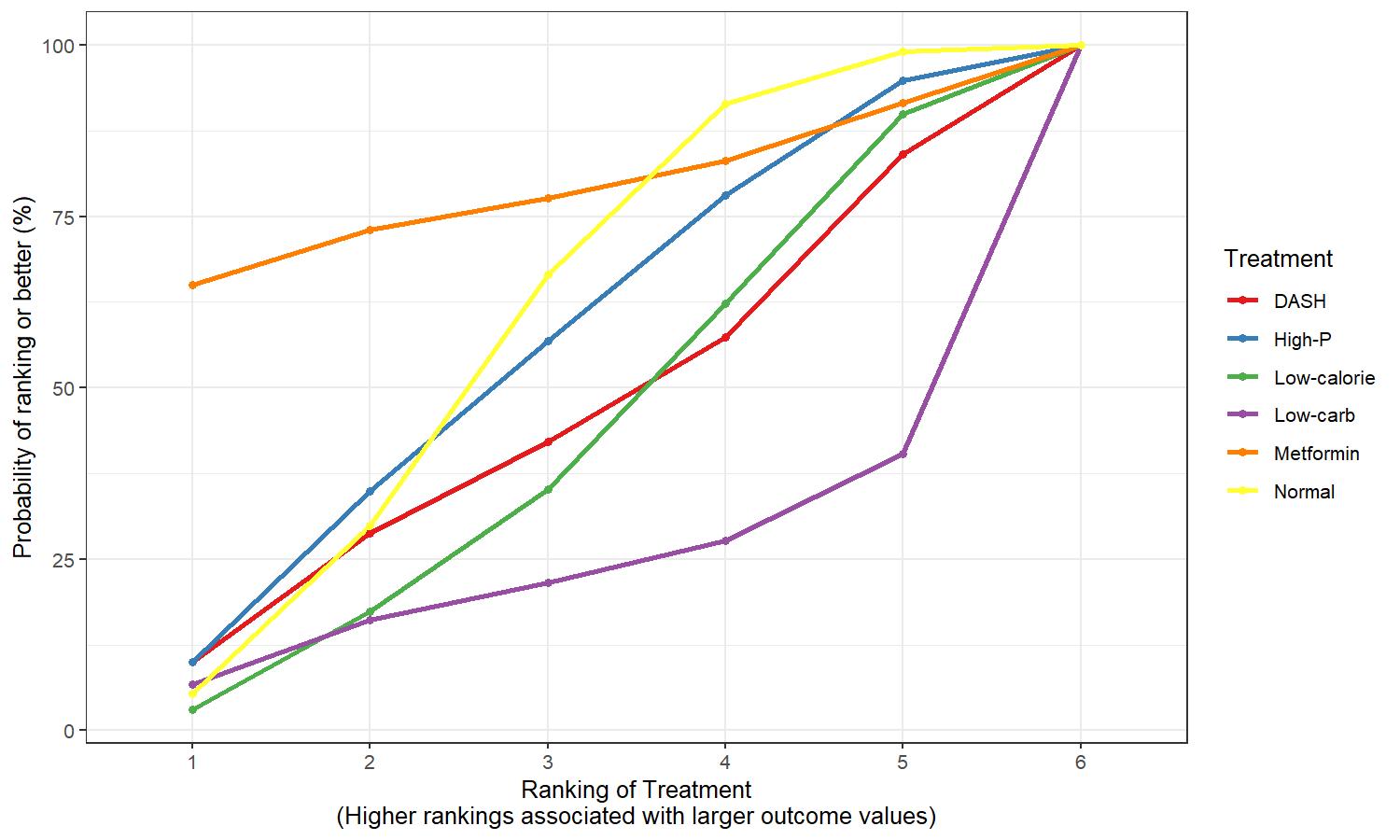


Table S9: League table containing comparisons regarding LDL level. Values are given as MD (95% credible interval).

High-P, High-Protein diet; DASH, Dietary approach stop hypertension; Low-carb, Low- carbohydrate


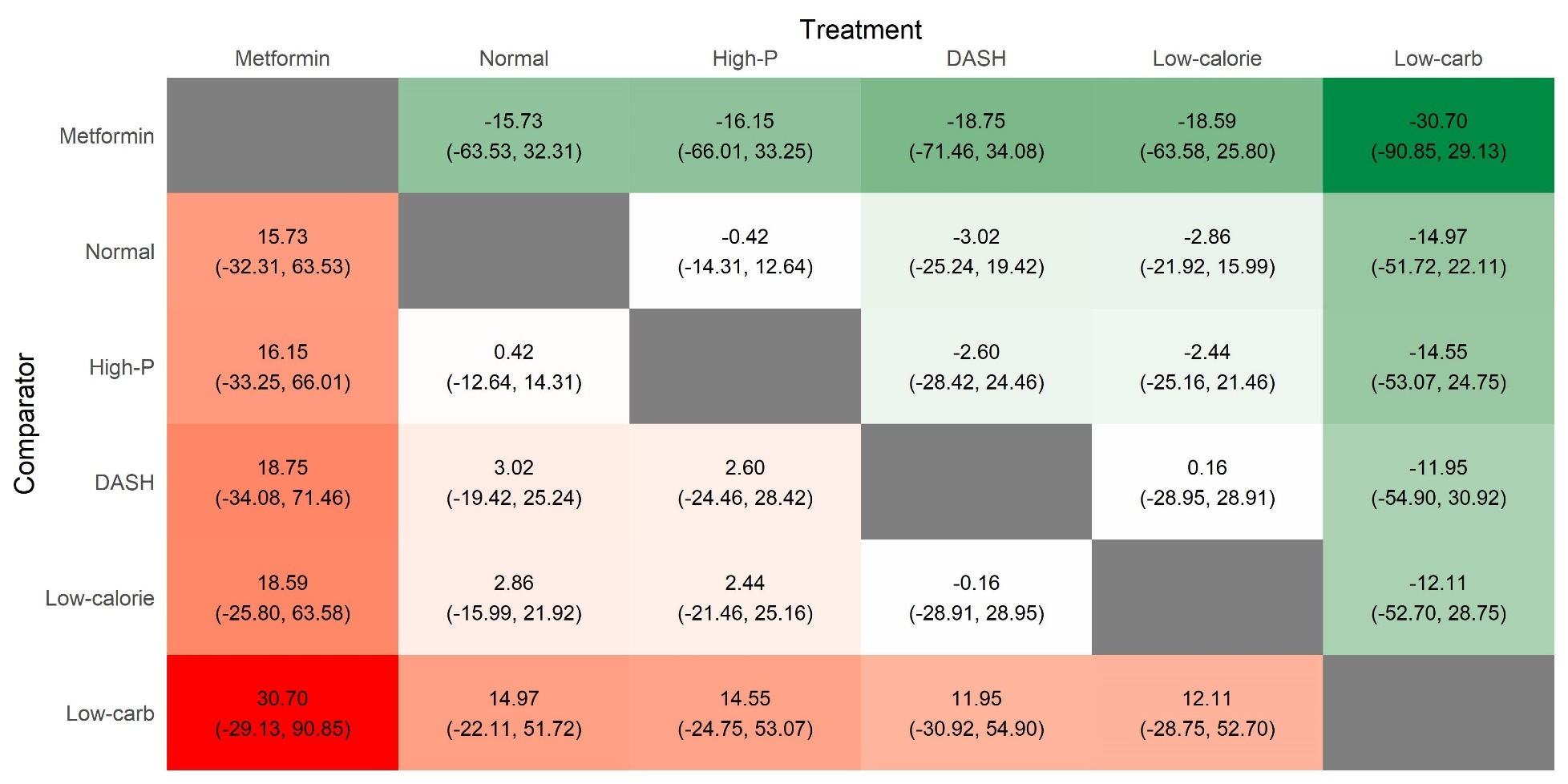


Figure S15: Rankogram representing surface under the cumulative ranking curve (SUCRA%) values of HDL level. SUCRA values range from 0 to 100%. The higher the SUCRA value and the closer to 100%, the higher the likelihood that intervention is in the top rank or one of the top ranks.

DASH, Dietary approach stop hypertension; Low-carb, Low- carbohydrate; High-P, High-Protein diet


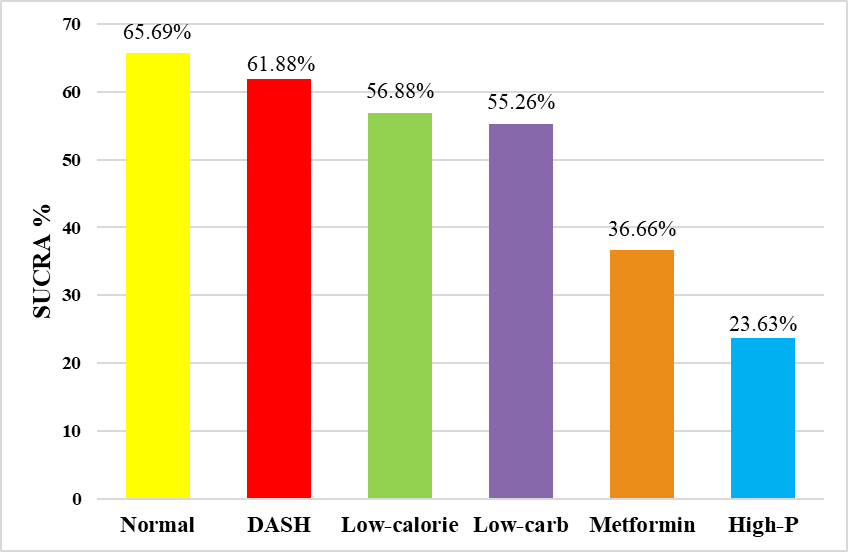


Figure S16: Surface under the cumulative ranking (SUCRA) curves of HDL level. SUCRA values range from 0 to 100%. The higher the SUCRA value, and the closer to 100%, the higher the likelihood that intervention is in the top rank or one of the top ranks.

DASH, Dietary approach stop hypertension; Low-carb, Low- carbohydrate; High-P, High-Protein diet


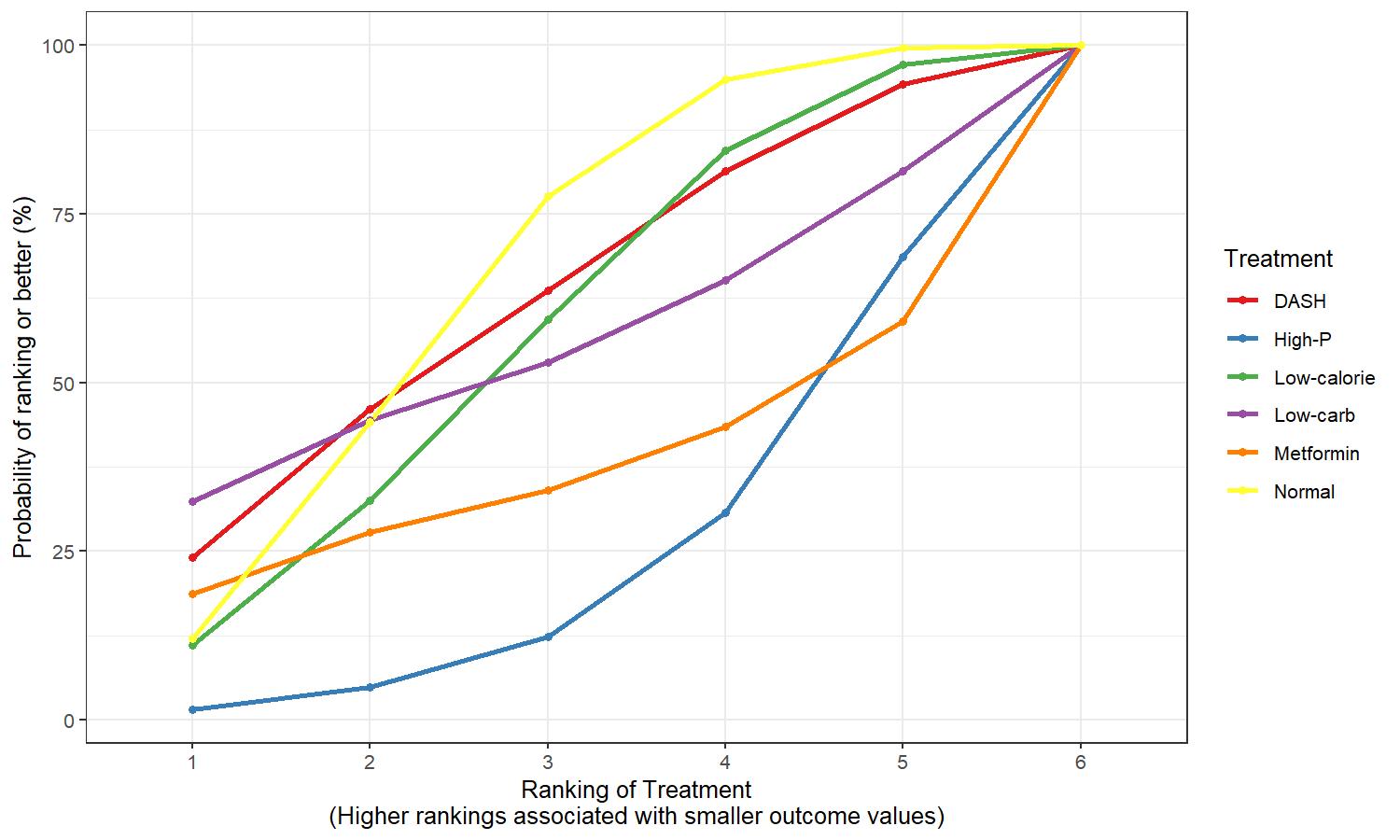


Table S10: League table containing comparisons regarding HDL level. Values are given as MD (95% credible interval).

DASH, Dietary approach stop hypertension; Low-carb, Low- carbohydrate; High-P, High-Protein diet


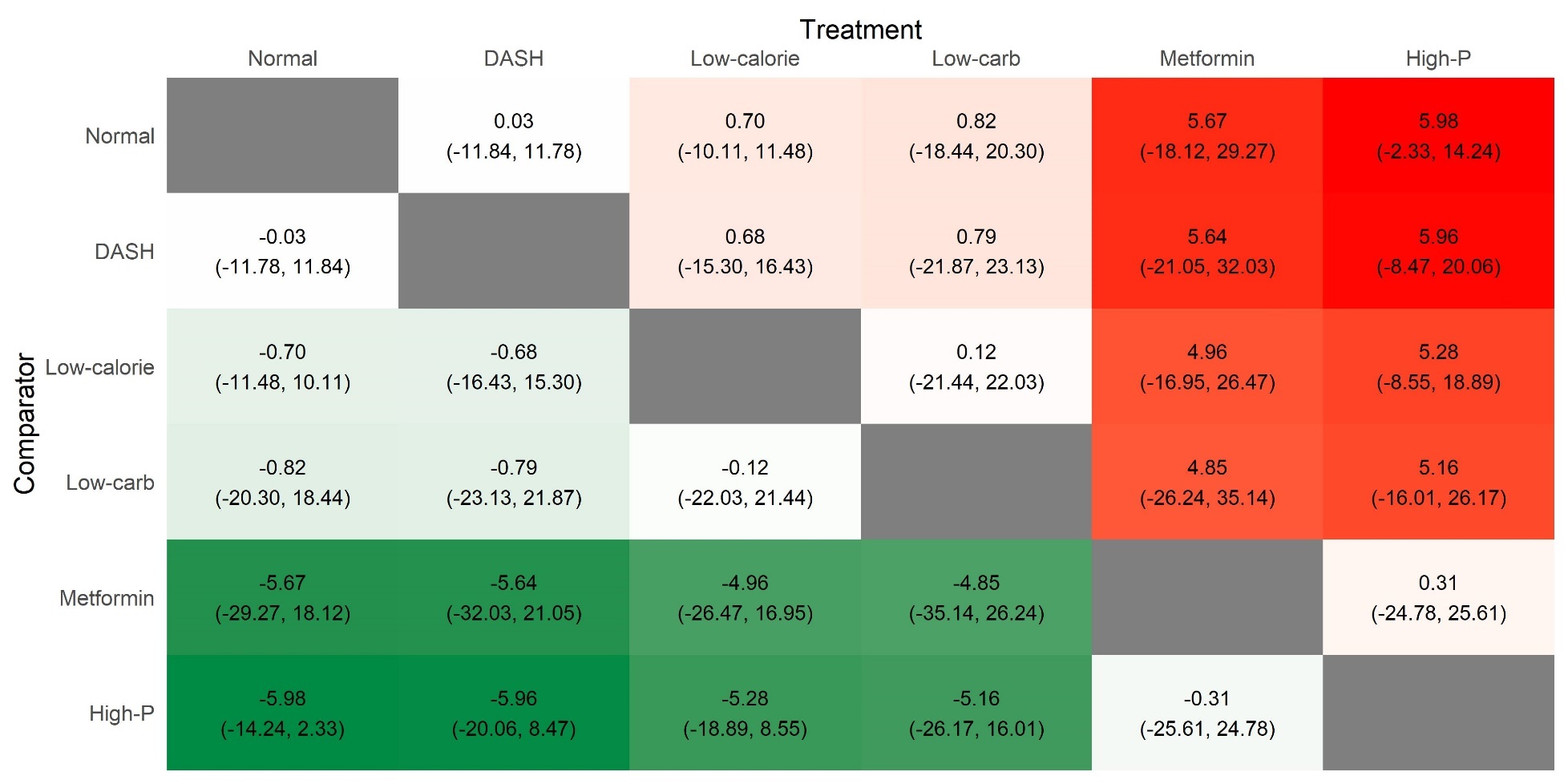


Figure S17: Rankogram representing surface under the cumulative ranking curve (SUCRA%) values of reducing triglyceride level. SUCRA values range from 0 to 100%. The higher the SUCRA value and the closer to 100%, the higher the likelihood that intervention is in the top rank or one of the top ranks.

DASH, Dietary approach stop hypertension; Low-GI, Low-Glycemic Index diet; High-P, High-Protein diet; Low-carb, Low- carbohydrate


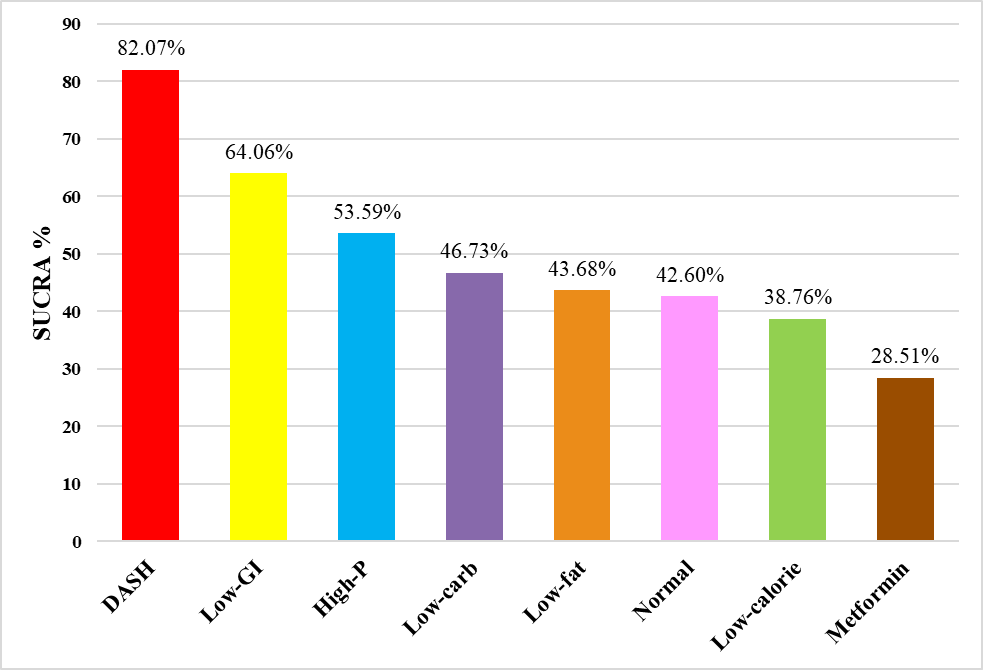


Figure S18: Surface under the cumulative ranking (SUCRA) curves of triglyceride level. SUCRA values range from 0 to 100%. The higher the SUCRA value, and the closer to 100%, the higher the likelihood that intervention is in the top rank or one of the top ranks.

DASH, Dietary approach stop hypertension; Low-GI, Low-Glycemic Index diet; High-P, High-Protein diet; Low-carb, Low- carbohydrate


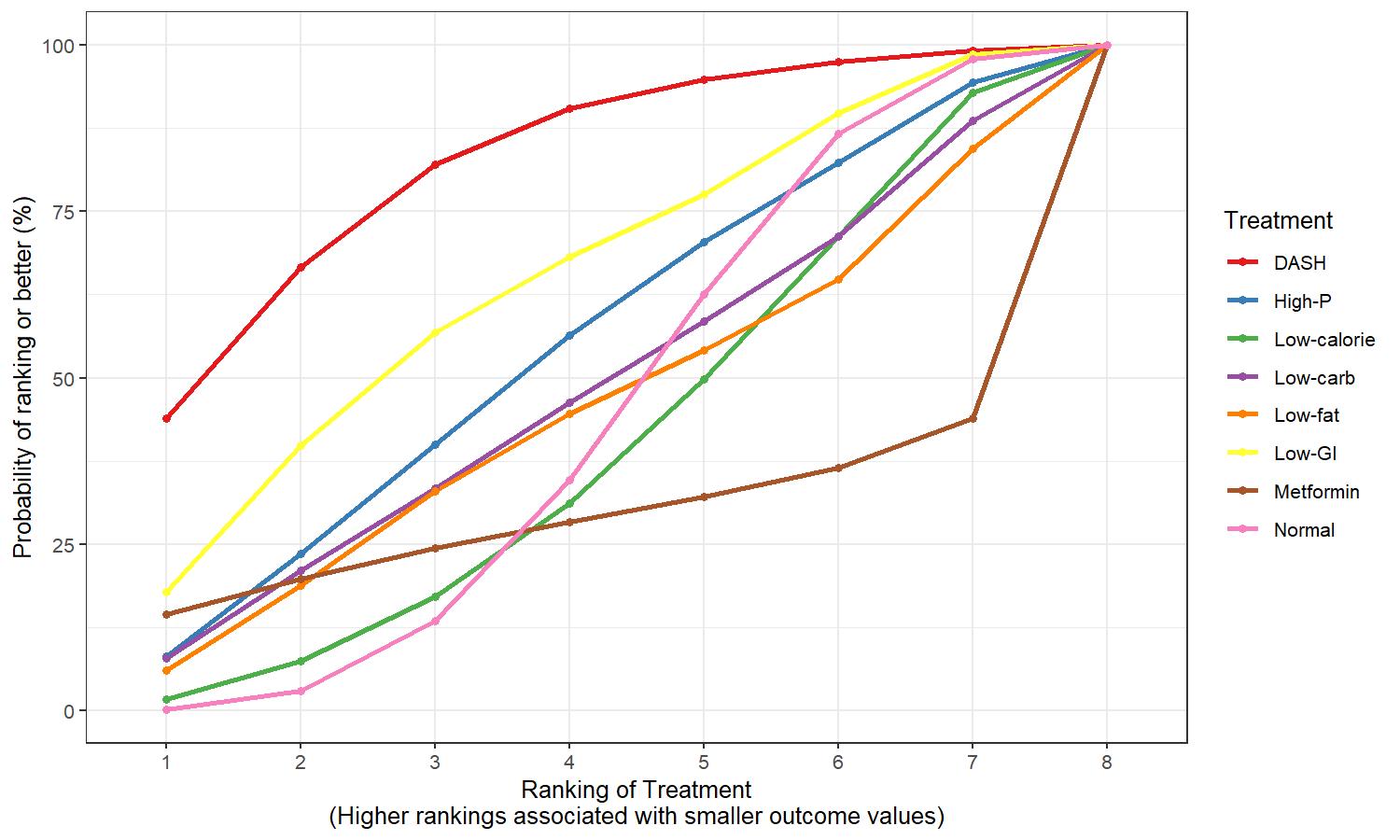


Table S11: League table containing comparisons regarding triglyceride level. Values are given as MD (95% credible interval).

DASH, Dietary approach stop hypertension; Low-GI, Low-Glycemic Index diet; High-P, High-Protein diet; Low-carb, Low- carbohydrate


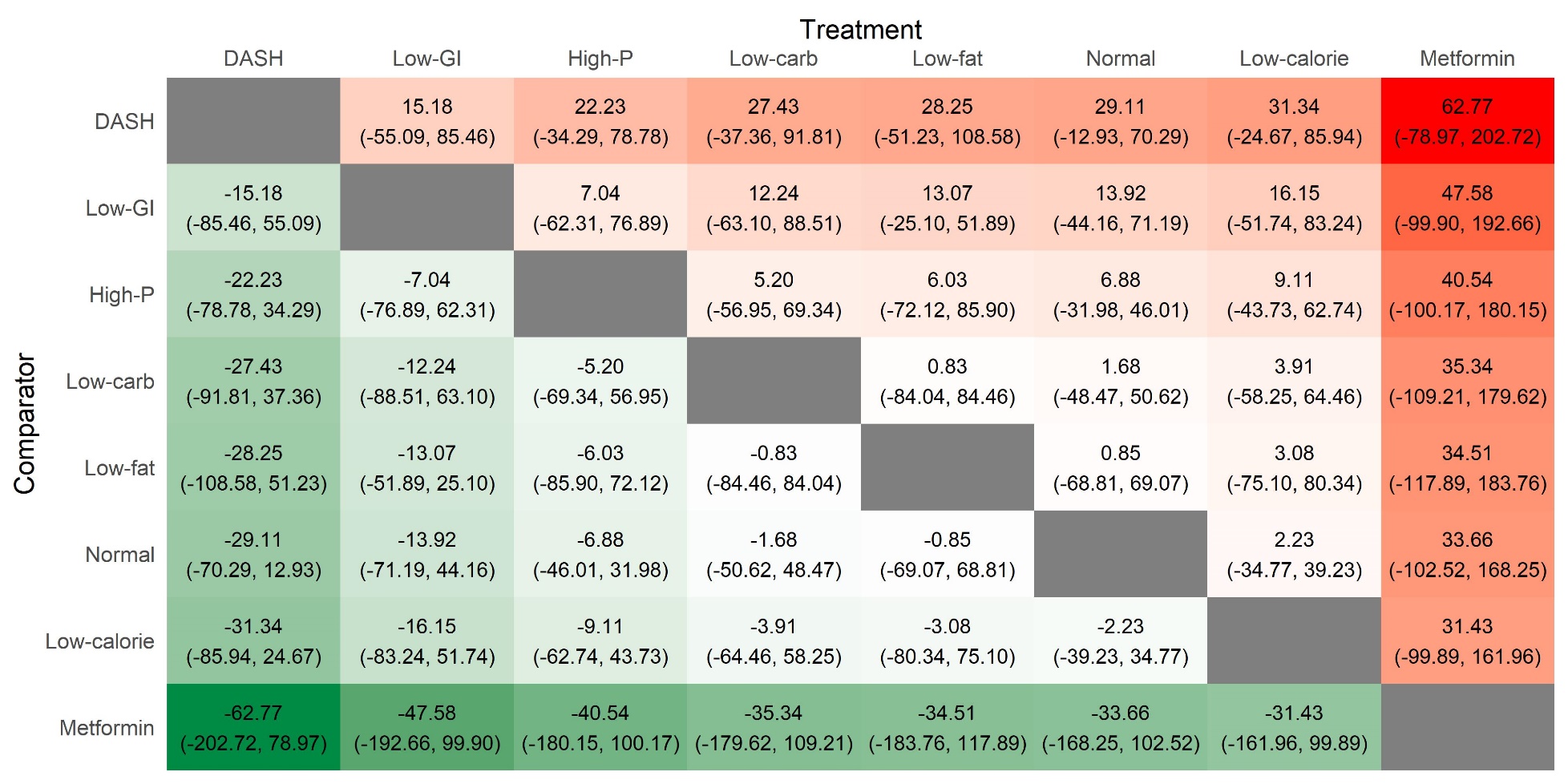


Figure S19: Rankogram representing surface under the cumulative ranking curve (SUCRA%) values of reducing cholesterol level. SUCRA values range from 0 to 100%. The higher the SUCRA value and the closer to 100%, the higher the likelihood that intervention is in the top rank or one of the top ranks.

Low-carb, Low- carbohydrate; DASH, Dietary approach stop hypertension; Low-GI, Low-Glycemic Index diet; High-P, High-Protein diet


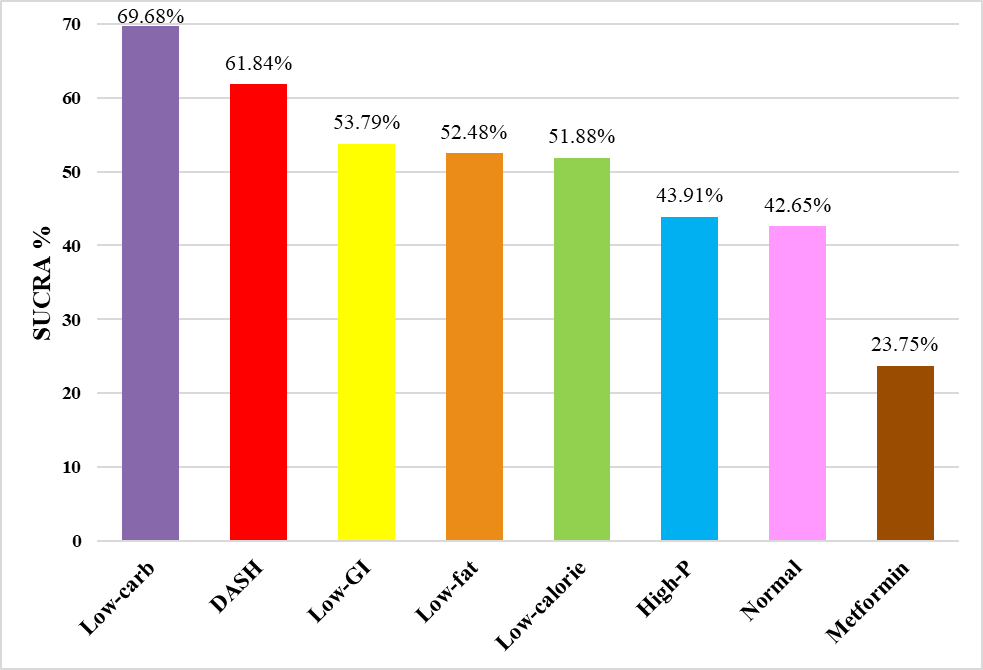


Figure S20: Surface under the cumulative ranking (SUCRA) curves of cholesterol level. SUCRA values range from 0 to 100%. The higher the SUCRA value, and the closer to 100%, the higher the likelihood that intervention is in the top rank or one of the top ranks.

Low-carb, Low- carbohydrate; DASH, Dietary approach stop hypertension; Low-GI, Low-Glycemic Index diet; High-P, High-Protein diet


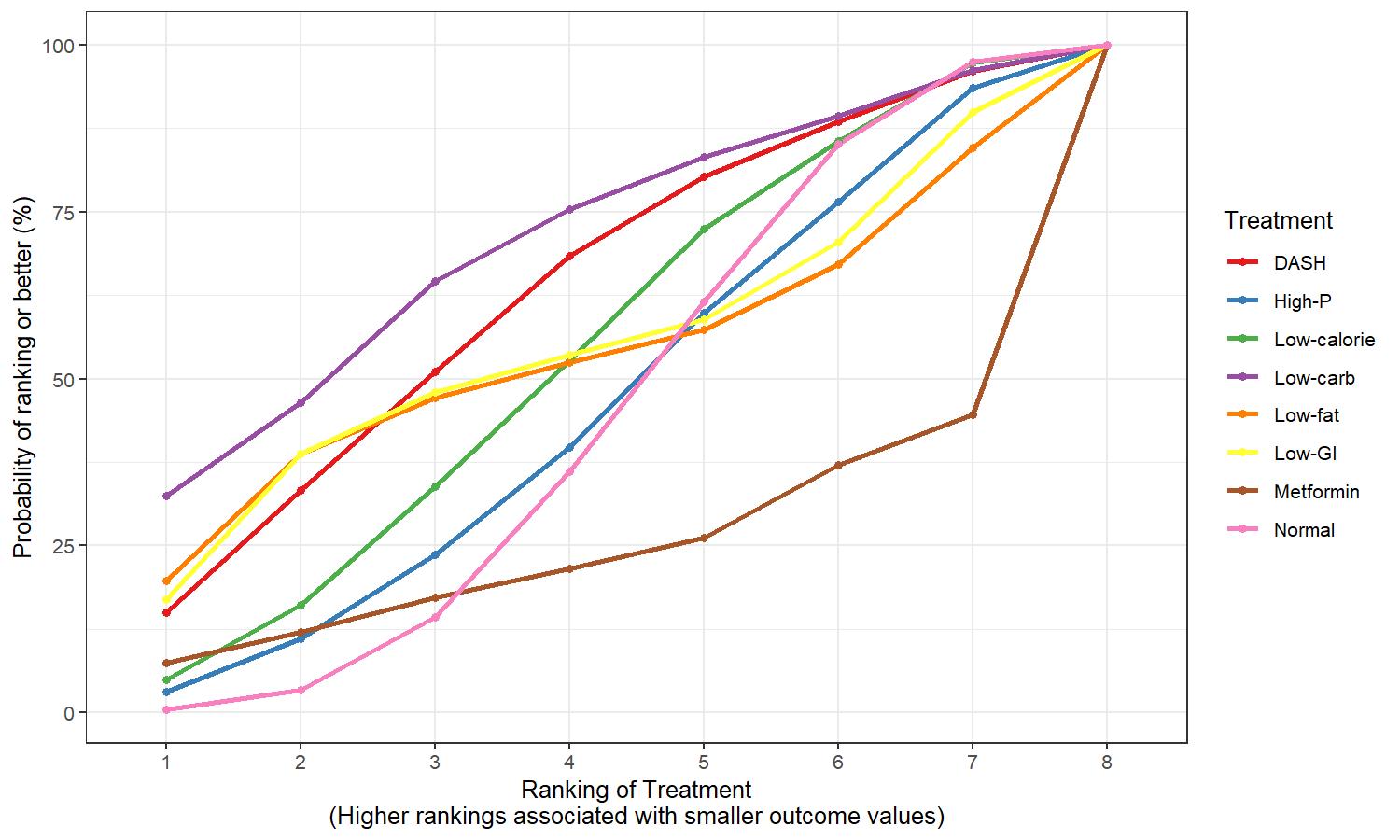


Table S12: League table containing comparisons regarding cholesterol level. Values are given as MD (95% credible interval).

Low-carb, Low- carbohydrate; DASH, Dietary approach stop hypertension; Low-GI, Low-Glycemic Index diet; High-P, High-Protein diet


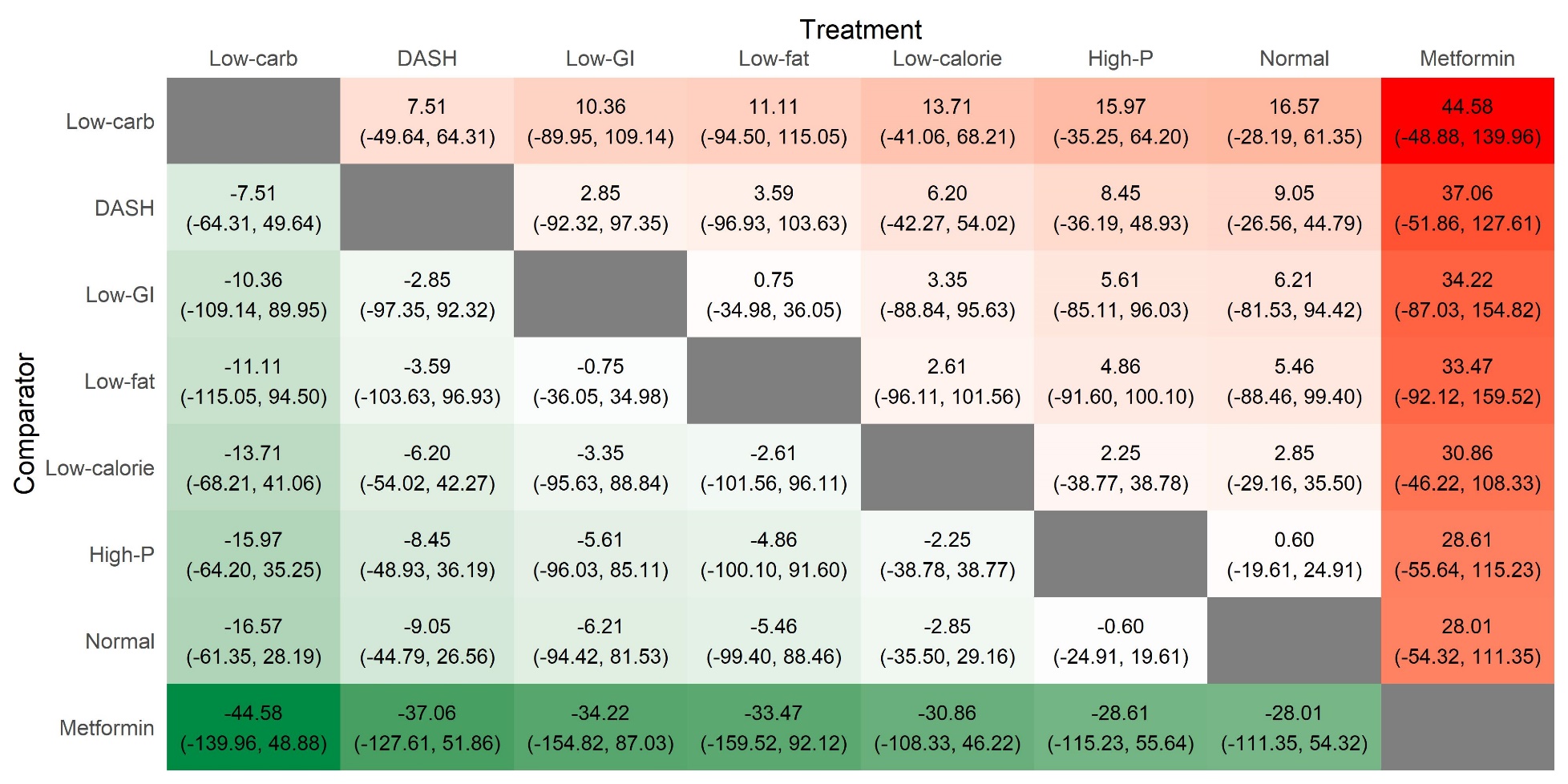


# Figure S21: Risk of bias assessment at study level regarding BMI.

# Figure S22: Risk of bias assessment at domain level regarding BMI.


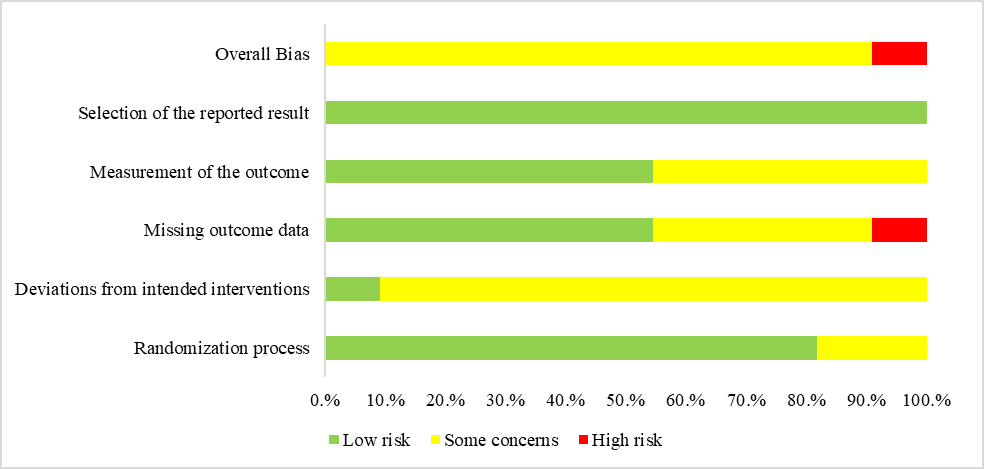


# Figure S23: Risk of bias assessment at study level regarding weight.

# Figure S24: Risk of bias assessment at domain level regarding weight.


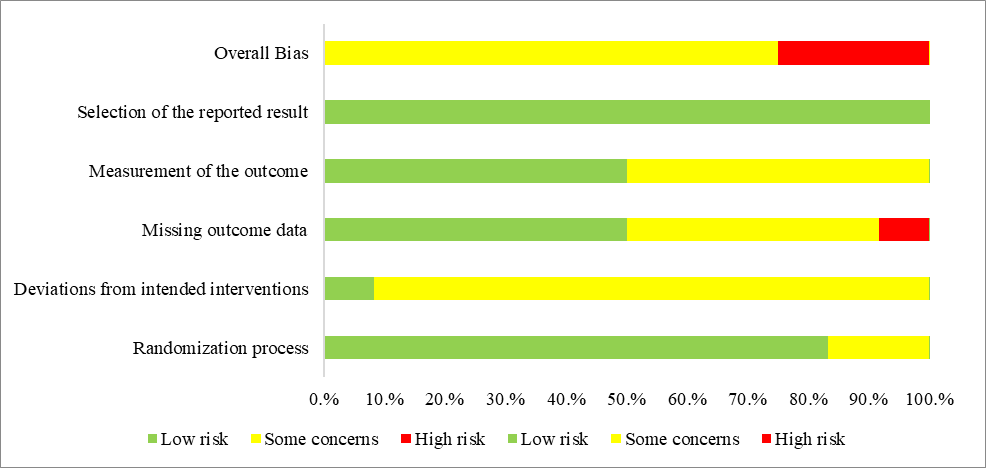


# Figure S25: Risk of bias assessment at study level regarding HOMA-IR.

# Figure S26: Risk of bias assessment at domain level regarding HOMA-IR.


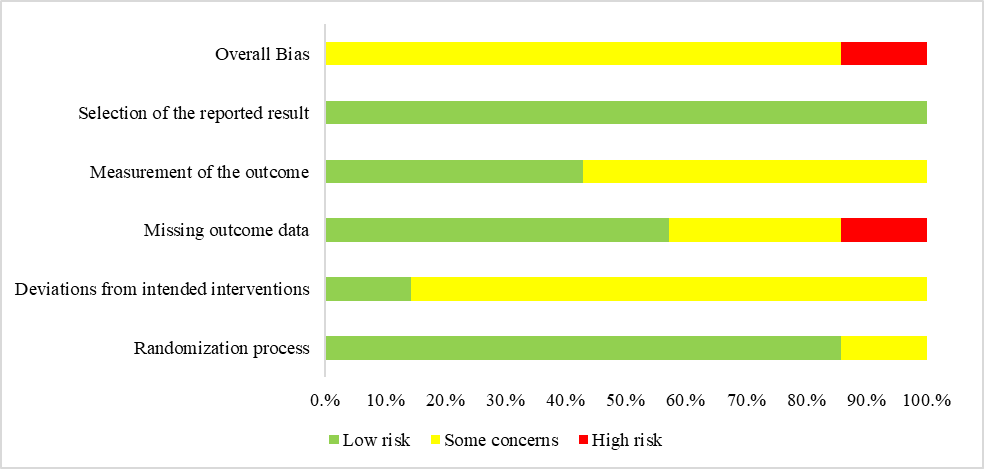


# Figure S27: Risk of bias assessment at study level regarding fasting insulin level.

# Figure S28: Risk of bias assessment at domain level regarding fasting insulin level.


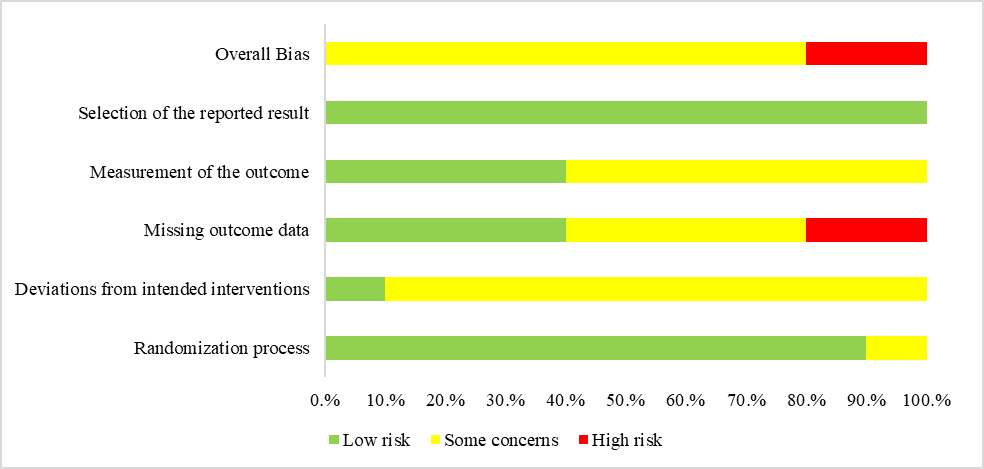


# Figure S29: Risk of bias assessment at study level regarding fasting blood glucose level.

# Figure S30: Risk of bias assessment at domain level regarding fasting blood glucose level.


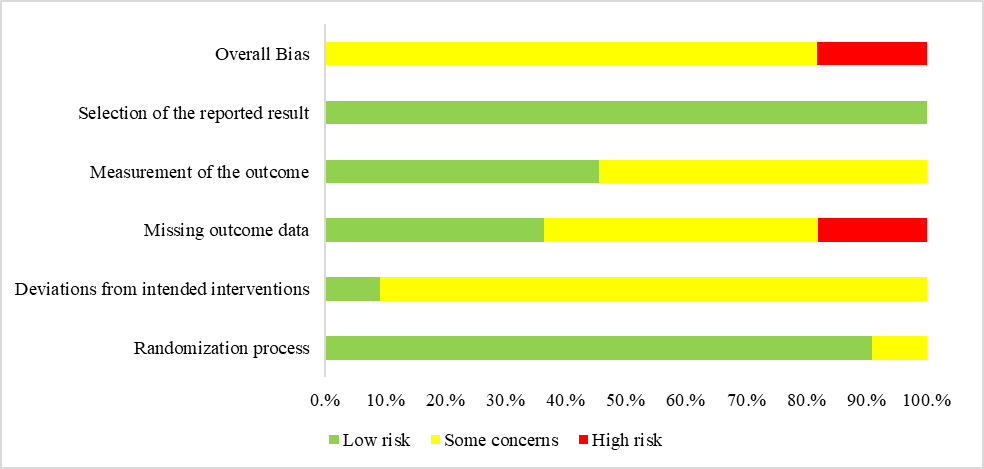


# Figure S31: Risk of bias assessment at study level regarding total testosterone level.

# Figure S32: Risk of bias assessment at domain level regarding total testosterone level.


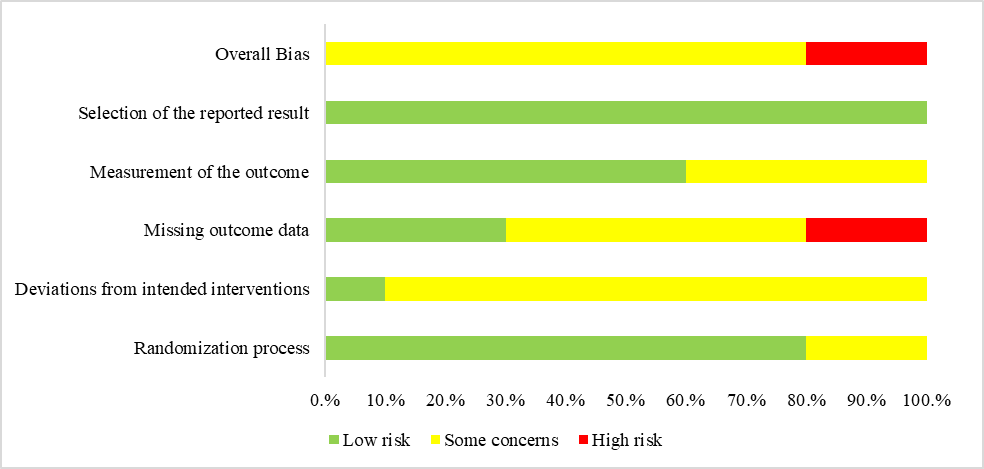


# Figure S33: Risk of bias assessment at study level regarding LDL level.

# Figure S34: Risk of bias assessment at domain level regarding LDL level.


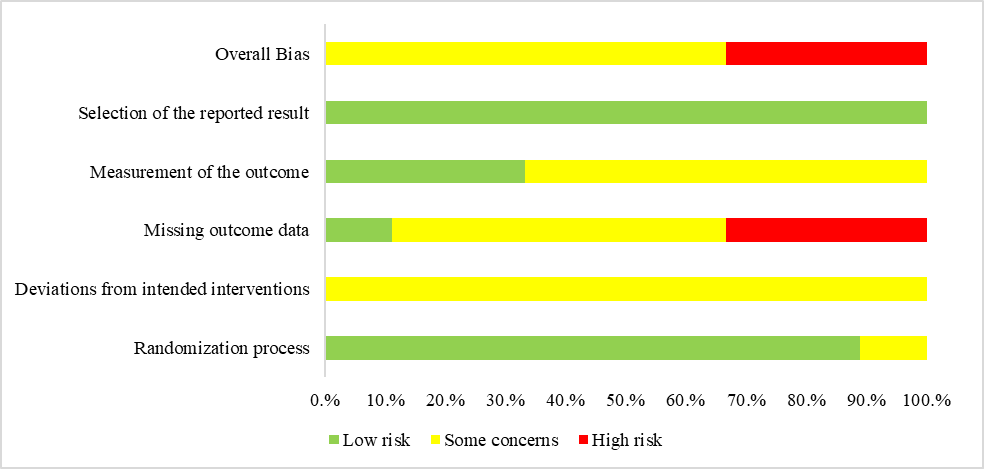


# Figure S35: Risk of bias assessment at study level regarding HDL level.

# Figure S36: Risk of bias assessment at domain level regarding HDL level.


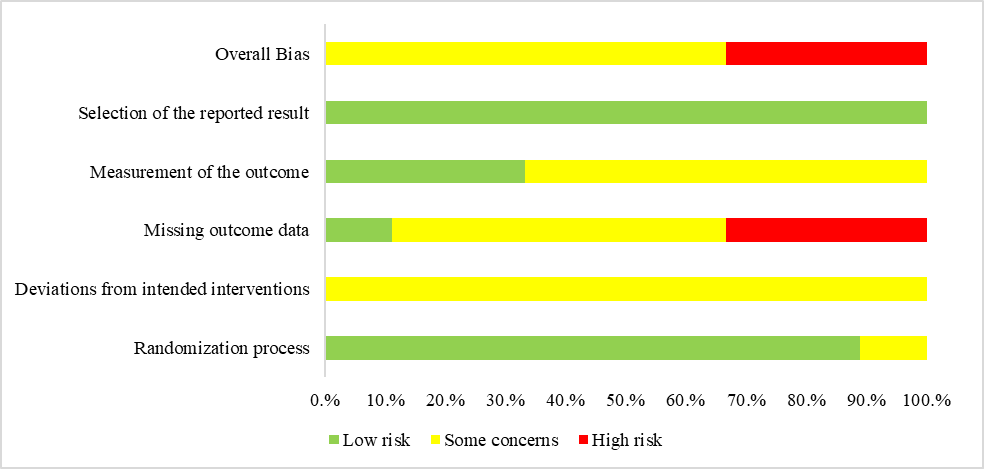


# Figure S37: Risk of bias assessment at study level regarding triglyceride level.

# Figure S38: Risk of bias assessment at domain level regarding triglyceride level.


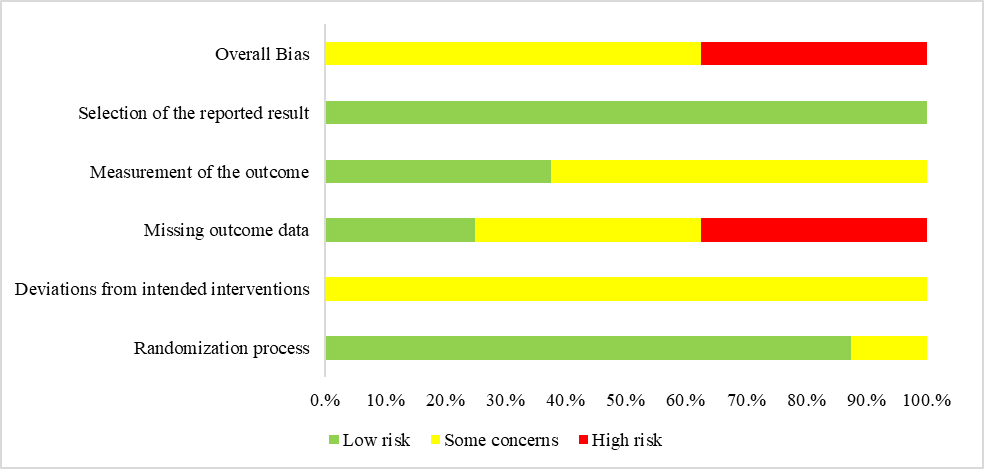


# Figure S39: Risk of bias assessment at study level regarding cholesterol level.

# Figure S40: Risk of bias assessment at domain level regarding cholesterol level.


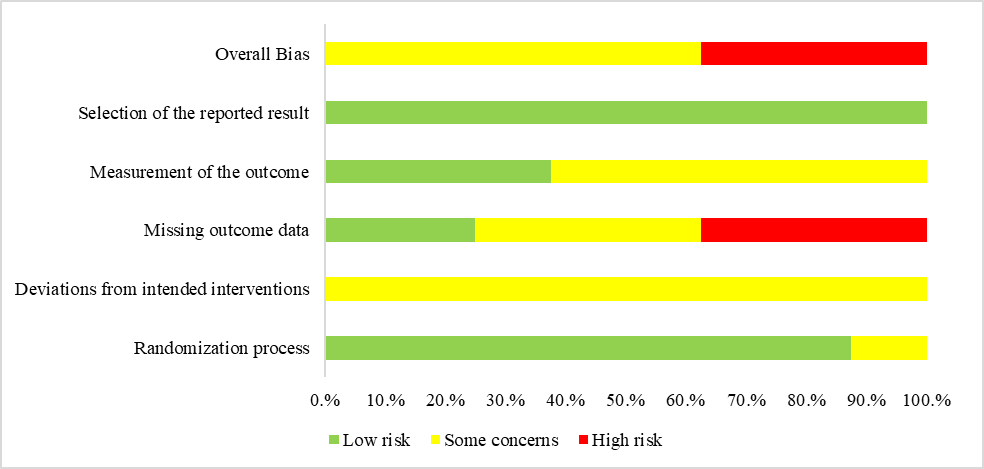


# Table S13: Assessment of certainty of evidence regarding BMI.

1: Regarding risk of bias (RoB) in indirect comparisons, overall RoBwas assessed by the study carrying highest RoBin the same comparison.; 2: Based on the sample size calculations of included studies or if it was not reported, 700 patient was defined as the minimum sample sizeforeachstudy; 3: The probability of the small study could not be assessed by evaluating funnel plot asymmetry visually and by Egger’s test(followingPuhan’srecommendations)due to the presence of too many direct comparisons containing a single study. No automatic downgrading was performed.4: Node splitting could not be performed due to the geometry of the networks, as a result inconsistency could not be tested. No automatic downgrading was performed.; 5: Indirectnesscannot be judged in a star-shapednetwork.

DASH, Dietary approach stop hypertension; Low-carb, Low- carbohydrate; High-P, High-Protein diet; Low-GI, Low-Glycemic Index diet; Low-calorie + M, Low-calorie diet plus metformin; diet

# Table S14: Assessment of certainty of evidence regarding weight.

1: Regarding risk of bias (RoB) in indirect comparisons, overall RoBwas assessed by the study carrying highest RoBin the same comparison.; 2: Based on the sample size calculations of included studies or if it was not reported, 700 patient was defined as the minimum sample sizeforeachstudy; 3: The probability of the small study could not be assessed by evaluating funnel plot asymmetry visually and by Egger’s test(followingPuhan’srecommendations)due to the presence of too many direct comparisons containing a single study. No automatic downgrading was performed.4: Node splitting could not be performed due to the geometry of the networks, as a result inconsistency could not be tested. No automatic downgrading was performed.; 5: Indirectnesscannot be judged in a star-shapednetwork.

DASH, Dietary approach stop hypertension; Low-carb, Low- carbohydrate; High-P, High-Protein diet; Low-GI, Low-Glycemic Index diet; Low-calorie + M, Low-calorie diet plus metformin; diet

# Table S15: Assessment of certainty of evidence regarding HOMA-IR.

1: Regarding risk of bias (RoB) in indirect comparisons, overall RoBwas assessed by the study carrying highest RoBin the same comparison.; 2: Based on the sample size calculations of included studies or if it was not reported, 700 patient was defined as the minimum sample sizeforeachstudy; 3: The probability of the small study could not be assessed by evaluating funnel plot asymmetry visually and by Egger’s test(followingPuhan’srecommendations)due to the presence of too many direct comparisons containing a single study. No automatic downgrading was performed.4: Node splitting could not be performed due to the geometry of the networks, as a result inconsistency could not be tested. No automatic downgrading was performed.; 5: Indirectnesscannot be judged in a star-shapednetwork.

DASH, Dietary approach stop hypertension; Low-carb, Low- carbohydrate; High-P, High-Protein diet; Low-GI, Low-Glycemic Index diet; Low-calorie + M, Low-calorie diet plus metformin; diet

Table S16: Assessment of certainty of evidence regarding fasting insulin level. (continue)

1: Regarding risk of bias (RoB) in indirect comparisons, overall RoBwas assessed by the study carrying highest RoBin the same comparison.; 2: Based on the sample size calculations of included studies or if it was not reported, 700 patient was defined as the minimum sample sizeforeachstudy; 3: The probability of the small study could not be assessed by evaluating funnel plot asymmetry visually and by Egger’s test(followingPuhan’srecommendations)due to the presence of too many direct comparisons containing a single study. No automatic downgrading was performed.4: Node splitting could not be performed due to the geometry of the networks, as a result inconsistency could not be tested. No automatic downgrading was performed.; 5: Indirectnesscannot be judged in a star-shapednetwork.

DASH, Dietary approach stop hypertension; Low-carb, Low- carbohydrate; High-P, High-Protein diet; Low-GI, Low-Glycemic Index diet; Low-calorie + M, Low-calorie diet plus metformin; diet

# Table S17: Assessment of certainty of evidence regarding fasting insulin level.

# Table S18: Assessment of certainty of evidence regarding fasting blood glucose level.

1: Regarding risk of bias (RoB) in indirect comparisons, overall RoBwas assessed by the study carrying highest RoBin the same comparison.; 2: Based on the sample size calculations of included studies or if it was not reported, 700 patient was defined as the minimum sample sizeforeachstudy; 3: The probability of the small study could not be assessed by evaluating funnel plot asymmetry visually and by Egger’s test(followingPuhan’srecommendations)due to the presence of too many direct comparisons containing a single study. No automatic downgrading was performed.4: Node splitting could not be performed due to the geometry of the networks, as a result inconsistency could not be tested. No automatic downgrading was performed.; 5: Indirectnesscannot be judged in a star-shapednetwork.

DASH, Dietary approach stop hypertension; Low-carb, Low- carbohydrate; High-P, High-Protein diet; Low-GI, Low-Glycemic Index diet; Low-calorie + M, Low-calorie diet plus metformin; diet

# Table S19: Assessment of certainty of evidence regarding total testosterone level.

1: Regarding risk of bias (RoB) in indirect comparisons, overall RoBwas assessed by the study carrying highest RoBin the same comparison.; 2: Based on the sample size calculations of included studies or if it was not reported, 700 patient was defined as the minimum sample sizeforeachstudy; 3: The probability of the small study could not be assessed by evaluating funnel plot asymmetry visually and by Egger’s test(followingPuhan’srecommendations)due to the presence of too many direct comparisons containing a single study. No automatic downgrading was performed.4: Node splitting could not be performed due to the geometry of the networks, as a result inconsistency could not be tested. No automatic downgrading was performed.; 5: Indirectnesscannot be judged in a star-shapednetwork.

DASH, Dietary approach stop hypertension; Low-carb, Low- carbohydrate; High-P, High-Protein diet; Low-GI, Low-Glycemic Index diet; Low-calorie + M, Low-calorie diet plus metformin; diet

# Table S20: Assessment of certainty of evidence regarding LDL level.

1: Regarding risk of bias (RoB) in indirect comparisons, overall RoBwas assessed by the study carrying highest RoBin the same comparison.; 2: Based on the sample size calculations of included studies or if it was not reported, 700 patient was defined as the minimum sample sizeforeachstudy; 3: The probability of the small study could not be assessed by evaluating funnel plot asymmetry visually and by Egger’s test(followingPuhan’srecommendations)due to the presence of too many direct comparisons containing a single study. No automatic downgrading was performed.4: Node splitting could not be performed due to the geometry of the networks, as a result inconsistency could not be tested. No automatic downgrading was performed.; 5: Indirectnesscannot be judged in a star-shapednetwork.

DASH, Dietary approach stop hypertension; Low-carb, Low- carbohydrate; High-P, High-Protein diet; Low-GI, Low-Glycemic Index diet; Low-calorie + M, Low-calorie diet plus metformin; diet

# Table S21: Assessment of certainty of evidence regarding HDL level.

1: Regarding risk of bias (RoB) in indirect comparisons, overall RoBwas assessed by the study carrying highest RoBin the same comparison.; 2: Based on the sample size calculations of included studies or if it was not reported, 700 patient was defined as the minimum sample sizeforeachstudy; 3: The probability of the small study could not be assessed by evaluating funnel plot asymmetry visually and by Egger’s test(followingPuhan’srecommendations)due to the presence of too many direct comparisons containing a single study. No automatic downgrading was performed.4: Node splitting could not be performed due to the geometry of the networks, as a result inconsistency could not be tested. No automatic downgrading was performed.; 5: Indirectnesscannot be judged in a star-shapednetwork.

DASH, Dietary approach stop hypertension; Low-carb, Low- carbohydrate; High-P, High-Protein diet; Low-GI, Low-Glycemic Index diet; Low-calorie + M, Low-calorie diet plus metformin; diet

# Table S22: Assessment of certainty of evidence regarding triglyceride level.

1: Regarding risk of bias (RoB) in indirect comparisons, overall RoBwas assessed by the study carrying highest RoBin the same comparison.; 2: Based on the sample size calculations of included studies or if it was not reported, 700 patient was defined as the minimum sample sizeforeachstudy; 3: The probability of the small study could not be assessed by evaluating funnel plot asymmetry visually and by Egger’s test(followingPuhan’srecommendations)due to the presence of too many direct comparisons containing a single study. No automatic downgrading was performed.4: Node splitting could not be performed due to the geometry of the networks, as a result inconsistency could not be tested. No automatic downgrading was performed.; 5: Indirectnesscannot be judged in a star-shapednetwork.

DASH, Dietary approach stop hypertension; Low-carb, Low- carbohydrate; High-P, High-Protein diet; Low-GI, Low-Glycemic Index diet; Low-calorie + M, Low-calorie diet plus metformin; diet

# Table S23: Assessment of certainty of evidence regarding cholesterol level.

1: Regarding risk of bias (RoB) in indirect comparisons, overall RoBwas assessed by the study carrying highest RoBin the same comparison.; 2: Based on the sample size calculations of included studies or if it was not reported, 700 patient was defined as the minimum sample sizeforeachstudy; 3: The probability of the small study could not be assessed by evaluating funnel plot asymmetry visually and by Egger’s test(followingPuhan’srecommendations)due to the presence of too many direct comparisons containing a single study. No automatic downgrading was performed.4: Node splitting could not be performed due to the geometry of the networks, as a result inconsistency could not be tested. No automatic downgrading was performed.; 5: Indirectnesscannot be judged in a star-shapednetwork.

DASH, Dietary approach stop hypertension; Low-carb, Low- carbohydrate; High-P, High-Protein diet; Low-GI, Low-Glycemic Index diet; Low-calorie + M, Low-calorie diet plus metformin; diet

# Figure S41: Results from investigations of inconsistency regarding BMI. The figure shows the deviances from consistency and the inconsistency models. Consistency model assumes that the evidence derived from direct and indirect estimates should be in agreement.


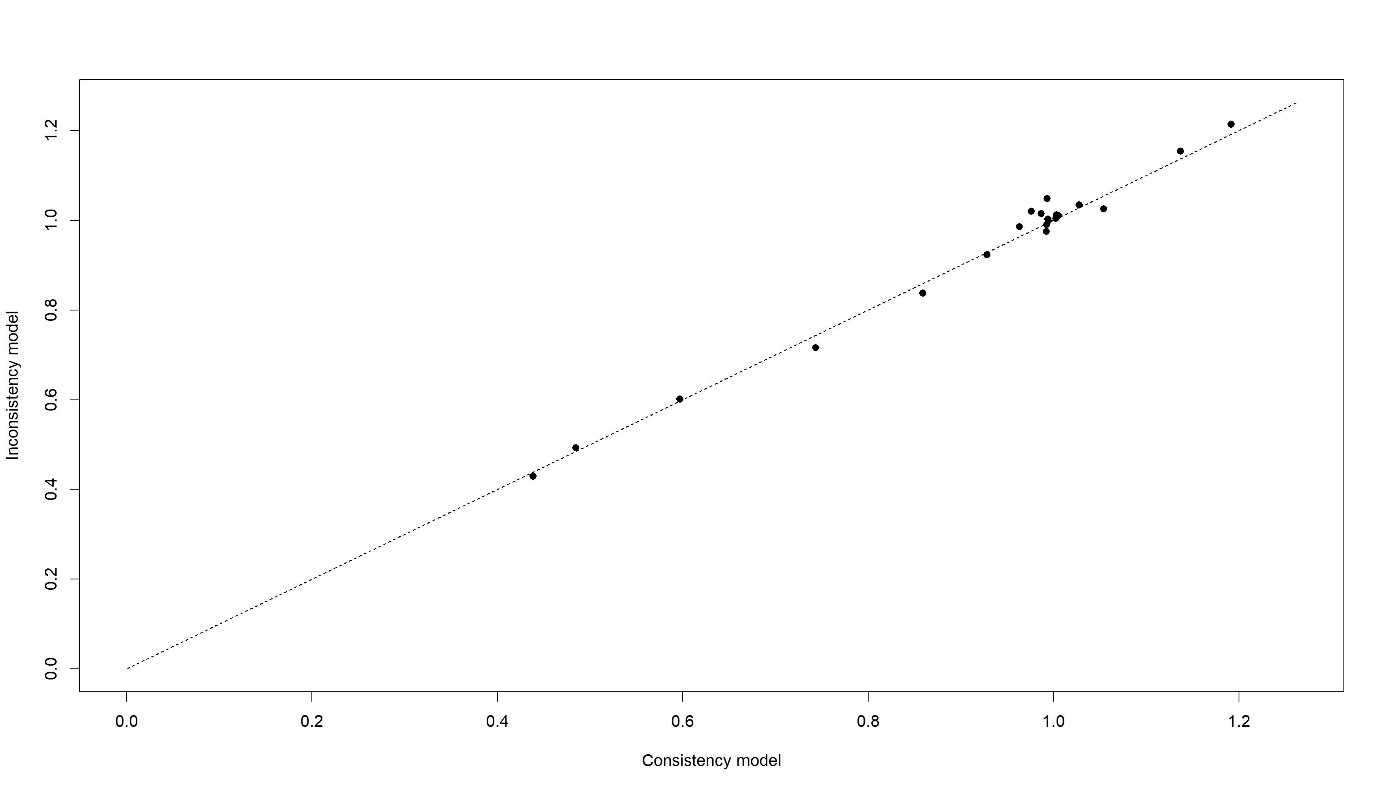


# Figure S42: Results from investigations of inconsistency regarding weight. The figure shows the deviances from consistency and the inconsistency models. Consistency model assumes that the evidence derived from direct and indirect estimates should be in agreement.


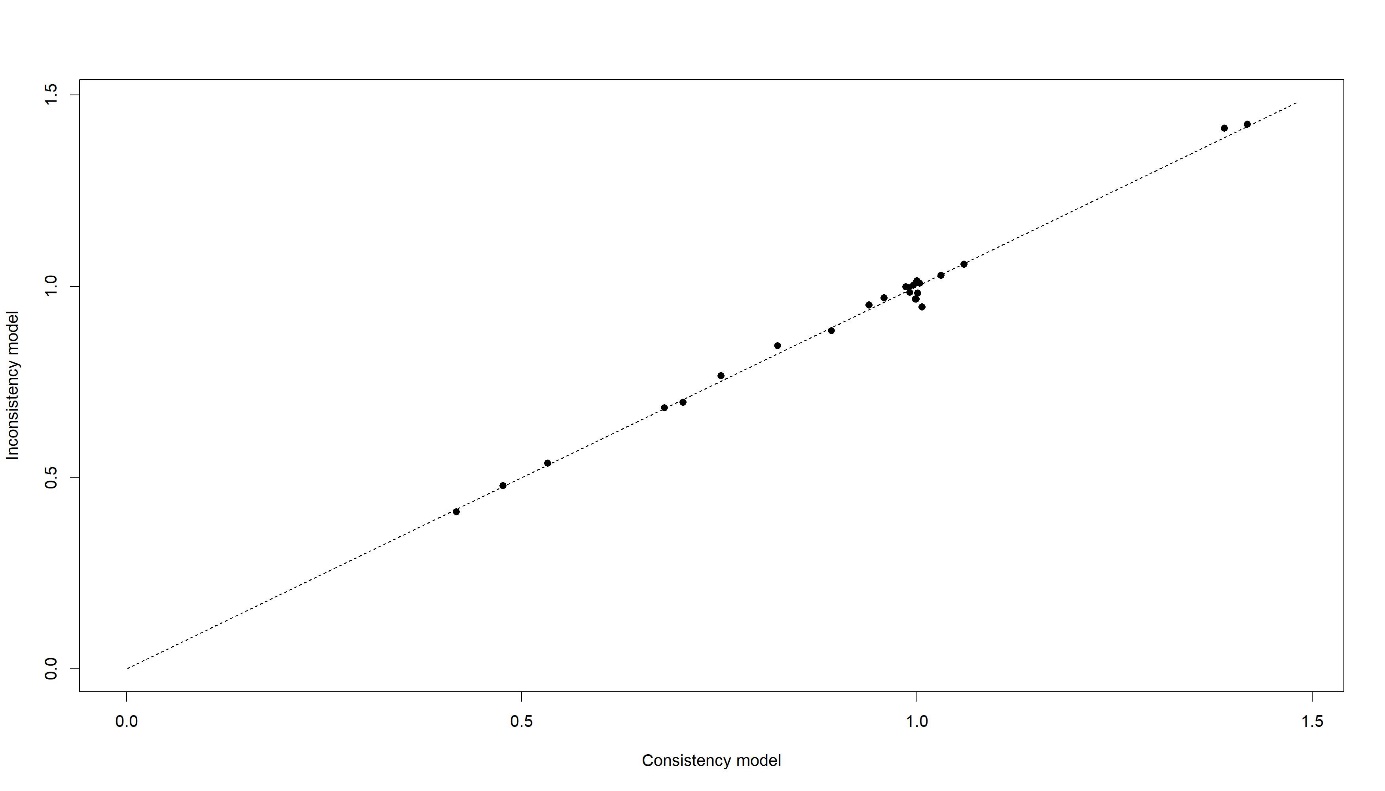


# Figure S43: Results from investigations of inconsistency regarding HOMA-IR. The figure shows the deviances from consistency and the inconsistency models. Consistency model assumes that the evidence derived from direct and indirect estimates should be in agreement.


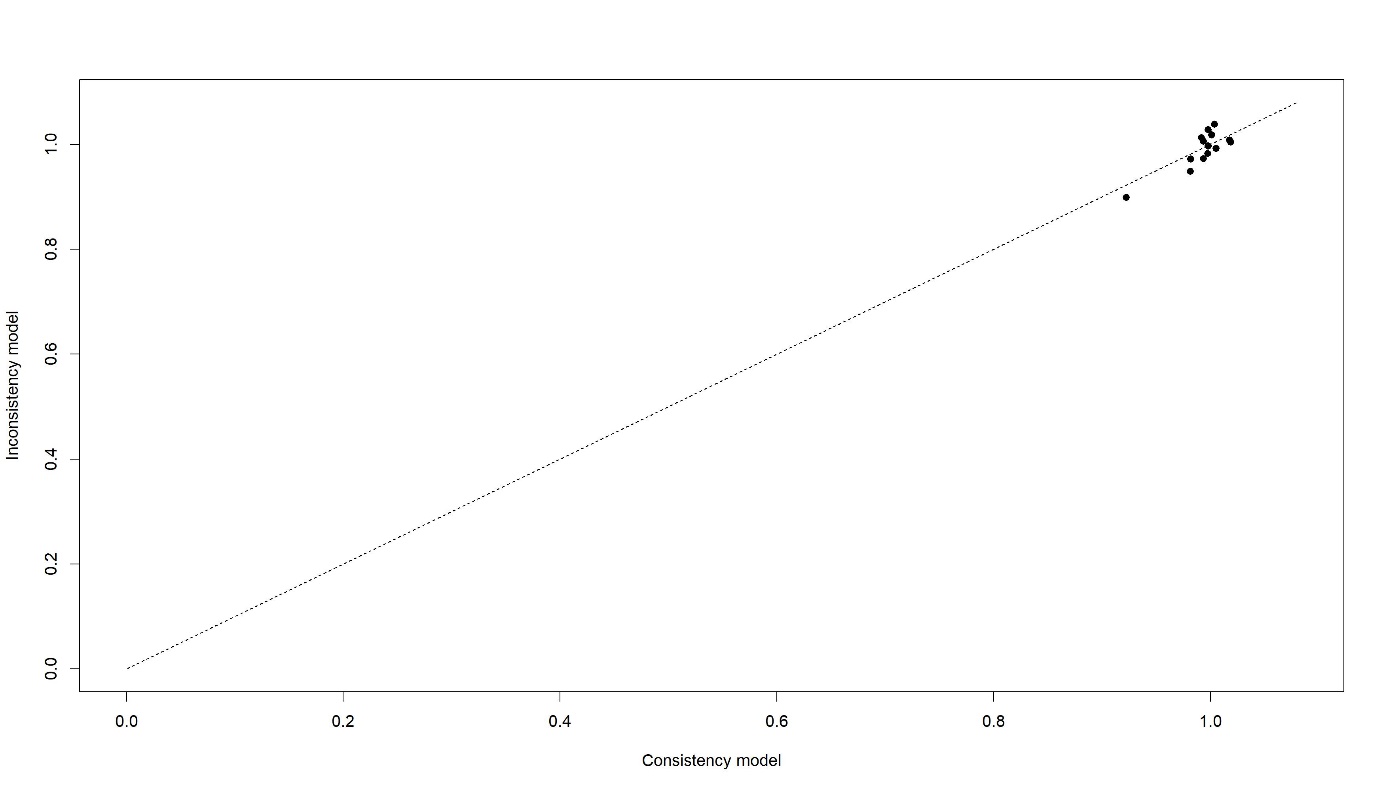


# Figure S43: Results from investigations of inconsistency regarding fasting insulin level. The figure shows the deviances from consistency and the inconsistency models. Consistency model assumes that the evidence derived from direct and indirect estimates should be in agreement.


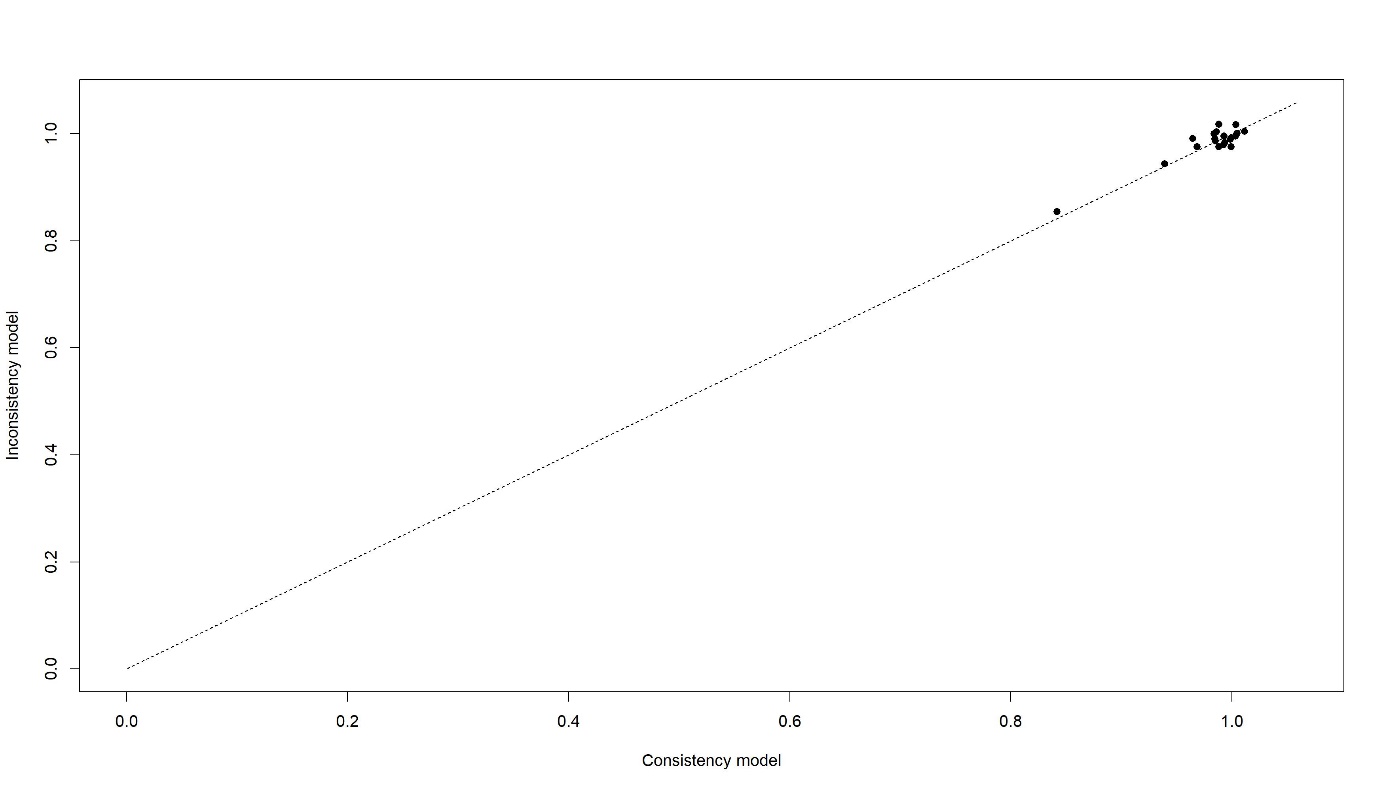


# Figure S44: Results from investigations of inconsistency regarding fasting blood glucose level. The figure shows the deviances from consistency and the inconsistency models. Consistency model assumes that the evidence derived from direct and indirect estimates should be in agreement.


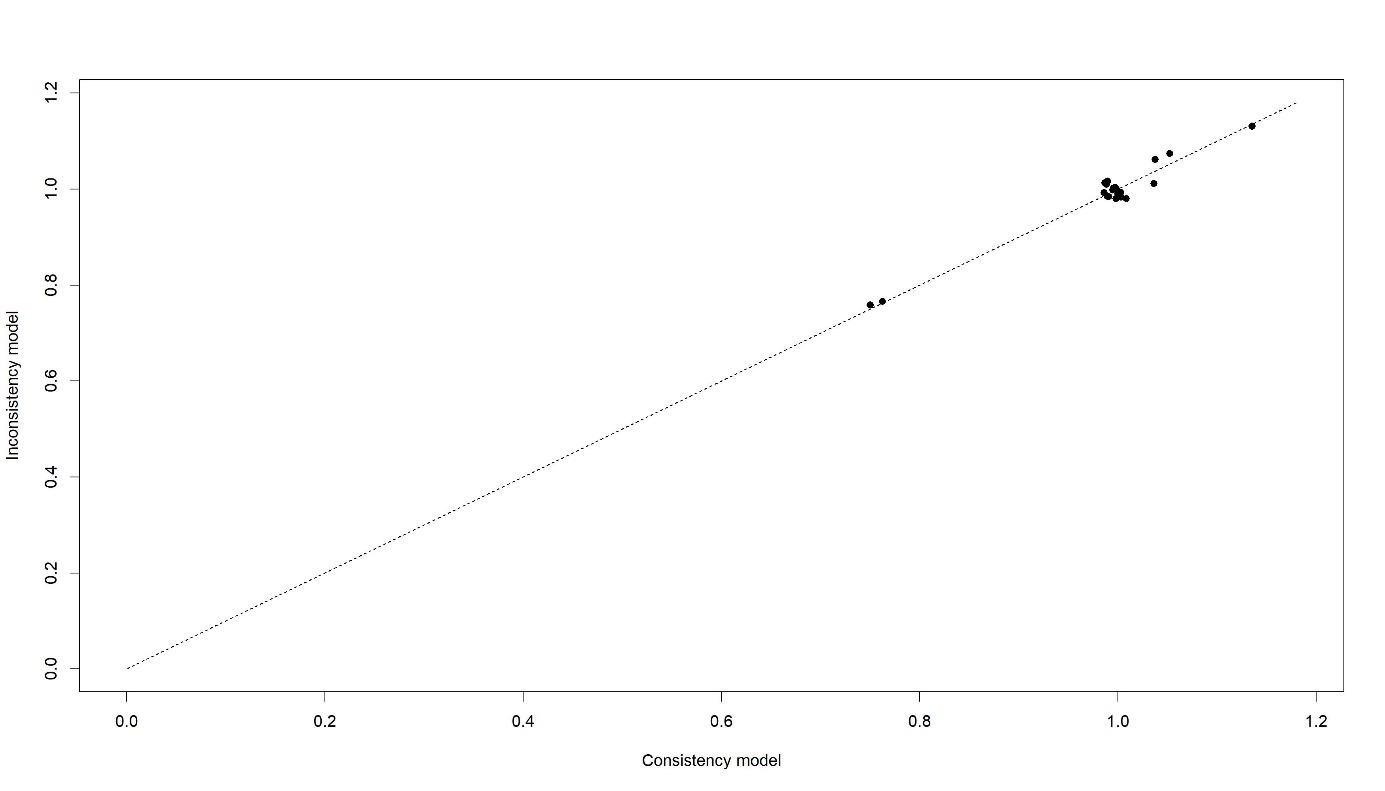


# Figure S45: Results from investigations of inconsistency regarding total testosterone level. The figure shows the deviances from consistency and the inconsistency models. Consistency model assumes that the evidence derived from direct and indirect estimates should be in agreement.


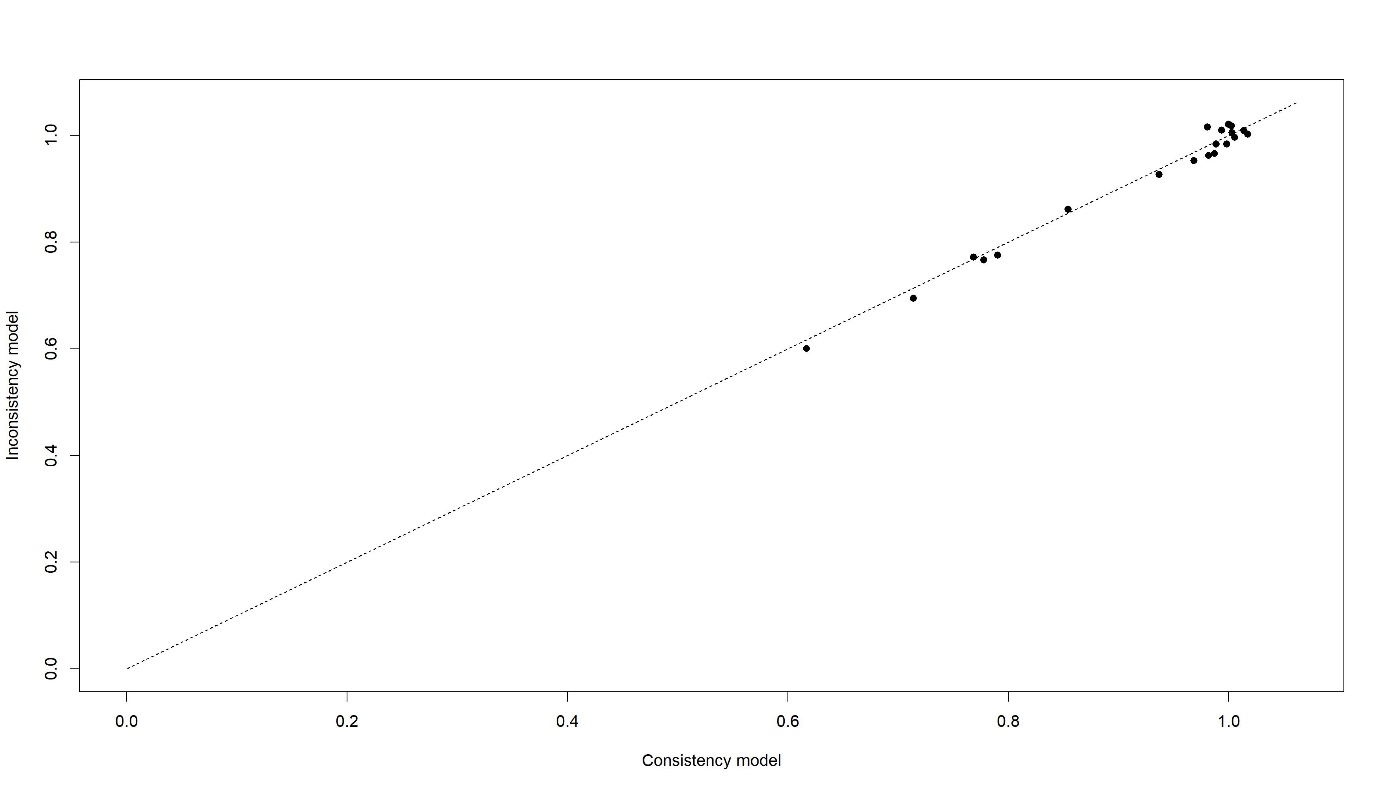


# Figure S46: Results from investigations of inconsistency regarding LDL level. The figure shows the deviances from consistency and the inconsistency models. Consistency model assumes that the evidence derived from direct and indirect estimates should be in agreement.


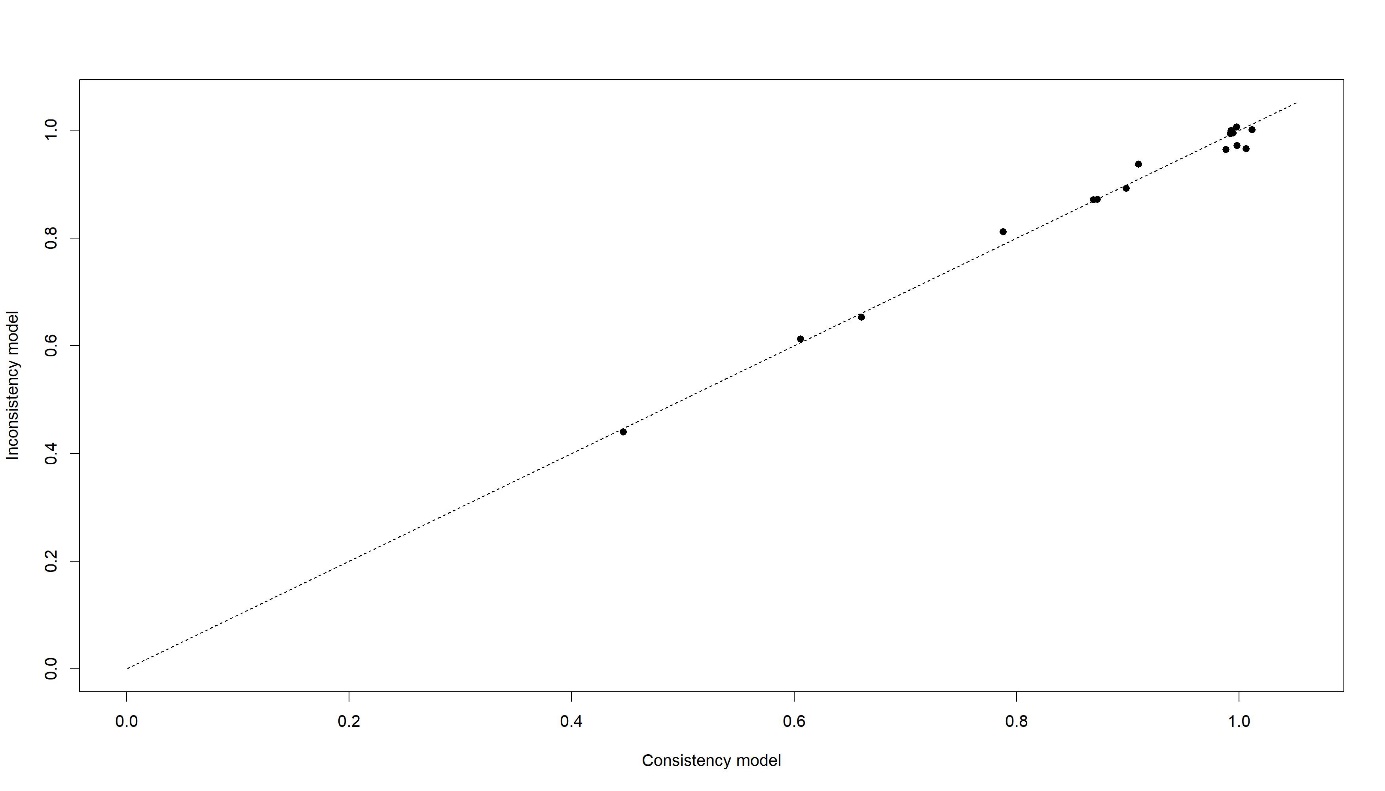


# Figure S47: Results from investigations of inconsistency regarding HDL level. The figure shows the deviances from consistency and the inconsistency models. Consistency model assumes that the evidence derived from direct and indirect estimates should be in agreement.


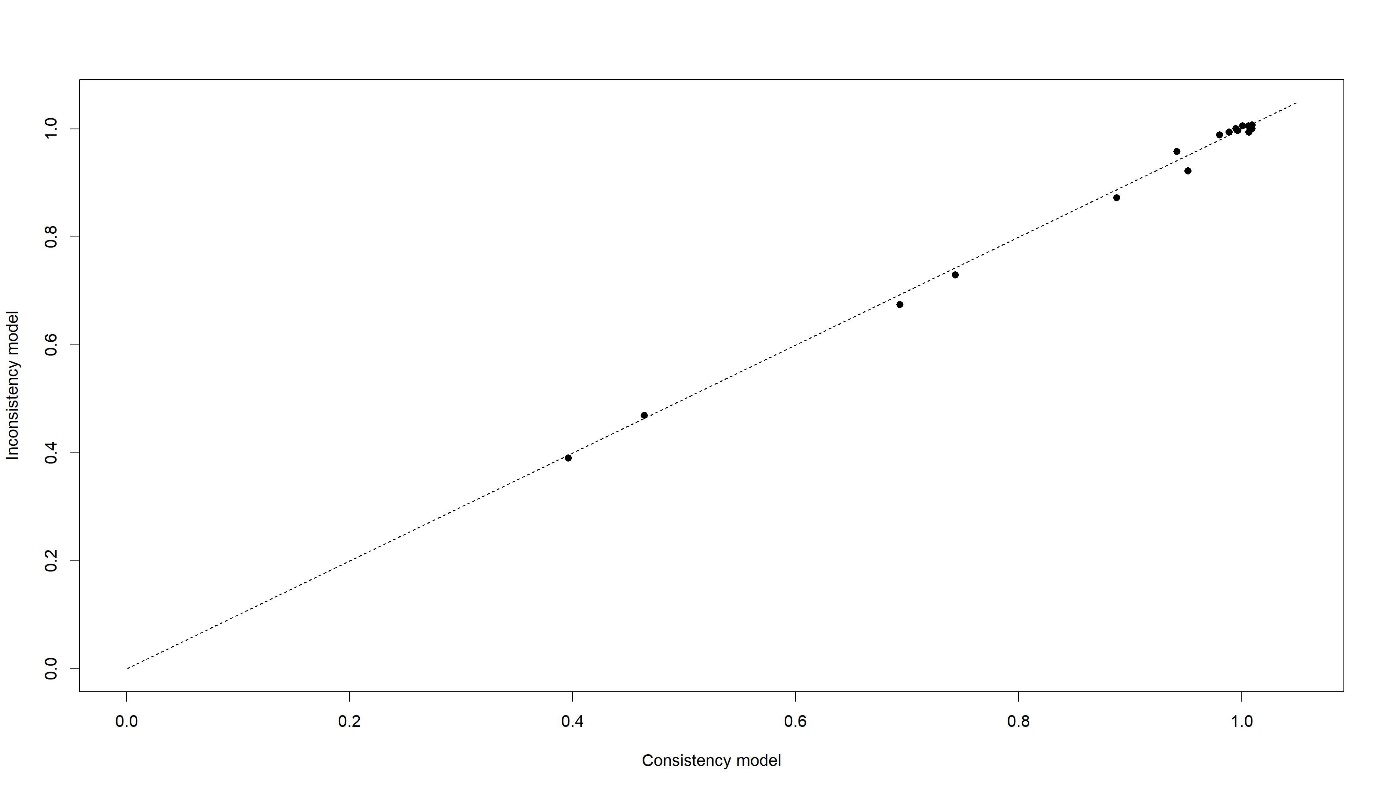


# Figure S48: Results from investigations of inconsistency regarding triglyceride level. The figure shows the deviances from consistency and the inconsistency models. Consistency model assumes that the evidence derived from direct and indirect estimates should be in agreement.


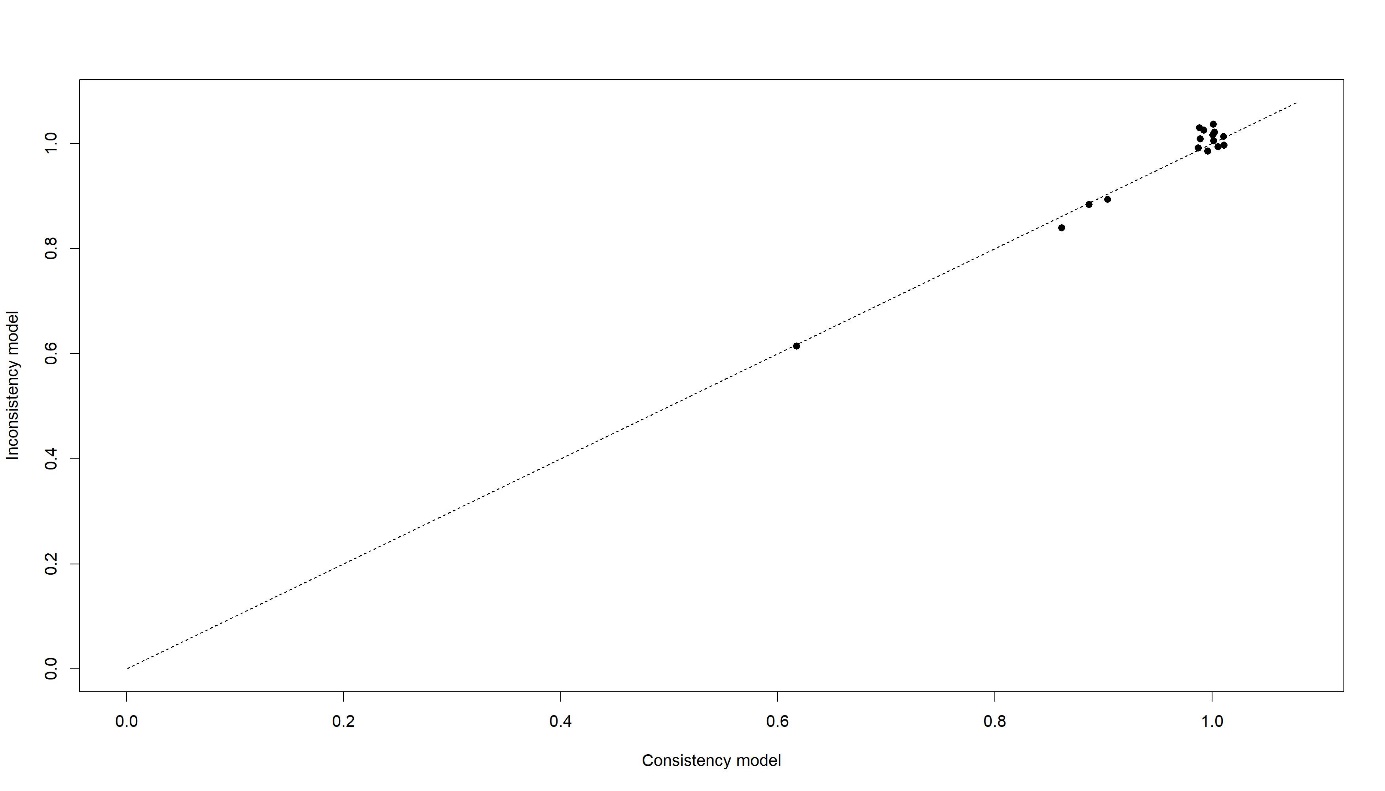


# Figure S49: Results from investigations of inconsistency regarding cholesterol level. The figure shows the deviances from consistency and the inconsistency models. Consistency model assumes that the evidence derived from direct and indirect estimates should be in agreement.


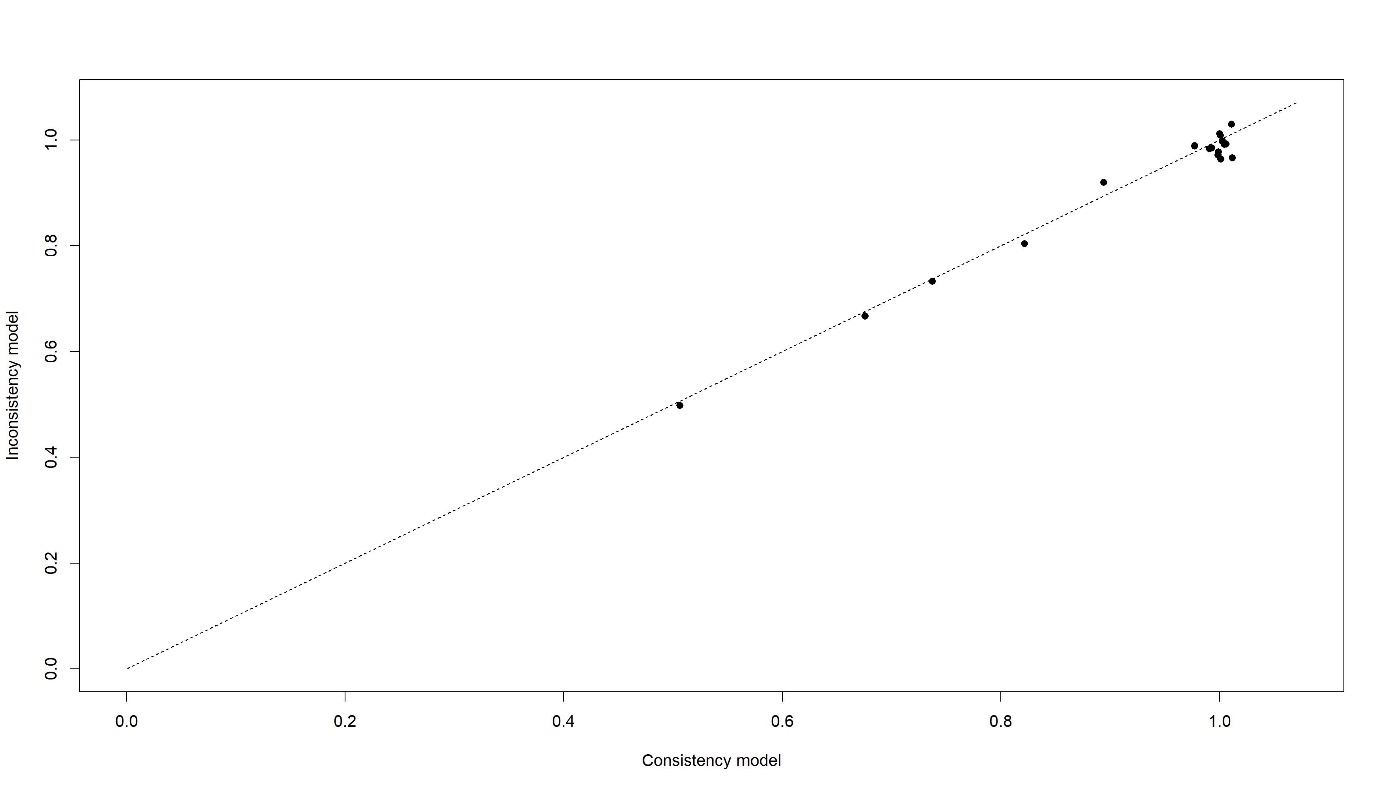

Supplement: Supplementary file 1 — Additional file 1: PRISMA NMA Checklist; Searchkey; Summary of the dietary interventions; and Rankogram, Surface under the cumulative ranking (SUCRA) curves, League table, Risk of bias assessment, Assessment of certainty of evidence, Investigations of inconsistency of all outcomes. [file 12978_2024_1758_MOESM1_ESM.docx]
